# Supplementary material for: The Genomic and Immune Landscapes of Lethal Metastatic Breast Cancer
Source: Cell Rep. 2019 May 28;27(9):2690–2708.e10. doi: 10.1016/j.celrep.2019.04.098 (PMC6546974; doi:10.1016/j.celrep.2019.04.098)
Supplement: Document S1. Figures S1–S7 [file mmc1.pdf]

Cell Reports, Volume *27*

Supplemental Information

The Genomic and Immune Landscapes

of Lethal Metastatic Breast Cancer

Leticia De Mattos-Arruda, Stephen-John Sammut, Edith M. Ross, Rachael Bashford-Rogers, Erez Greenstein, Havell Markus, Sandro Morganella, Yvonne Teng, Yosef Maruvka, Bernard Pereira, Oscar M. Rueda, Suet-Feung Chin, Tania Contente-Cuomo, Regina Mayor, Alexandra Arias, H. Raza Ali, Wei Cope, Daniel Tiezzi, Aliakbar Dariush, Tauanne Dias Amarante, Dan Reshef, Nikaoly Ciriaco, Elena Martinez-Saez, Vicente Peg, Santiago Ramon y Cajal, Javier Cortes, George Vassiliou, Gad Getz, Serena Nik-Zainal, Muhammed Murtaza, Nir Friedman, Florian Markowetz, Joan Seoane, and Carlos Caldas

Cell Reports

Supplemental Information

The genomic and immune landscapes of lethal metastatic breast cancer

Leticia De Mattos-Arruda, Stephen-John Sammut, Edith M. Ross, Rachael Bashford-Rogers, Erez Greenstein, Havell Markus, Sandro Morganella, Yvonne Teng, Yosef Maruvka, Bernard Pereira, Oscar M. Rueda, Suet-Feung Chin, Tania Contente-Cuomo, Regina Mayor, Alexandra Arias, H Raza Ali, Wei Cope, Daniel Tiezzi, Aliakbar Dariush, Tauanne Dias Amarante, Dan Reshef, Nikaoly Ciriaco, Elena Martinez-Saez, Vicente Peg, Santiago Ramon y Cajal, Javier Cortes, George Vassiliou, Gad Getz, Serena Nik-Zainal, Muhammed Murtaza, Nir Friedman, Florian Markowetz, Joan Seoane, and Carlos Caldas

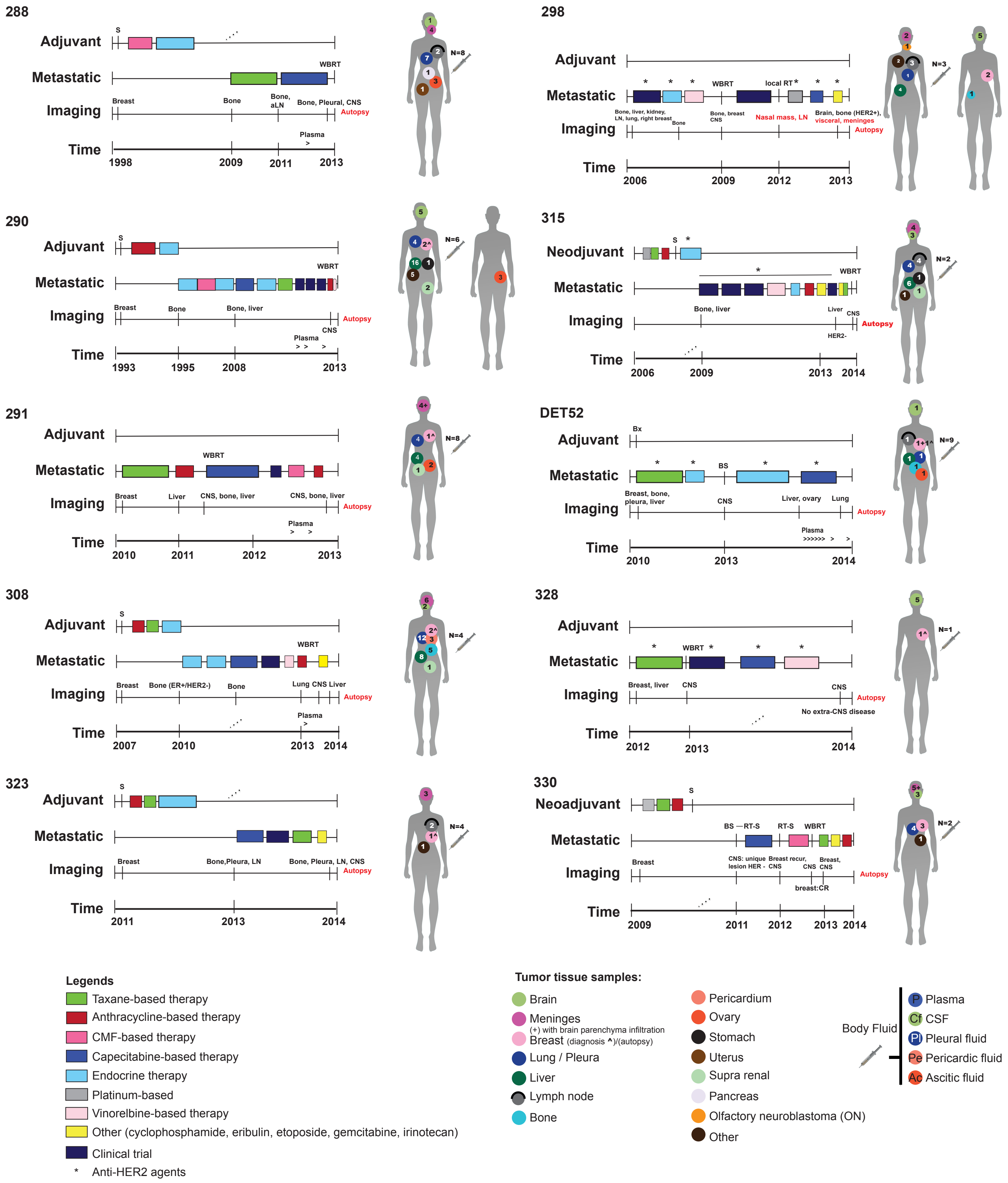

Figure S1: <u>Timelines with clinical and treatment data for all 10 cases,</u> related to Figure 1.

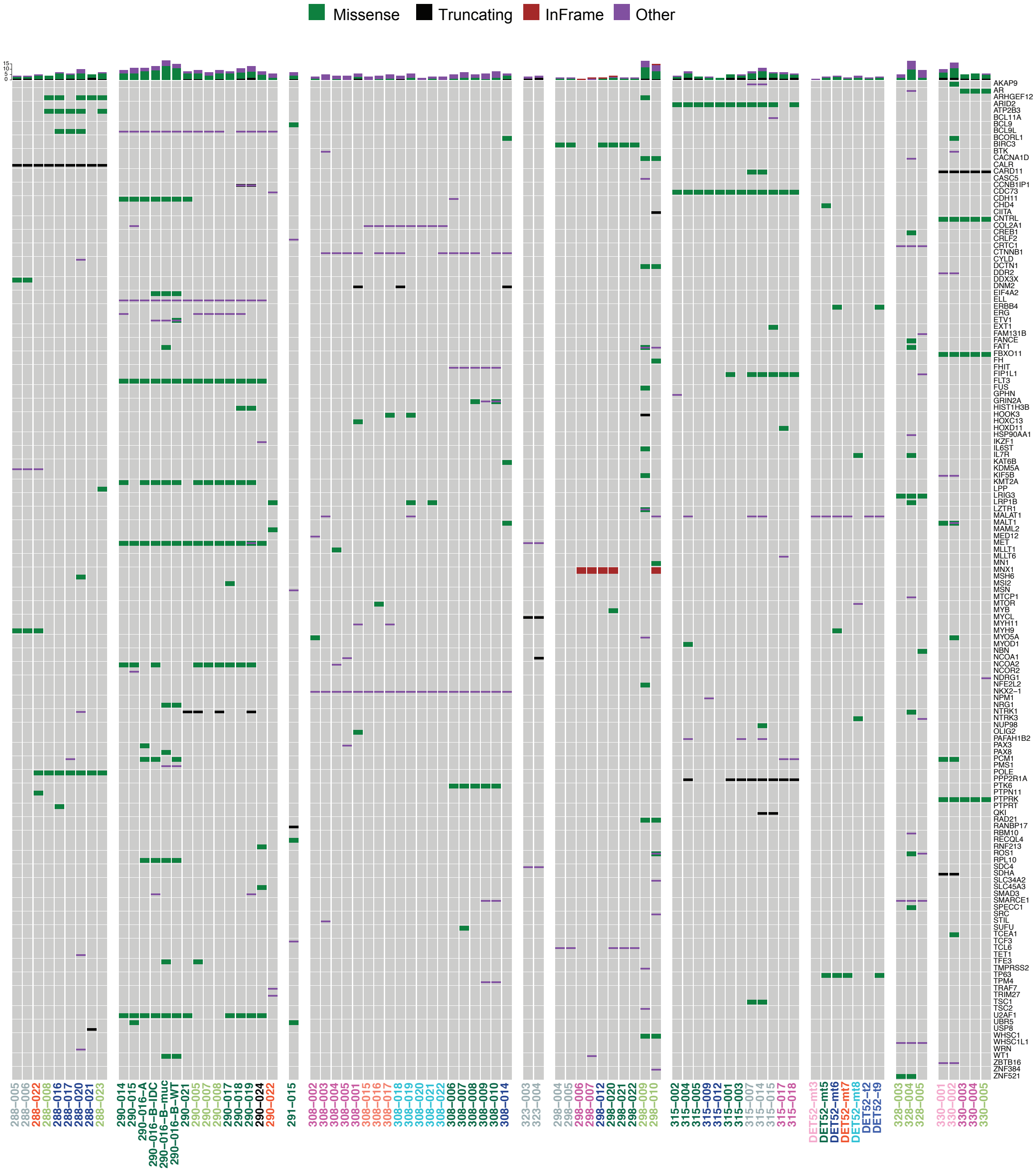

Figure S2: Oncoprint plot showing the mutations in non-breast cancer driver genes identified by WES across 85 metastases for the 10 patients, related to Figure 2.

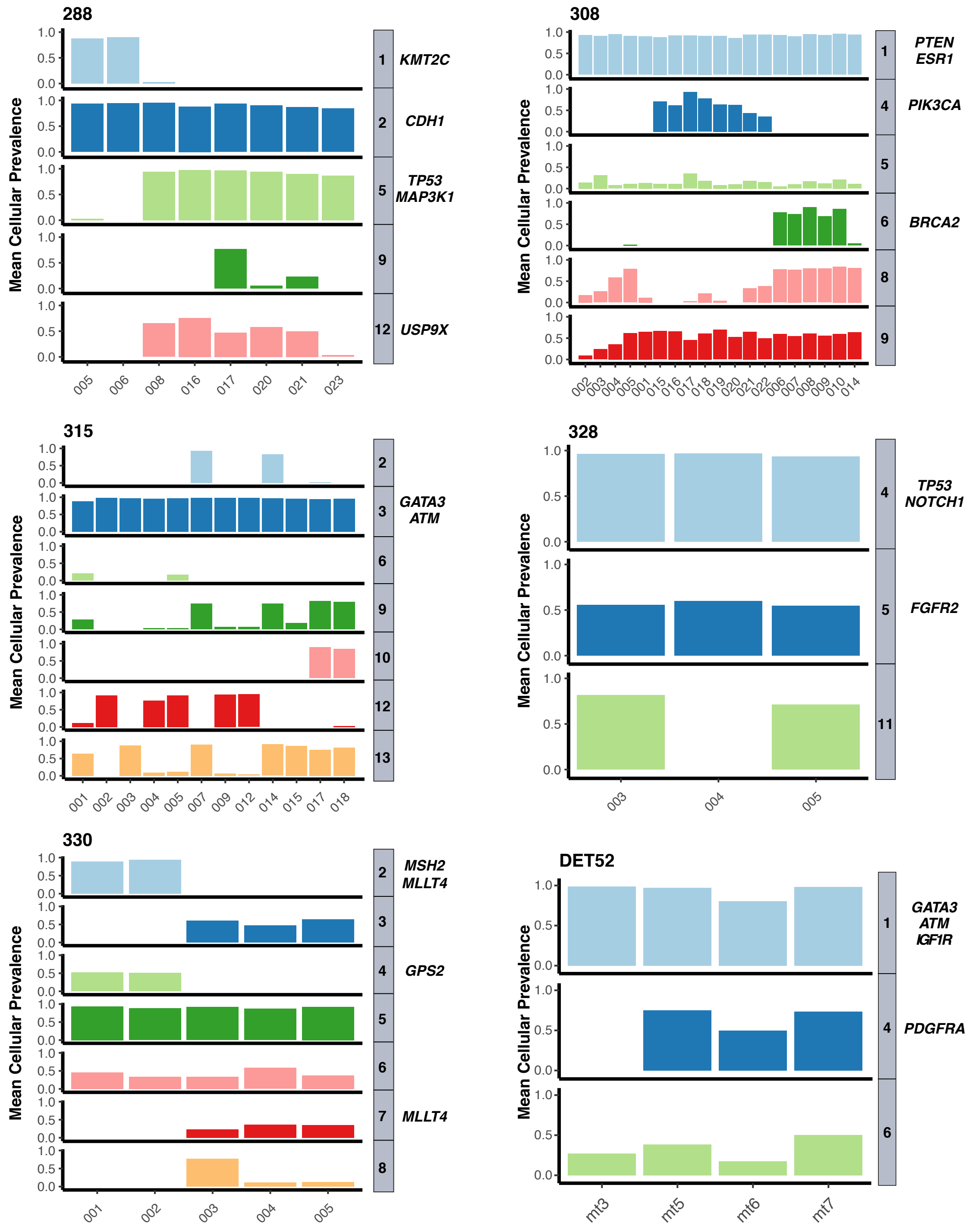

Figure S3: Mean cellular prevalence (CP) of mutation clusters identified by PyClone from WES data across metastases for 6 cases having more than two metastatic samples, related to Figure 3.

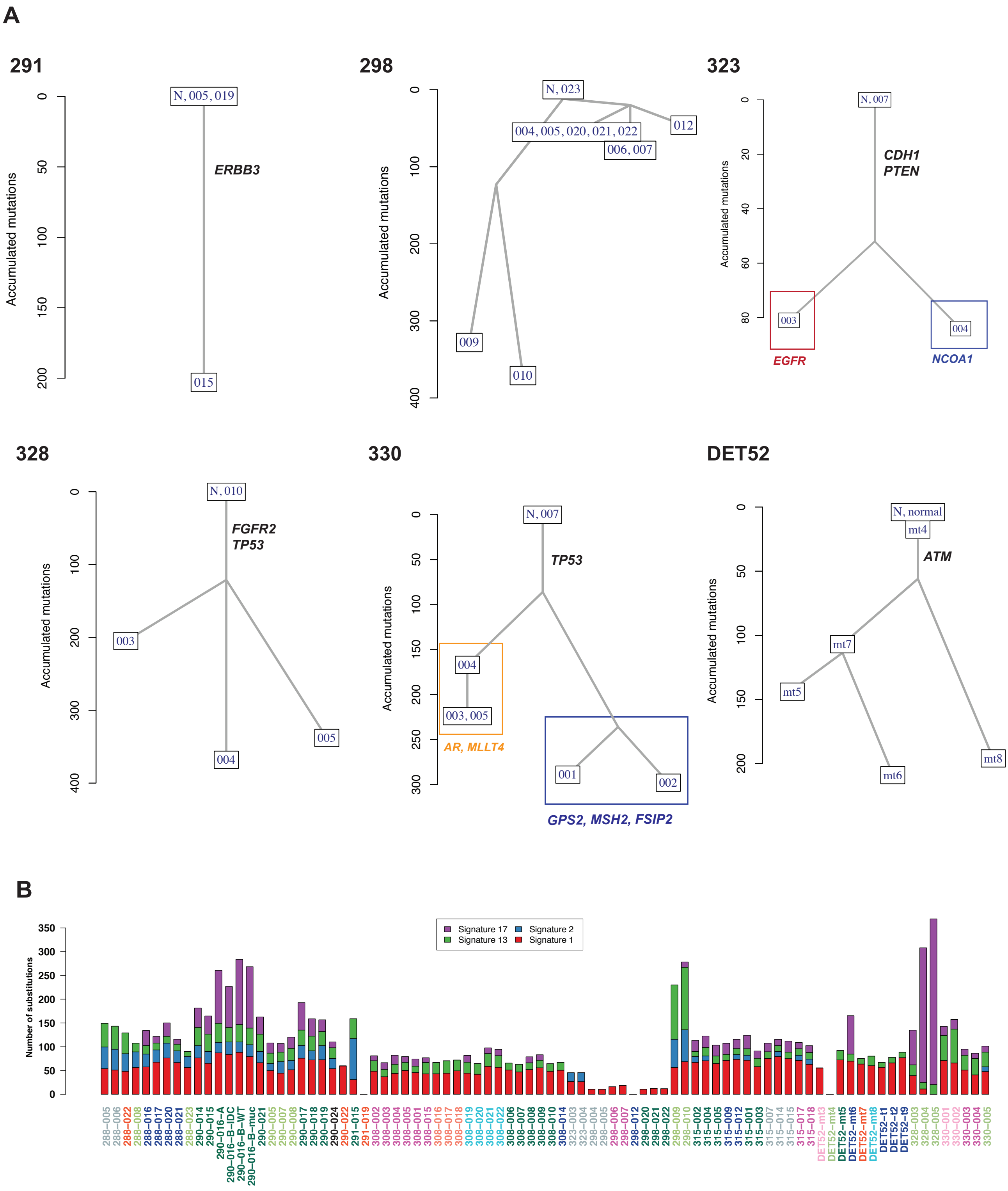

Figure S4: Phylogenetic ancestries of breast cancer metastasis, related to Figure 4. **(A)** Phylogenetic trees from OncoNEM algorithm. Cases 291, 298, 323, 328, 330, DET52. **(B)** Barplots of mutational signatures across metastasis for all cases with all mutations included. We only considered assignments with a minimum of ten mutations and with greater than 3% of the total mutational burden of each sample.

A

B

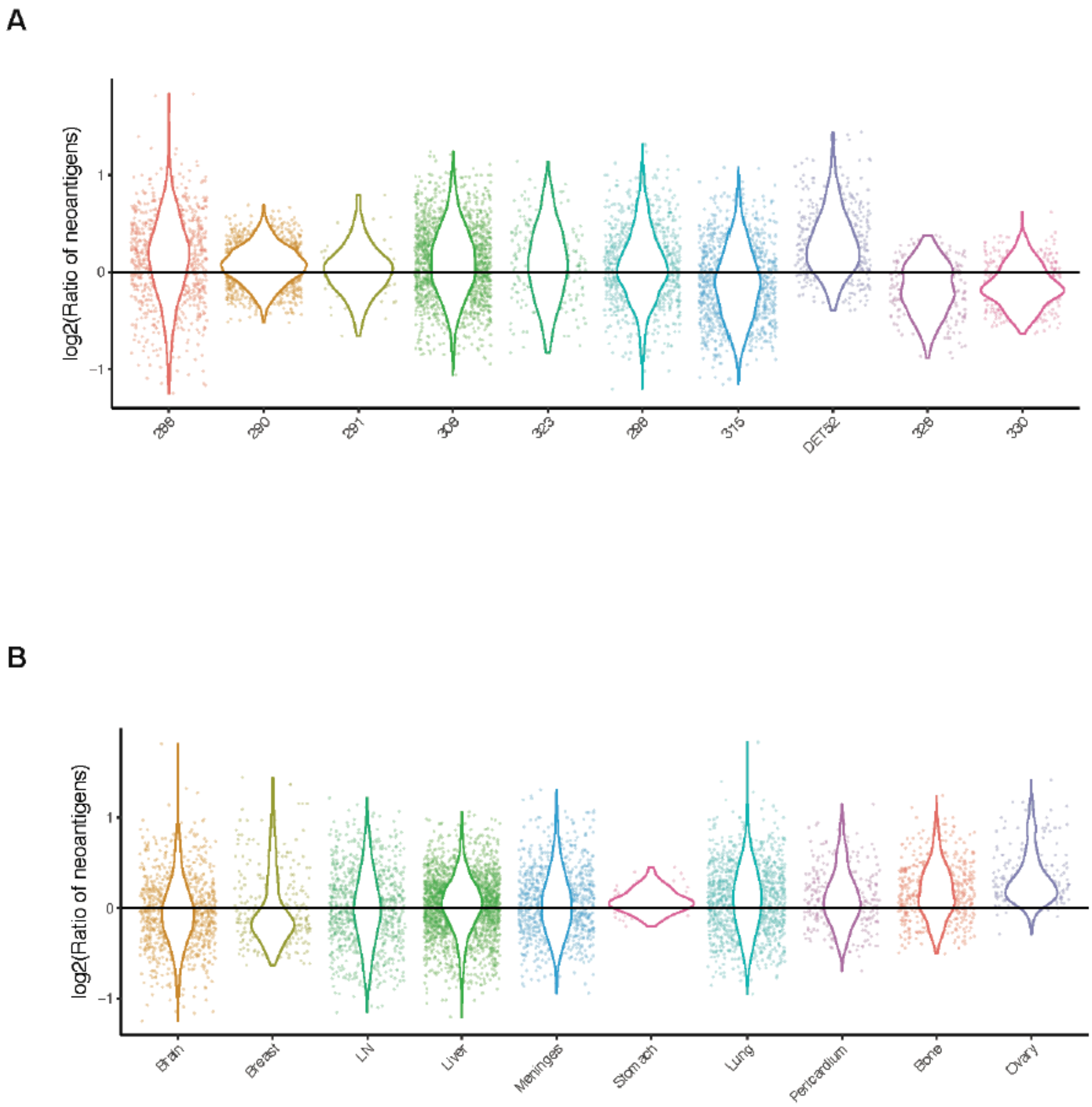

Figure S5: <u>Neo-antigen landscape across breast cancer metastasis,</u> related to Figure 5. **(A)** Violin plots of observed/expected neoantigen ratios grouped by patient. **(B)** Violin plots of observed/expected neoantigen ratios grouped by metastatic site.

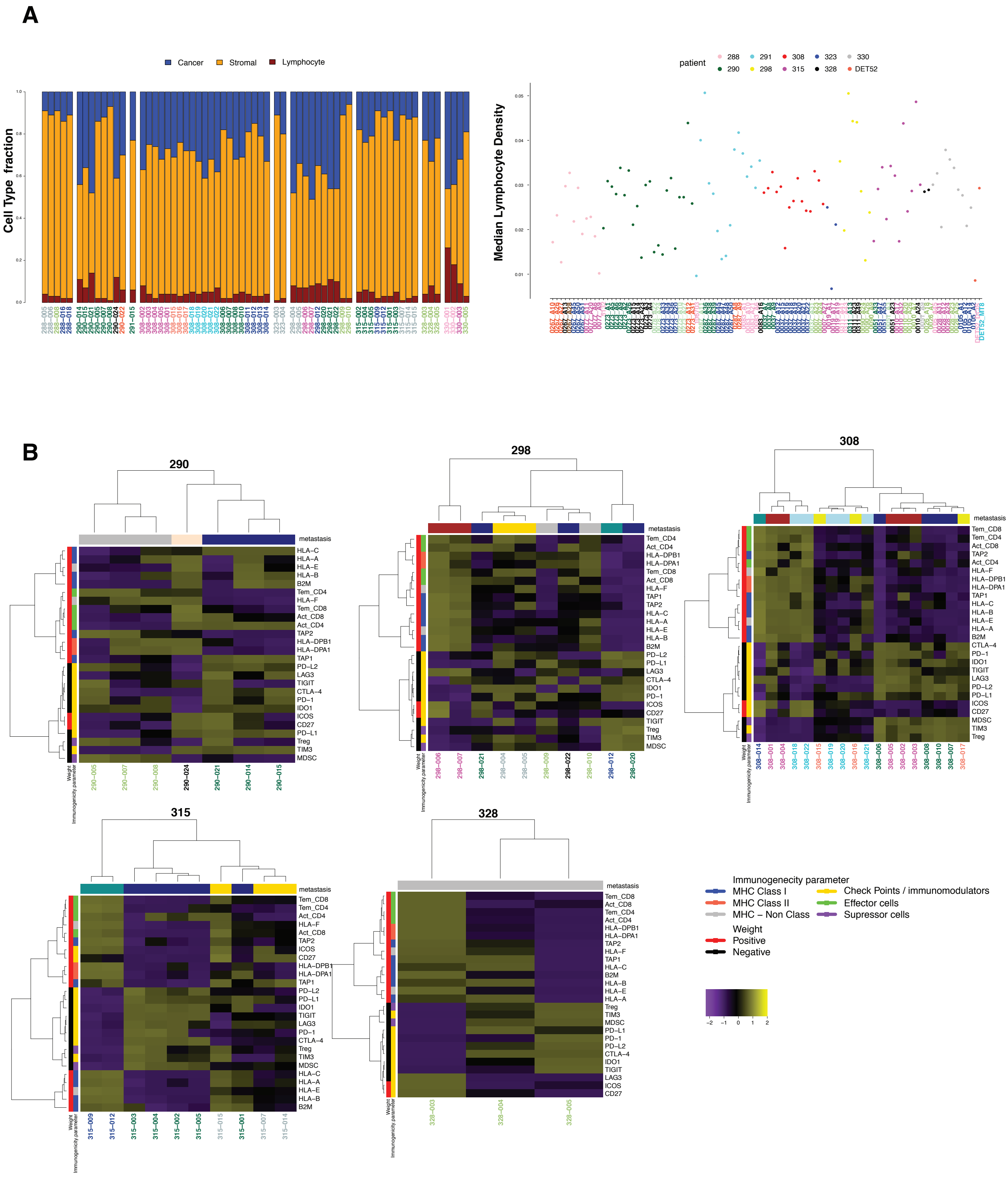

Figure S6: Tumor microenvironment is heterogeneous across metastasis, related to Figure 6. **(A)** Distribution of cancer, stromal and lymphocyte fractions across each metastasis (64 fresh frozen metastases) (left panel); Median lymphocyte density across FFPE-derived samples (primary breast and metastases) (right panel). **(B)** Heatmaps depicting two-way unsupervised hierarchical clustering of immune parameters and metastases for patients 290, 298, 308, 315, 328.

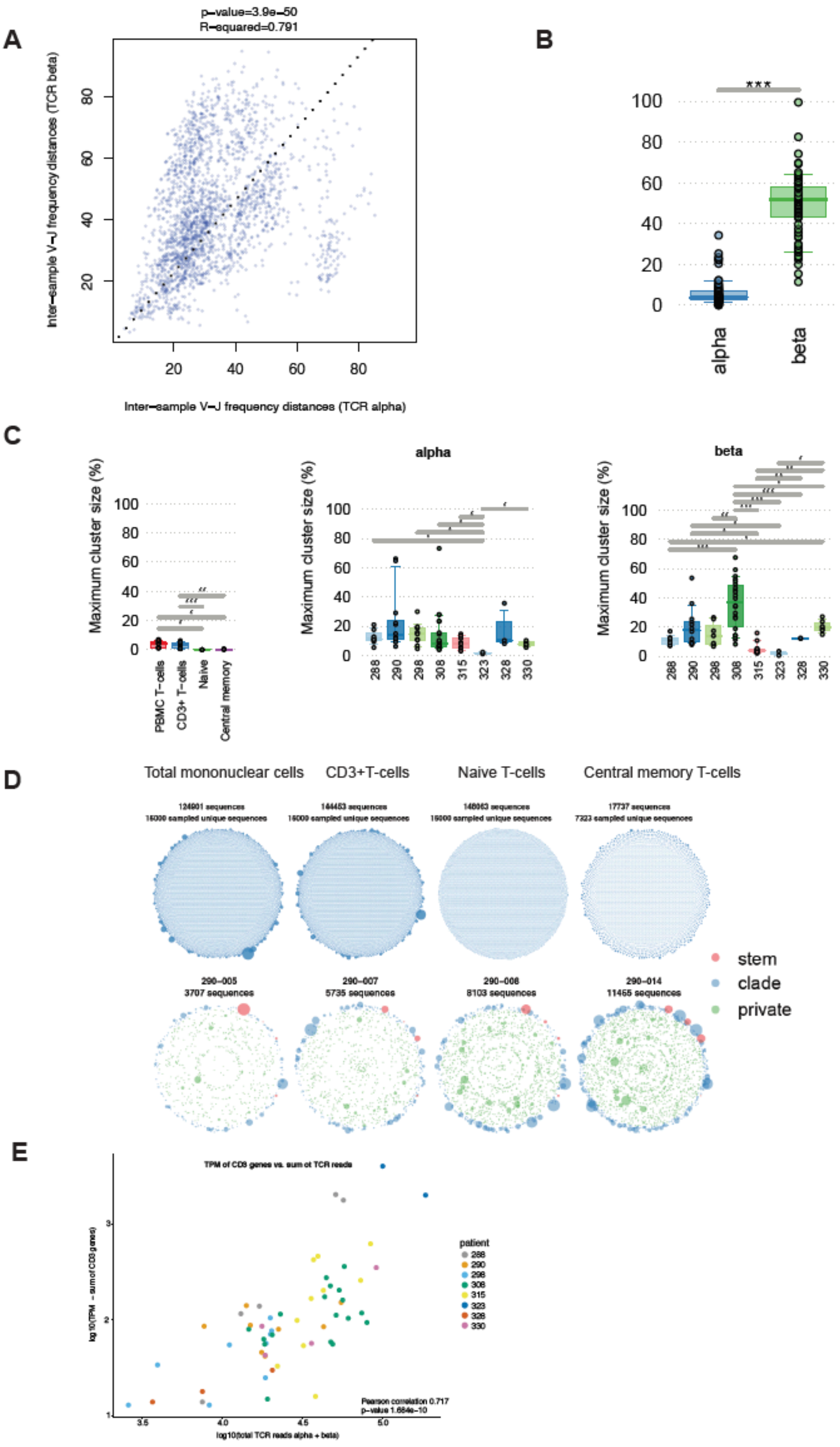

Figure S7: Analysis of TCR repertoire across metastasis, related to Figure 7. **(A)** Scatter plot of inter-sample V-J frequency distances for beta vs alpha TCR chains. **(B)** Boxplots of percentage of non-public sequences across all metastases for alpha and beta TCR chains. **(C)** Boxplots of distribution of the percentage of maximum cluster size in normal samples (beta chain) and metastases (alpha and beta chains). Significant differences between repertoire clonalities per patient for alpha and beta chains. * denotes p-values <0.05, ** denotes p-values <0.005, *** denotes p-values <0.0005 (after correcting for multiple testing). **(D)** Network plots of TCR beta chain sequences from healthy donor peripheral blood (top) and in eight metastases from case 290 (bottom). Color code indicates whether TCR sequences are stem, clade or private. Network plots for all metastases across cases where TCR sequencing was done are presented in SI7. **(E)** Correlation between the sum of the TPM values for the four genes that comprise the CD3 complex (CD3D, CD3G, CD3E and the zeta chain CD247) and the number of TCR reads.
