# Supplementary material for: The Genomic and Immune Landscapes of Lethal Metastatic Breast Cancer
Source: Cell Rep. 2019 May 28;27(9):2690–2708.e10. doi: 10.1016/j.celrep.2019.04.098 (PMC6546974; doi:10.1016/j.celrep.2019.04.098)
Supplement: Document S2. Article plus Supplemental Information [file mmc9.pdf]

Cell Reports

Article

The Genomic and Immune Landscapes of Lethal Metastatic Breast Cancer

Graphical Abstract

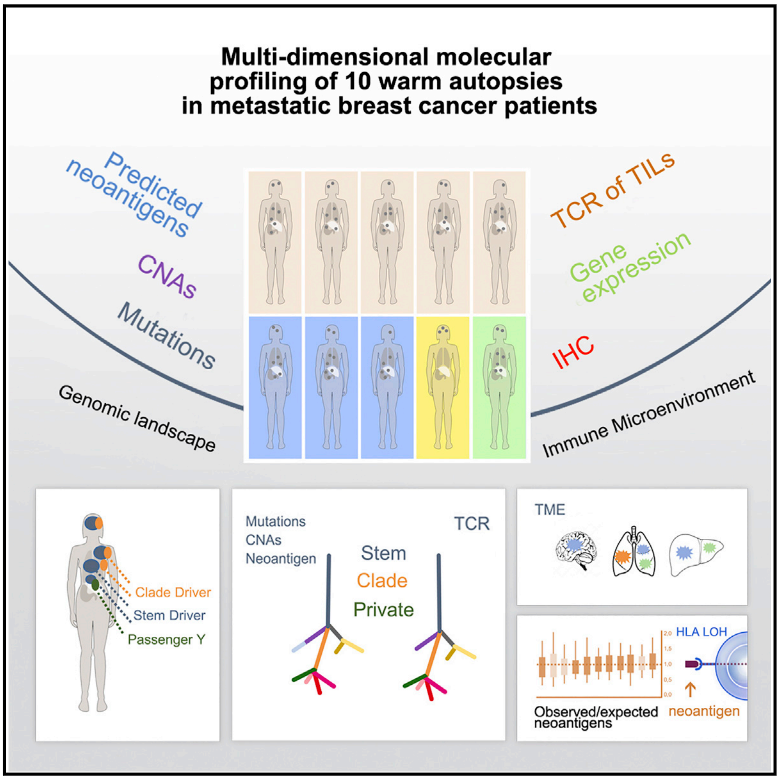

Highlights

- Genomic and transcriptomic landscapes for 10 lethal breast cancers
- Within a patient, metastases group in limited clades with shared genomic ancestry
- Tumor immune microenvironments across metastases are not uniform
- Phylogenetic trees are correlated with TIL-TCR trees across metastases

Authors

Leticia De Mattos-Arruda, Stephen-John Sammut, Edith M. Ross, ..., Florian Markowetz, Joan Seoane, Carlos Caldas

Correspondence

jseoane@vhio.net (J.S.), carlos.caldas@cruk.cam.ac.uk (C.C.)

In Brief

De Mattos-Arruda et al. profiled multiple metastases from autopsies of patients with therapy-resistant breast cancer, showing that multi-clonal spreading occurs in a small number of founder events. The analysis characterizes predicted neo-antigen landscapes, tumor microenvironments, and accumulation of HLA LOH. T cell immune responses appear to co-evolve with metastatic cancer genomes.

Check for updates

De Mattos-Arruda et al., 2019, Cell Reports *27*, 2690–2708
May 28, 2019 © 2019 The Authors.
https://doi.org/10.1016/j.celrep.2019.04.098

CellPress

OPEN
ACCESS
CellPress

Cell Reports
Article

The Genomic and Immune Landscapes of Lethal Metastatic Breast Cancer

Leticia De Mattos-Arruda,[1,2,20] Stephen-John Sammut,[1,20] Edith M. Ross,[1] Rachael Bashford-Rogers,[3] Erez Greenstein,[4] Havell Markus,[5,6] Sandro Morganella,[7] Yvonne Teng,[8] Yosef Maruvka,[9,10] Bernard Pereira,[1] Oscar M. Rueda,[1] Suet-Feung Chin,[1] Tania Contente-Cuomo,[5,6] Regina Mayor,[2,11] Alexandra Arias,[2,11] H. Raza Ali,[1] Wei Cope,[1] Daniel Tiezzi,[1] Aliakbar Dariush,[12] Tauanne Dias Amarante,[7] Dan Reshef,[4] Nikaoly Ciriaco,[13] Elena Martinez-Saez,[11,13] Vicente Peg,[11,13,14] Santiago Ramon y Cajal,[11,13,14] Javier Cortes,[2,15] George Vassiliou,[8,16,17] Gad Getz,[9,10] Serena Nik-Zainal,[7] Muhammed Murtaza,[5,6] Nir Friedman,[4] Florian Markowetz,[1] Joan Seoane,[2,11,18,21,*] and Carlos Caldas[1,19,21,22,*]

[1]Department of Oncology and Cancer Research UK Cambridge Institute, Li Ka Shing Centre, University of Cambridge, Cambridge CB2 0RE, UK
[2]Vall d'Hebron Institute of Oncology (VHIO), Vall d'Hebron University Hospital, Barcelona 08035, Spain
[3]Department of Medicine, University of Cambridge, Cambridge CB2 0QQ, UK
[4]Department of Immunology, Weizmann Institute of Science, Rehovot 76100, Israel
[5]Center for Noninvasive Diagnostics, Translational Genomics Research Institute, Phoenix, AZ 85004, USA
[6]Mayo Clinic Center for Individualized Medicine, Scottsdale, AZ, USA
[7]Department of Medical Genetics, The Clinical School, University of Cambridge, Cambridge CB2 0QQ, UK
[8]Cancer Molecular Diagnosis Laboratory, NIHR Cambridge Biomedical Research Centre, Cambridge, UK
[9]The Broad Institute, Cambridge, MA 02142, USA
[10]Massachusetts General Hospital Cancer Center and Department of Pathology, Charlestown, MA 02129, USA
[11]Spanish Biomedical Research Network Centre in Oncology (CIBERONC), Madrid, Spain
[12]Institute of Astronomy, University of Cambridge, Cambridge CB3 0HA, UK
[13]Department of Pathology, Vall d'Hebron University Hospital, 08035 Barcelona, Spain
[14]Translational Molecular Pathology, Vall d'Hebron Research Institute (VHIR), Universitat Autònoma de Barcelona, 08035 Barcelona, Spain
[15]Ramon y Cajal Hospital, 28034 Madrid, Spain
[16]Wellcome Trust Sanger Institute, Wellcome Trust Genome Campus, Hinxton, Cambridge, UK
[17]Wellcome Trust/MRC Cambridge Stem Cell Institute, Cambridge, UK
[18]Institució Catalana de Recerca i Estudis Avançats (ICREA), Barcelona 08010, Spain
[19]Breast Cancer Programme, Cancer Research UK Cambridge Cancer Centre, Cambridge CB2 2QQ, UK
[20]These authors contributed equally
[21]Senior author
[22]Lead Contact
*Correspondence: jseoane@vhio.net (J.S.), carlos.caldas@cruk.cam.ac.uk (C.C.)
https://doi.org/10.1016/j.celrep.2019.04.098

SUMMARY

The detailed molecular characterization of lethal cancers is a prerequisite to understanding resistance to therapy and escape from cancer immunoediting. We performed extensive multi-platform profiling of multi-regional metastases in autopsies from 10 patients with therapy-resistant breast cancer. The integrated genomic and immune landscapes show that metastases propagate and evolve as communities of clones, reveal their predicted neo-antigen landscapes, and show that they can accumulate HLA loss of heterozygosity (LOH). The data further identify variable tumor microenvironments and reveal, through analyses of T cell receptor repertoires, that adaptive immune responses appear to co-evolve with the metastatic genomes. These findings reveal in fine detail the landscapes of lethal metastatic breast cancer.

INTRODUCTION

The genomic characterization of large numbers of primary breast tumors has revealed significant inter-tumor heterogeneity and unraveled an increasingly refined molecular taxonomy of early breast cancer with profound implications for prognostication and therapeutic stratification (Cancer Genome Atlas, 2012; Curtis et al., 2012; Dvinge et al., 2013; Nik-Zainal et al., 2012a, 2012b, 2016). Intra-tumor genomic heterogeneity is also seen in early breast cancers, highlighting that complex clonal architectures are already present in primary tumors (Nik-Zainal et al., 2016; Pereira et al., 2016; Shah et al., 2012; Yates et al., 2015). The tumor microenvironment (TME) in primary tumors is also different and distinctive across breast cancer subtypes, in particular with regards to adaptive immunity (Ali et al., 2016a; Rooney et al., 2015). The nature of the adaptive immune response, the status of immunoediting, and the diversity of the T cell receptor (TCR) repertoire have been analyzed in some early breast cancers (Munson et al., 2016; Park et al., 2016; Wang et al., 2017), but such information for metastatic lesions is lacking.

 © 2019 The Authors.
This is an open access article under the CC BY license (http://creativecommons.org/licenses/by/4.0/).

Large-scale studies reporting the genomic and transcriptomic characterization of breast cancer metastasis (Robinson et al., 2013) and whole-genome sequencing of matched primary tumors and metastases (Yates et al., 2017) have identified targets that are enriched in metastases compared with primary tumors. Despite their size, these studies were generally limited to single metastatic samples.

Genomic evolution is seen in breast cancer metastases compared with their matched primary tumors. This was first reported in single cases (Shah et al., 2009; Ding et al., 2010). A couple of warm autopsy case reports have also revealed heterogeneity of genomic resistance mechanisms to targeted therapies across metastases (Juric et al., 2015; Murtaza et al., 2015). More recently two small autopsy studies with multiple-metastases profiling have confirmed significant inter-metastasis heterogeneity (Hoadley et al., 2016; Savas et al., 2016).

However, a comprehensive molecular analysis of lethal breast cancers, interrogating both the malignant and TME compartments and TCR repertoires, across multiple metastases in several cases is still lacking. Here we report extensive multi-platform molecular profiling (DNA sequencing, RNA sequencing, TCR sequencing, digital pathology of H&E sections, and immunohistochemistry) of multiple individual metastases from 10 warm autopsies of patients with lethal multi-therapy-resistant breast cancers. This collection allowed us to characterize the mutational and copy number aberration (CNA) landscapes across the individual metastasis to infer the clonal ancestries of metastases, assess the TME in each individual metastasis, characterize the predicted neo-antigens, and assess the TCR repertoires across metastases, providing a detailed molecular characterization of lethal breast cancers that had been subjected to multiple lines of systemic therapies.

RESULTS

Multi-site Genomic and Transcriptomic Landscapes of Lethal Metastatic Breast Cancers

We performed warm autopsies in 10 patients with metastatic breast cancer that had become resistant to multiple lines of therapy (Figure 1A; Figure S1). The cohort is fairly representative of the major subtypes: 8 were diagnosed with estrogen receptor (ER)-positive disease, and three of these were HER2-positive; one was ER-negative and HER2-positive; and one was triple-negative. In total, 181 samples from multiple metastatic sites in each patient (mean, 18.5/patient; range, 5–37) were collected and either fresh-frozen (for DNA and RNA extraction) or formalin-fixed and paraffin-embedded (FFPE). FFPE samples from the original breast surgery or diagnostic biopsy were available from 6 of the patients, and metastatic biopsies during treatment were also collected from 3 patients. The FFPE samples were used for histological and immunohistochemistry analysis and for DNA extraction. Plasma samples and a selection of body fluids, collected during the patient's life or at autopsy, were available from all 10 patients (mean, 4.7/patient; range, 1–9/patient) and used for cell-free DNA (cfDNA) extraction. Comprehensive clinical information for the patients and analyzed samples can be found in Table S1.

The genomic landscapes of breast cancers are dominated by CNAs (Ciriello et al., 2013). We used shallow whole-genome sequencing (sWGS) to obtain CNA profiles in 168 samples from the 10 cases: 122 tumor biopsies (109 metastasis at autopsy, 4 metastatic biopsies during treatment, 8 primary breast surgical or diagnostic biopsy specimens from 6 cases, and a nasopharyngeal olfactory neuroblastoma) and 46 body fluid samples (24 plasma samples, 5 ascites samples, 9 cerebrospinal fluid [CSF] samples, 7 pleural fluid samples, and 1 pericardial fluid sample) (Table S1). For 64 of these metastatic samples (from 9 of the 10 cases), we also performed RNA sequencing (RNA-seq). The combined sWGS and RNA-seq data were used to classify individual metastasis into one of the 10 genome driver-based subtypes, called integrative clusters (IntClust) (Ali et al., 2014; Curtis et al., 2012).

The tumor CNA profiles were remarkably similar across metastases in 9 of the 10 cases, and, as expected, all metastases were classified into the same IntClust (Figure 1B; SI1 in https://doi.org/10.17632/6cv77bry6m.1). An exception was case 288, an ER-positive lobular breast cancer, where, besides a 1q gain and 16q loss seen in all metastases, there were additional and mutually exclusive CNAs: amplification of 11q13/14, including *CCND1* and *PAK1*, in lymph nodes (288-005 and 288-006), the ascites fluid cell pellet, and ovaries (classified as IntClust2) and 8q and 10q amplifications in brain and lung and pleura (classified as IntClust10). These data suggest that all metastases shared a common ancestor with 1q gain and 16q loss, and early sub-clonal evolution with remarkable genomic divergence then occurred. The PAM50 intrinsic subtypes (Parker et al., 2009) were less consistent across metastases and failed to capture the clade segregation in case 288 (SI1 in https://doi.org/10.17632/6cv77bry6m.1).

We used whole-exome sequencing (WES) at a median of 132× depth, including, in each case, DNA extracted from the buffy coat as the matched germline reference to characterize the somatic mutational landscape across 79 metastases and 7 body fluid samples, with a range of 2 to 19 metastatic samples per patient sequenced. We analyzed the WES data with rigorous filters (STAR Methods) based on a recently described pipeline (Callari et al., 2017). To further validate the WES mutation calls (Table S2), we generated ultra-deep targeted sequencing (TS) (mean depth of 7,570−29,891×; mean coverage > 1,000× for 71%–100% of samples) for amplicons across 464 mutations (average, 46.4/case; range, 16–127) (Table S3). Matched WES and TS data were available from 79 samples, and they validated the robustness of the WES data analysis pipeline we used: sensitivity, 0.85; specificity, 0.99; accuracy, 0.91; precision, 0.99 (STAR Methods).

The WES data identified 15,430 somatic mutations across the 86 samples: 7,809 missense, 1258 truncating, 10 nonstop, 234 in-frame deletions or insertions, and 6,119 other (Table S2). The mutation burden varied between cases, with a median of 507 mutations per case (range, 113–997), and across metastases within each case, with a median mutation load of 146 per metastasis (interquartile range, 86.25). These numbers are significantly greater ($p \leq 2.2e{-}16$, Wilcoxon rank-sum test) than mutation burdens reported previously for primary breast tumors within The Cancer Genome Atlas (TCGA), with a median mutation load of 63.5 (interquartile range, 68).

We classified somatic mutations found in WES as "metastatic stem" when present in all metastases from the same case,

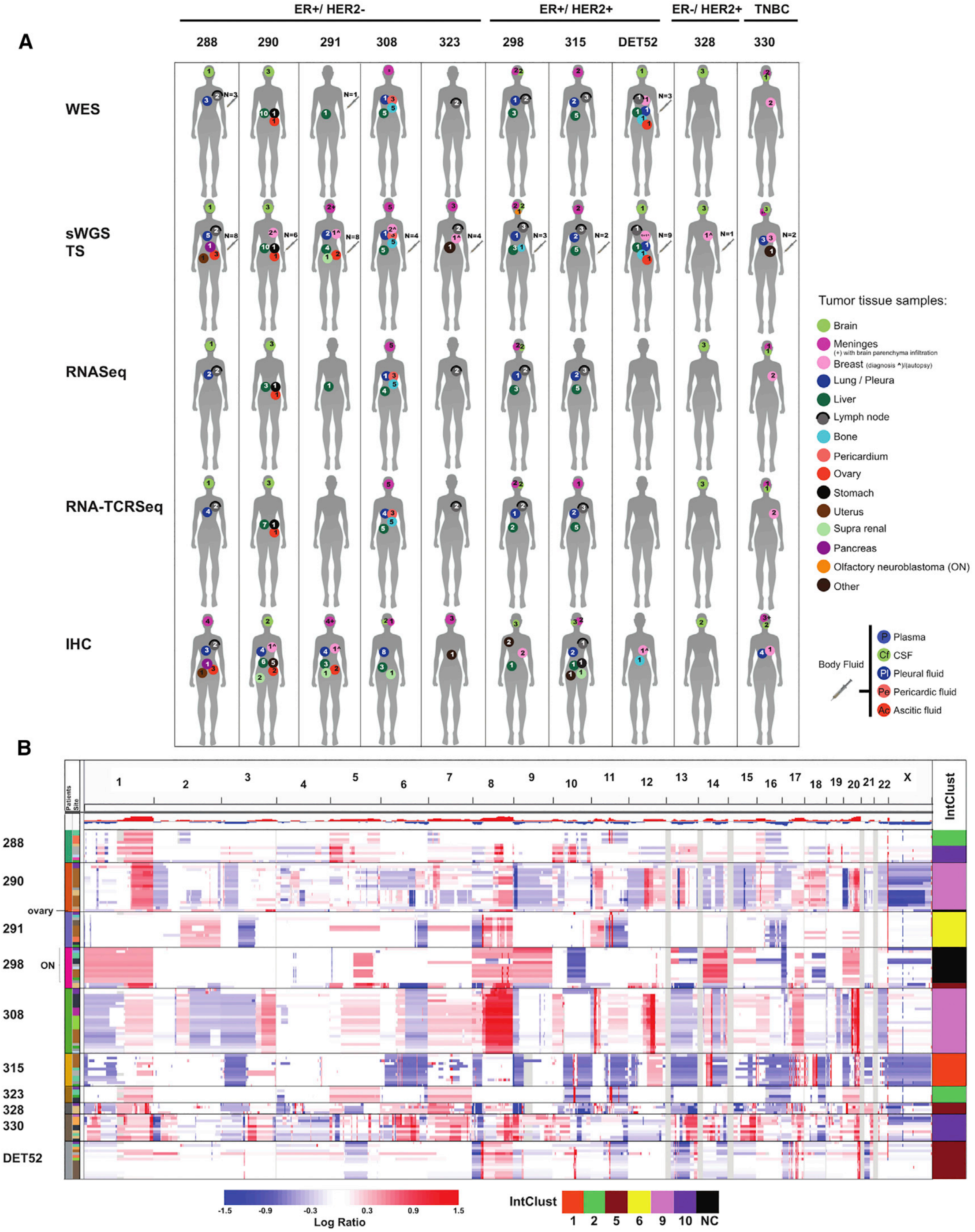

Figure 1. Molecular Profiling of 10 Lethal Metastatic Breast Cancers

(A) Silhouettes representing the 10 patients with number and type of samples profiled using each platform. Patients are grouped as indicated above the silhouettes according to ER and HER2 status. Samples profiled are labeled according to the color key panel on the right. WES, whole-exome sequencing; sWGS, shallow whole-genome sequencing; TS, targeted sequencing; RNA-seq, RNA sequencing; RNA-TCRseq, targeted TCR sequencing in RNA; IHC, immunohistochemistry; TNBC, triple-negative breast cancer.

(B) IGV plot showing the copy number aberration (CNA) landscapes across 168 metastases, with samples grouped by patient. The IntClust bar shows individual sample assignment to one of the 10 integrative clusters (Curtis et al., 2012). Copy number gains and amplifications are indicated in shades of red; copy number losses are indicated in shades of blue (see bar for corresponding Log ratio). IntClust as per color bar. NC- not classified; ON- olfactory neuroblastoma.

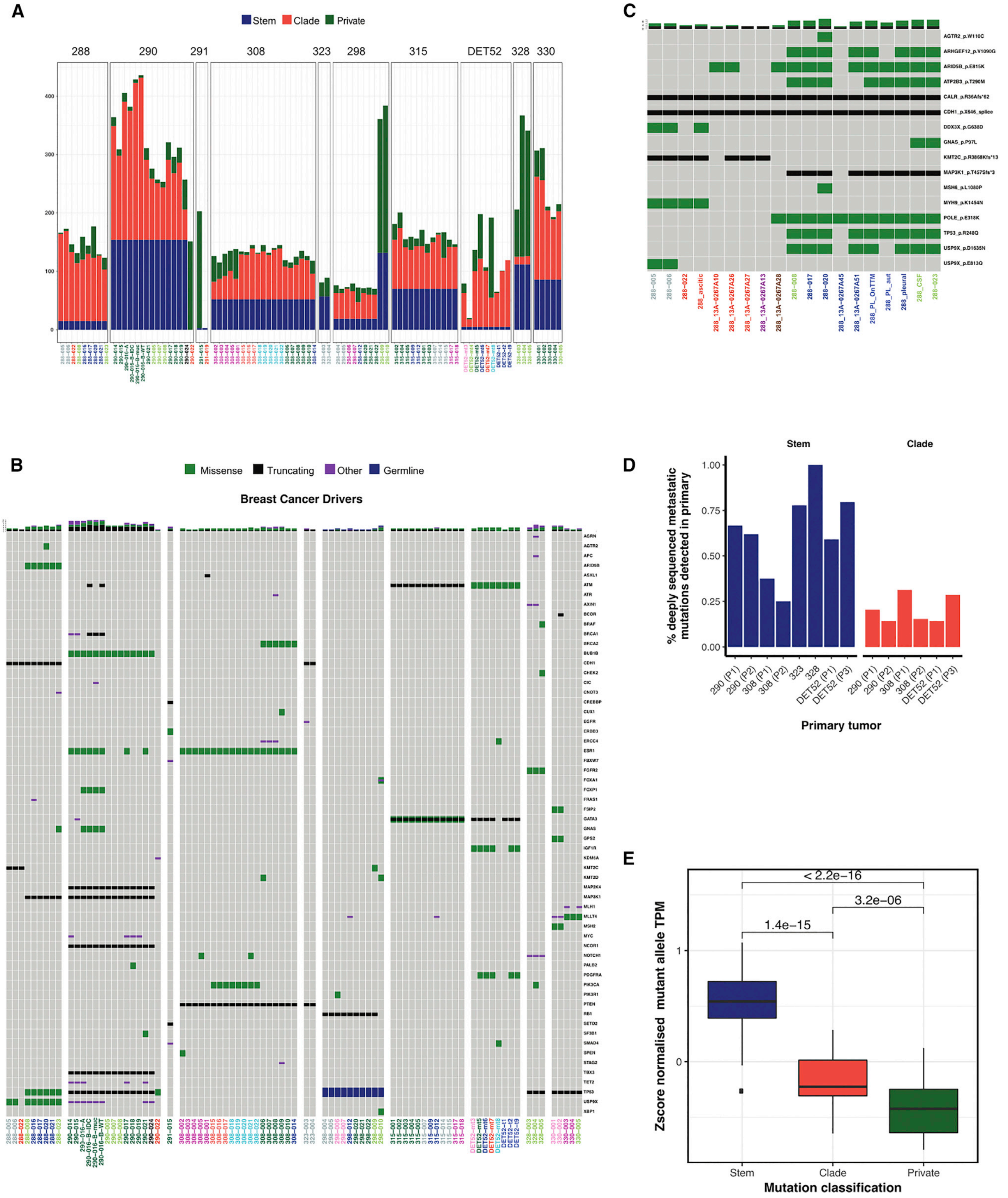

Figure 2. Mutational Landscape of 10 Lethal Metastatic Breast Cancers

(A) Mutational burden barplots across 86 metastatic samples using WES. Colors indicate mutations classified as metastatic stem, metastatic clade, and metastatic private.

(B) Oncoprint plot showing the mutations in breast cancer driver genes identified by WES across 84 metastases for the 10 patients.

(legend continued on next page)

"metastatic clade" when present in at least two but not all metastases, and "metastatic private" when present in a single metastasis (Figure 2A). This revealed that the vast majority of the mutations in individual metastasis were either stem or clade. We focused our analysis on mutation drivers (Table S4). We considered a gene a mutation driver (e.g., associated with a fitness advantage when somatically mutated) using the widely accepted framework that is based on analyses of large cancer mutation datasets (Lawrence et al., 2013; Vogelstein et al., 2013). For our analyses, we defined a list of 109 breast cancer driver genes identified from reviewing the data in three large cohorts (Lefebvre et al., 2016; Nik-Zainal et al., 2016; Pereira et al., 2016) and a list of 527 non-breast cancer drivers (non-overlapping) from the Cancer Gene Census (https://cancer.sanger.ac.uk/census). Mutations were significantly (p = 0.0002, chi-square test) more common in breast cancer driver genes (58 of 109, 53.21%) than in non-breast cancer driver genes (142 of 527, 26.94%). The total number of driver mutations per metastasis averaged 11.44 (range, 2–30) (Figure 2B; Figure S2), which is higher than the estimated number per primary tumor (Martincorena et al., 2017). Metastatic stem driver mutations were identified in all 10 cases: 2 in case 288 (*CDH1*; non-breast driver: *CALR*), 9 in case 290 (*BUB1B*, *MAP2K4*, *MAP3K1*, *NCOR1*, *TBX3*, and *TP53*; non-breast drivers: *ELL*, *MET*, and *FLT3*), 1 in case 291 (*ERBB3*), 3 in case 308 (*ESR1* and *PTEN*; non-breast driver: *NKX2-1*), 4 in case 315 (*ATM* and *GATA3,* one missense and one truncating; non-breast driver: *CDC73*), 5 in case 323 (*CDH1* and *PTEN*; non-breast drivers: *MET*, *MYCL*, and *SDC4*), 1 in case DET52 (*ATM*), 8 in case 328 (*FGFR2*, *NOTCH1*, and *TP53*; non-breast drivers: *AXIN1*, *CRTC1*, *LRIG3*, *SMARCE1*, and *WHSC1L1*), and 5 in case 330 (*TP53*; non-breast drivers: *CARD11*, *CNTRL*, *FBXO11*, and *PTPRK*). A Li-Fraumeni syndrome patient, case 298, had two known metastasized malignancies: HER2-positive breast cancer and olfactory neuroblastoma. Previously, we showed that the brain metastasis originated from the breast cancer (De Mattos-Arruda et al., 2015). The *HER2*-amplified brain metastases had 5 mutation drivers (non-breast drivers: *CACNA1D*, *DCTN1*, *FAT1*, *RAD21*, and *WHSC1*) in addition to the germline *TP53* mutation with associated somatic loss of heterozygosity. The WES data also revealed that the olfactory neuroblastoma had an *RB1* stem driver mutation. This *RB1* mutation was detected in one of the two breast cancer brain metastases (298-009), likely because of contamination by CSF cfDNA. Indeed, mutations arising from both leptomeningeal neuroblastoma and from HER2-positive brain metastases had been detected in CSF cfDNA (De Mattos-Arruda et al., 2015). In case 290, an ovarian tumor sample originally presumed to be metastatic lacked all 6 breast cancer stem mutations, including a *TP53* frameshift mutation. The sample had a different *TP53* p.Y220D missense mutation, and this prompted a pathology review, which confirmed that it was an independent primary ovarian adenocarcinoma. Most cases had also several metastatic clade driver mutations, whereas metastatic private driver mutations were uncommon.

TS data were also obtained from 40 additional samples for which only FFPE blocks were available, bringing the total number of metastatic samples, primary tumors, and liquid biopsies with TS data to 159 (average per patient, 16 samples; range, 4–25). The TS validated and extended the WES findings, and this was particularly informative in case 288, showing that the bilateral ovarian metastases shared the driver mutations with the lymph nodes and ascites (Figure 2C; SI2 in https://doi.org/10.17632/6cv77bry6m.1). In 6 cases, FFPE blocks from the original primary breast tumor were available, and TS data confirmed that all of these contained the clonal ancestors of the metastases, but a percentage of stem mutations and an even larger fraction of clade mutations were not detected (Figure 2D; SI2 in https://doi.org/10.17632/6cv77bry6m.1). This included some metastatic stem driver mutations (case 290, *BUB1B* absent in two FFPE blocks; case 308, *ESR1* and *PTEN* absent in the two FFPE blocks; DET52, *ATM* not detected in ductal carcinoma *in situ* (DCIS) or metastatic axillary lymph nodes) and most metastatic clade driver mutations (SI2 in https://doi.org/10.17632/6cv77bry6m.1).

We next asked whether the expression of the mutant allele was similar across mutations. A combined analysis of WES and RNA-seq data were possible in 8 cases (case 291 with a single metastasis with combined data and case DET52 without RNA-seq data were excluded) and revealed that the normalized expression of the mutant allele was highest in stem, lower in clade, and lowest in private mutations (Figure 2E).

In summary, metastases keep accumulating mutations, including mutations in known cancer driver genes, but an apparent hierarchy of expression (stem-clade-private) of mutant alleles suggests that, as more mutations accumulate in metastases, these are increasingly passengers (e.g., not expressed). A fraction of mutations (including drivers) shared across metastases (stem and clade) were not detectable in the available primary tumor tissue blocks, suggesting either their origin from a minor clone in the primary tumor or their acquisition in metastatic cells that had already left the breast.

Metastases Are Initiated and Maintained as Communities of Clones

A monoclonal origin of metastases should result in uniformly high variant allelic fractions (VAFs) of stem and clade mutations across all metastases in a case. Plots of allelic fractions of these mutations across individual metastases revealed a scatter of allelic fractions using WES data (Figure 3A), and this was validated using deep TS data. This observation was confirmed by calculating the cancer cell fraction (CCF), which is the VAF of each somatic mutation corrected for copy number and purity estimates, across all of the individual metastases. The probability distributions of the CCFs were then used to classify each somatic mutation as clonal or sub-clonal (McGranahan et al., 2015;

(C) Oncoprint plot showing driver mutations validated by TS (allelic fraction [AF] $\geq$ 0.1%) for case 288.
(D) Boxplot showing the percentage of stem and clade mutations identified as present by TS (AF $\geq$ 3 SD from AF in matched normal). DNA was extracted from FFPE blocks from primary surgery samples, except for case DET52, where P1 and P3 were diagnostic biopsies (breast and axillary lymph node, respectively).
(E) Boxplots of *Z* score-normalized mutant allele expression from RNA-seq data in metastatic stem, metastatic clade, and metastatic private mutations. TPM, transcripts per million. Bars indicate significance of difference (p values < 0.05 are considered statistically significant).

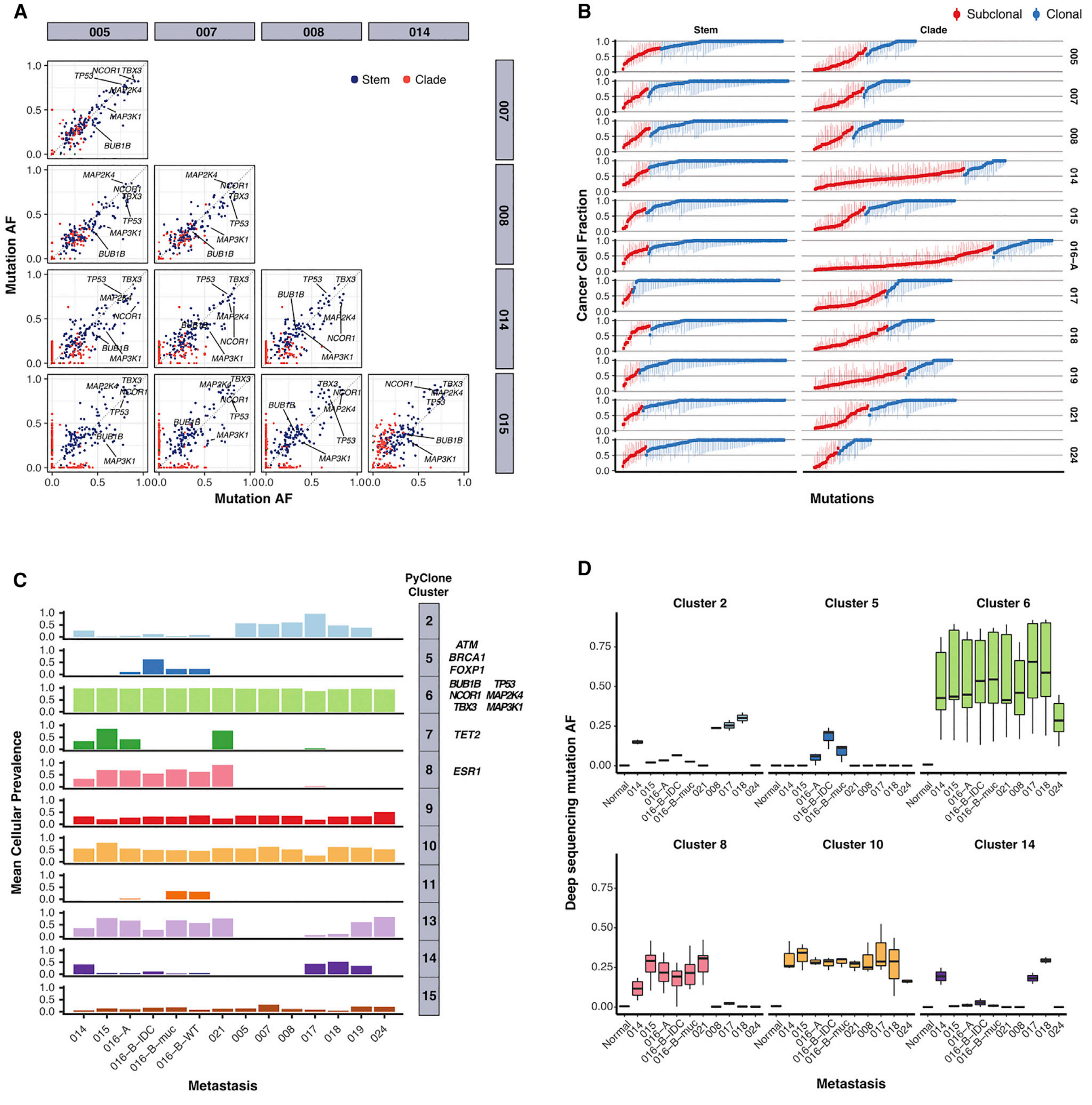

Figure 3. Breast Cancer Metastases Are Communities of Clones

(A) Pairwise comparisons of raw VAFs from WES data across 10 pairs of metastases from case 290. Metastatic stem and metastatic clade mutations are colored as indicated.

(B) Cancer cell fraction (from WES data) of metastatic stem and metastatic clade mutations across metastases in case 290. Each symbol represents a somatic mutation in an individual metastasis. Probability distributions of the CCFs were used to classify each mutation as either clonal (blue) or subclonal (red). Error bars represent the 95% confidence interval. Plots for all remainder cases are shown in SI3 in https://doi.org/10.17632/6cv77bry6m.1.

(C) Mean cellular prevalence of mutation clusters identified by PyClone from WES data across metastases in case 290. Metastatic stem (clusters 6, 9, 10, and 15) and metastatic clade (clusters 2, 5, 7, 8, 11, 13, and 14) mutation clusters are shown.

(D) Boxplots showing the distribution of mutation AFs in TS data in case 290. Amplicons representative of PyClone exome-derived mutation clusters were analyzed. Plots for all remainder cases are shown in SI3 in https://doi.org/10.17632/6cv77bry6m.1.

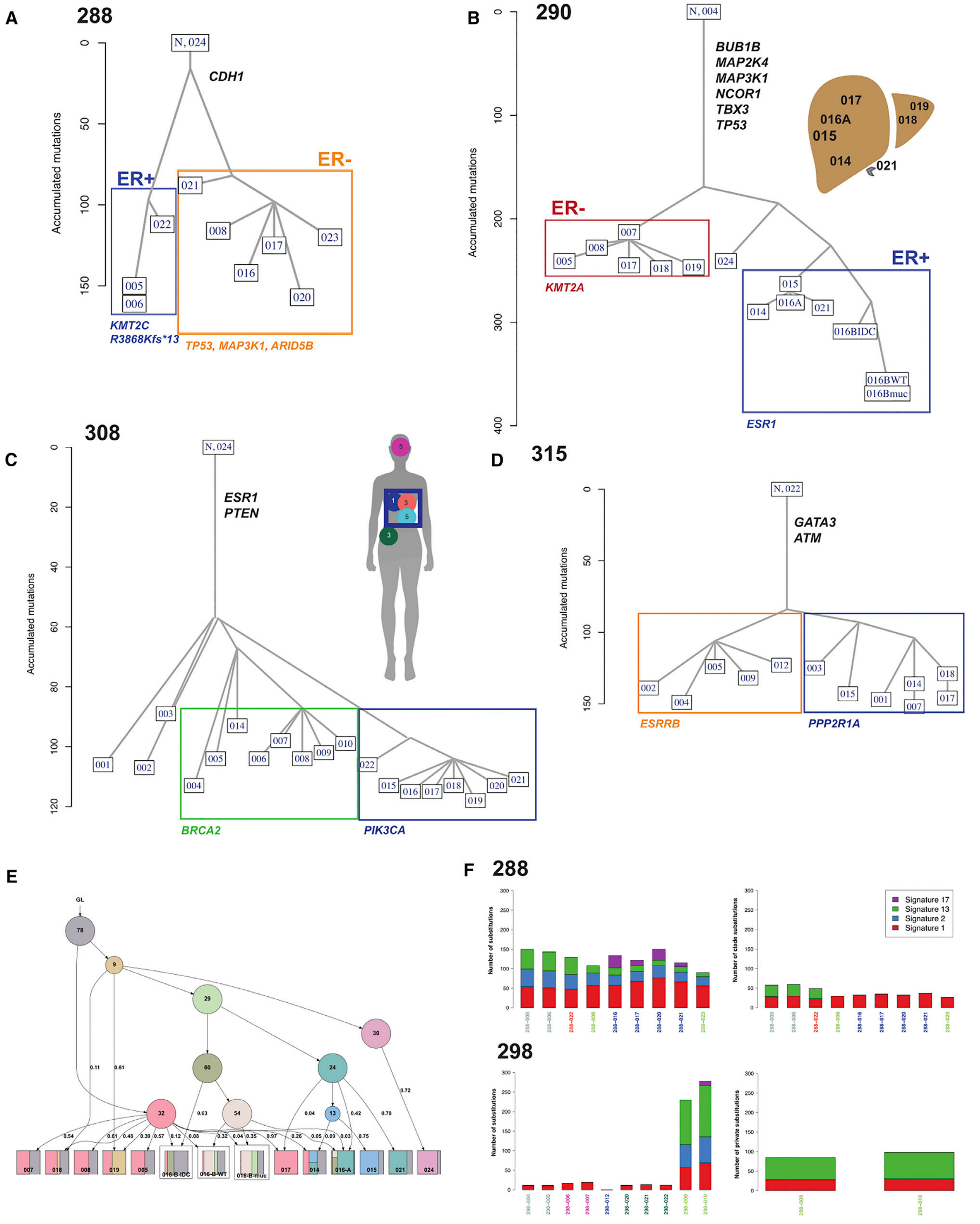

(legend on next page)

Figures 3B and S3; https://doi.org/10.17632/6cv77bry6m.1), and the results were consistent with a fraction of the metastatic stem and clade mutations being sub-clonal.

We also analyzed the WES data with PyClone (Roth et al., 2014), a Bayesian clustering method for grouping sets of somatic mutations and estimating their cellular prevalence (Figure 3C; Figure S3; SI3 in https://doi.org/10.17632/6cv77bry6m.1). In each case, a cluster constituted by metastatic stem mutations had the highest predicted cellular prevalence (mean, 0.94; range, 0.81–0.99) across metastases, as would be expected (288, cluster 2; 290, cluster 6; 308, cluster 1; 315, cluster 3; 323, cluster 2; 328, cluster 4; 330, cluster 5; Det52, cluster 1). However, there were also stem mutation clusters with lower predicted cellular prevalence, indicating that these were probably sub-clonal (290: clusters 9, 10, and 15; 308: clusters 5 and 9; 328: cluster 5; 330: cluster 6; Det52: cluster 6). Amplicons representative of the clonal clusters identified from WES were validated using TS, and this confirmed that a fraction of stem and clade mutations appeared to be sub-clonal (Figure 3D; SI3 in https://doi.org/10.17632/6cv77bry6m.1).

In summary, these analyses are not compatible with all metastatic stem and clade mutations being clonal and support the hypothesis that metastases are initiated and maintained as groups of cellular clones.

Ancestries of Multiregional Metastases Defined by Phylogenetic Analyses

We aimed to reconstruct the metastatic seeding patterns with a series of phylogenetic methods. These included OncoNEM (Ross and Markowetz, 2016) and Treeomics (Reiter et al., 2017), which employ nucleotide substitutions and short insertions and deletions (from WES or TS data) as input data; Super-Freq (Savas et al., 2016), which employs both single-nucleotide variants (SNVs) and CNAs from WES as input data; and MEDICC (Schwarz et al., 2014), which employs CNAs from sWGS and WES data as input (STAR Methods). Overall, the results were consistent across these different methods, but for clarity, we present below the results from OncoNEM (Figure 4; Figure S4A) using either WES and/or TS data (all other results are provided in SI4 in https://doi.org/10.17632/6cv77bry6m.1). The OncoNEM phylogenetic trees of metastases had branched structures in nine cases (Figure 4; Figure S4A; SI4 in https://doi.org/10.17632/6cv77bry6m.1). These trees had a limited number of main branches forming separate clades of distinct but genetically related, by common ancestry, metastatic samples. The exception was case 291, where all metastases appeared linearly related using TS data (Figure S4A; SI4 in https://doi.org/10.17632/6cv77bry6m.1), but this appearance could be an artifact resulting from high-quality WES being available from a single metastasis (291-015). In case 298, there were two breast cancer metastases analyzed, with the remainder of metastases sequenced from neuroblastoma, and metastases segregated by tumor of origin (Figure S4). Case 288 was an ER-positive lobular cancer with a classical somatic truncating *CDH1* mutation (Figure 4A). This patient had bone metastases 10 years after diagnosis, followed by contralateral axillary lymph node metastases and, later, lung and CNS metastases. The metastases detected clinically were sampled at autopsy and, in addition, metastases found at autopsy in both ovaries and the uterus, and ascites was also collected. The genomic phylogeny was clear, with two separate clades: one consisting of lymph nodes (288-005 and -006), ascites (288-022), and bilateral ovarian Krukenberg metastases (13A-0267A10, -A26, and -A27) that were all ER-positive and had a truncating *KMT2C* R3868Kfs*13 mutation and 11q13 amplification and a second clade with brain (288-008), leptomeningeal (CSF pellet, 288-023), and lung metastases (288-016, -017, -020, and -021) that were ER-negative, shared *TP53*, *MAP3K1*, and *ARID5B* mutations, and lacked *KMT2C*-R3868Kfs*13 (Figure 4A). Case 290 (Figure 4B), an ER-positive/HER2− ductal cancer, relapsed with bone metastases 2 years after diagnosis, followed by liver metastases 13 years later and death shortly after development of CNS metastases 20 years after the original diagnosis. At autopsy, several liver metastases were sampled and carefully mapped, in addition to brain and stomach metastases being collected. The ovarian sample collected at autopsy was proven genomically and upon histological review to be a separate primary adenocarcinoma (see above). The genomic phylogeny of the breast cancer metastases revealed 3 clades. One clade, defined by the presence of an *ESR1* Y537S mutation, in ER-positive metastases (290-014, -015, -016A, -016B, and -021) mapped to the right inferior lobe of the liver. A second clade of ER-negative metastases in the brain (290-005, -007, and -008) and left (290-018 and -019) and upper right (290-017) lobes of the liver was defined by the presence of a *KMT2A* mutation. A third clade was defined by the absence of both *ESR1* and *KMT2A* mutations in a stomach metastasis (290-024). Case 308 (Figure 4C), an ER-positive/HER2− ductal cancer, relapsed with bone metastases 3 years after surgery, followed 3 years later by lung metastases and then, in quick succession, CNS and liver metastases and a mediastinal mass (formed by pericardial, lung, and rib metastases) developing in the year prior to death. All 19 metastases analyzed by WES shared activating *ESR1* and truncating *PTEN* mutations. A genomic clade was defined by a *PIK3CA* mutation, shared by pericardial (308-015, -016, and -017) and bone metastases (308-018, -019, -020, -021, and -022) forming the mediastinal mass.

Figure 4. Phylogenetic Ancestries of Breast Cancer Metastases

(A–D) Phylogenetic trees from the OncoNEM algorithm. Shown are cases 288 (A), 290 (B), 308 (C), and 315 (D). Metastatic stem driver mutations and selected metastatic clade mutations are shown. Boxes identify clades. Tree branches are proportional to the number of mutations.

(E) Phylogenetic tree from the LICHeE algorithm for case 290. Circles represent the mutation clonal clusters and digits within each circle the number of mutations for each cluster. Squares represent each individual metastasis, with colored rectangles representing the cellular prevalence of the respective clonal cluster. Cross-seeding from the *KMT2A* clade to 3 metastases (014, 016-A, and 016-B-WT/muc/IDC) in the *ESR1* clade can be seen. Similar plots for all remainder cases are shown in SI4 in https://doi.org/10.17632/6cv77bry6m.1.

(F) Mutation barplots colored according to mutational signatures for cases 288 and 298. Case 288: all mutations (left panel) and clade mutations (right panel). Case 298: all mutations across samples (left panel) and private mutations of Her2+ breast cancer metastases (right panel).

Most of the remainder of the metastases (except 3 of the meningeal metastases: 308-001, -002, and -003) formed a separate clade defined by a *BRCA2* missense mutation. Case 315 (Figure 4D), an ER-positive/HER2+ ductal cancer treated with neoadjuvant chemotherapy and anti-HER2 therapy followed by surgery, relapsed 3 years after diagnosis with bone and liver metastases, received multiple lines of therapy mostly targeting HER2, and within 1 year of death (8 years after diagnosis) had progressive liver and CNS metastases. At autopsy, all 12 metastases analyzed by WES shared *ATM* and two different *GATA3* mutations. Two genomic clades were identified: one defined by a truncating mutation in *PPP2R1A*, comprised of metastases in the liver (315-001, and -003), peri-pancreatic lymph node (315-015), para-tracheal lymph nodes (315-007 and -014), and meninges (315-017 and -018) and a second clade defined by an *ESRRB* mutation in liver (315-002, -004, and -005) and lung (315-009 and -012) metastases. Case 323 (Figure S4A; SI4 in https://doi.org/10.17632/6cv77bry6m.1), an ER-positive/HER2− lobular cancer, relapsed 2 years after diagnosis with metastases in bone, pleurae, and lymph nodes. The patient died a year later with CNS involvement. Genomic phylogeny showed that the two lymph node metastases formed a single clade with *CDH1* and *PTEN* stem mutations. Case 328 (Figure S4A), an ER-negative/HER2+ ductal carcinoma, presented with breast primary and metastatic disease in the liver, and the patient died 19 months later. At autopsy, three anatomically distinct brain metastases were sampled; all shared *TP53* and *FGFR2* mutations and formed a single clade. Case 330 (Figure S4A), a triple-negative ductal cancer, was originally treated with neo-adjuvant chemotherapy, with pathological complete response at surgery. The patient relapsed after 12 months with CNS metastases, followed months later by breast metastases. Phylogenetic analysis revealed two clades: breast metastases (330-001 and -002) with *GPS2*, *MSH2*, and *FSIP2* mutations and brain (330-005) and meningeal (330-003 and -004) metastases with *AR* and *MLLT4* mutations. Case DET52 (Figure S4A), an ER-positive/HER2+ ductal carcinoma, presented with widespread metastatic disease and had a tree rooted in a single brain metastasis resected surgically 18 months prior to death (Det52, mt4), with a clade formed by ovary (Det52, mt7), liver (Det52, mt5), and lung (Det52, mt6) metastases. The OncoNEM tree constructed from TS data (SI4 in https://doi.org/10.17632/6cv77bry6m.1) showed one clade formed by diagnostic DCIS and axillary lymph node (Det52, mt2) biopsies and invasive breast cancer sampled at autopsy (Det52, mt3), and another clade formed by the distal metastases collected at autopsy (bone, mt8; liver, mt5; ovary, mt7) and the surgically resected brain metastasis (mt4).

Because metastases were grouped in clades, we asked whether cross-seeding occurred both within and between clades. TS data revealed individual instances of cross-seeding (Table S3; SI2 in https://doi.org/10.17632/6cv77bry6m.1), and therefore we used a systematic approach to quantify these. Cross-seeding is the result of a clone (or a group of clones) from one metastasis recirculating and seeding another metastasis at a different site. We used the PyClone mutation clusters (see above) as a surrogate of metastatic cellular clones and entered these into the LICHeE phylogenetic tool (Popic et al., 2015), which orders clones across samples by comparing their cellular prevalence. This revealed that cross-seeding between clades occurs in a particular pattern: a common seeder for one clade can cross-seed metastases belonging to a separate clade (Figure 4E; SI4 in https://doi.org/10.17632/6cv77bry6m.1). Cross-seeding within a clade was rare.

In summary, the genomic phylogenies of metastases are complex but consistently show that, within a patient, individual metastasis can be grouped in phylogenic clades that share a common genomic ancestry, and this ancestry is mutually exclusive with the genomic ancestries of other clades. Each clade group of metastases is therefore likely seeded by a common ancestor, but cross-seeding between metastases can happen, although it appears that this occurs to a rather limited degree and mostly between clades.

Mutational Signatures across Metastases

Mutational signatures, generated by different mutational processes, are engraved in the genomes of breast cancers (Nik-Zainal et al., 2016). To extract these signatures accurately, direct application of non-negative matrix factorization (NNMF) on our 86 WES samples would lack power; hence, data from 240 additional WES single metastatic breast samples (Lefebvre et al., 2016) were included in the analysis. To identify canonical signatures, the rank and number that could be extracted by NNMF were allowed to vary, and four were robustly seen: the APOBEC-related signatures 2 and 13 (cosine similarity of 0.98 and 0.96, respectively); signature 17, comprising mostly T > G mutations (cosine similarity of 0.95); and signature 1, associated with demethylation of cytosines (cosine similarity of 0.91) (Figure 4F; Figure S4B).

We explored whether signatures stratified across stem, clade, and private mutations. In case 288, considering all mutations revealed signatures 1, 2, and 13 in all metastases and signature 17 only in ER-negative metastases, whereas considering clade mutations revealed that signature 13 was exclusive of ER-positive metastases (Figure 4F, top panels). In case 298, signatures 2 and 13 were seen only in Her2+ breast cancer brain metastases, with enrichment of signature 13 in private mutations (Figure 4F, bottom panels). In case 328, signature 17 was seen across all 3 brain metastases but restricted to private mutations in only two (SI4 in https://doi.org/10.17632/6cv77bry6m.1), suggesting enrichment with later tumor evolution. In case DET52, a single lung metastasis (DET52-mt6) with signature 17 (Figure S4B) carried in that context the *ERBB4* mutation, believed to be the driver of resistance to lapatinib, which ultimately killed the patient.

The assignment of mutations to the four canonical signatures revealed a considerable number of "residual" mutations (SI4 in https://doi.org/10.17632/6cv77bry6m.1). Mathematically small fluctuations in either direction of these residual mutations may reflect the lack of power in WES data. However, a consistent excess of positive residuals indicates that many mutations may be due to mutational processes previously unaccounted for. To exclude the possibility that these residual mutations arise simply because of fitting to fewer canonical signatures than truly present, the dataset was fitted to the 12 breast cancer-associated signatures and all 30 canonical signatures in the Catalogue of Somatic Mutations in Cancer (COSMIC). This revealed improvement in overall fitting (as expected) at the expense of increasing negative residuals ($p = 4e{-16}$, Wilcoxon signed-rank

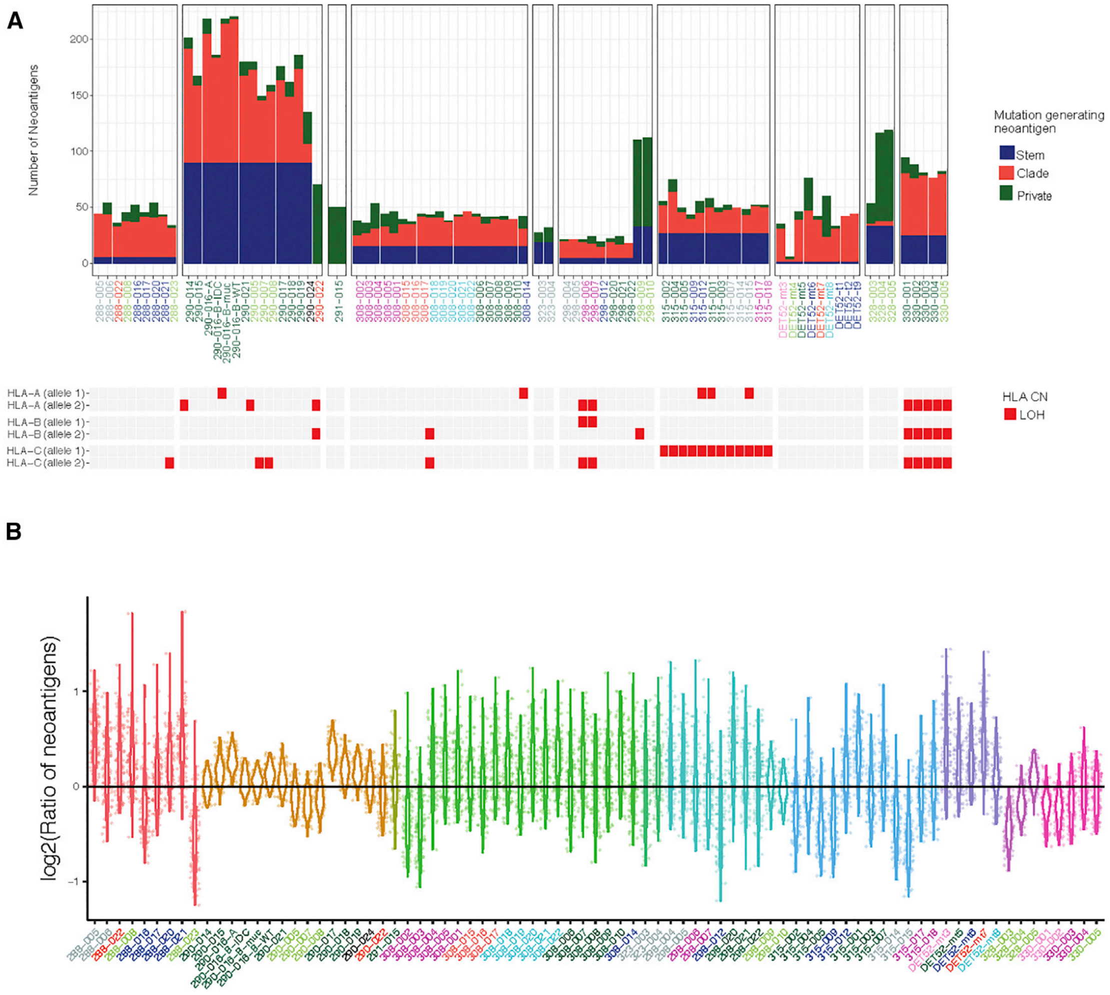

Figure 5. Neo-antigen Landscape across Breast Cancer Metastases

(A) Bar plots of the neo-antigen landscape across cases (top panel) and LOH at the HLA allelic locus across metastases (bottom panel).

(B) Violin plots of observed/expected neoantigen ratios across individual metastases. For each metastasis, 100 replicate expected mutation simulations were used, and each violin plot shows the distribution of the log2-transformed ratio. The ratio represents the relative deviation of the neo-epitope rate from expectation.

test), suggesting mis-assignment to signatures that are unlikely to be present. Next, to demonstrate that the excess of residuals was not simply due to using WES data, the same analysis was done in 640 WES primary cancers (SI4 in https://doi.org/10.17632/6cv77bry6m.1). Comparison of residuals (root-mean-square error [RMSE]) between primary and metastatic samples revealed that the fitting was much worse for metastatic cancers ($p < 2.2e-16$, Wilcoxon rank-sum test). Hence, we hypothesize that metastases with a longer history of exposure to mutational processes may carry additional signatures that are detected as the excess residuals. Indeed, cosine similarities between mutational profiles of metastatic samples and primary samples (with additional bootstrapping performed 10,000×; median $p = 3e-4$) revealed greater interpatient similarity in primary versus metastatic cancers. To further support this finding, we used the Shannon entropy index to quantify the diversity within the normalized mutational profile (considering the 96 permutations of triplet mutation context) of each sample and found greater diversity within metastatic samples compared with primary samples (Wilcoxon rank-sum test, $p = 1.6e-16$). Moreover, the evolution of diversity through the phylogenetic trees revealed a greater Shannon entropy index when all mutations were considered (Wilcoxon signed-rank test, $p = 1e-14$) versus stem mutations (SI4 in https://doi.org/10.17632/6cv77bry6m.1).

In summary, although metastases carry the same mutational signatures described in primary breast cancers, the increased residuals observed suggest that they probably carry additional mutation patterns. Remarkably mutation signatures are either shared across all metastases or across metastases within a clade, suggesting that they are scars of mutational processes operative in the metastatic founder clones.

The Predicted Neo-antigen Landscape across Metastases

Neo-antigens encoded by tumor-mutated genes result in neo-peptides that can be presented by the major histocompatibility

class I complex. These neo-peptides have the potential for binding TCRs and eliciting anti-tumor adaptive immune responses (Brown et al., 2014). We integrated WES and RNA-seq data to predict *in silico* putative neo-antigens (STAR Methods). Across the metastases, around 16% of expressed non-silent metastatic mutations yielded 1 or more predicted neo-epitopes (with IC_{50} < 500 nM), but only a small fraction (3%) of predicted neo-antigens originated from cancer drivers. Most predicted neo-antigens arise from metastatic stem (56%) and metastatic clade mutations (36%) (Figure 5A, top panel). Recently it was reported that loss of heterozygosity (LOH) at the HLA locus occurs as a mechanism of immune escape (McGranahan et al., 2017). Using the same method, we identified clonal LOH (present in every single metastasis) in cases 330 (in all three HLA class I alleles) and 315 (in HLA-C) and subclonal LOH (present in a fraction of the metastases) in cases 288, 290, 298, and 308 (Figure 5A, bottom panel). In case 330, the neo-antigens were significantly (p = 0.01, Wilcoxon rank-sum test) more commonly predicted to be presented by the lost HLA alleles. In the remainder of cases, this difference was not significant for any of the lost HLA alleles. Nevertheless, on average, 55.4% of predicted neo-antigens associated with the lost HLA allele and, hence, could not be presented directly by tumor cells.

We next asked, using a previously reported approach (Rooney et al., 2015), whether there was evidence for selected elimination of immunogenic sub-clones across individual metastases. The method relies on determining the ratio of observed-to-expected neo-epitopes, and to estimate the distribution of the number of expected neo-epitopes, we used simulated mutations that mimic the observed mutations (STAR Methods). We generated 100 datasets of simulated mutations for each sample and calculated the corresponding observed-to-expected ratios both for individual metastases and after combining all metastases from the same case or from the same organ across cases. The results (Figure 5B) showed that only one single metastasis (328-003, brain metastasis) had all 100 ratios below one (i.e., empirical p ≈ 0.01), suggesting immunoediting. We also estimated a null distribution for the mean observed-to-expected ratio using 20 of the simulated mutation datasets, generating for each one of them 100 simulated datasets and calculating the mean of the ratios (SI5 in https://doi.org/10.17632/6cv77bry6m.1). When grouping all metastases per case, none had a mean ratio lower than expected (compared with the 20 replicates) (Figure S5A), and when lumping metastases by target organ across cases, no organ site showed a trend suggesting immunoediting (Figure S5B).

In summary, in disseminated lethal breast cancer, most of the predicted neo-antigens originate from mutations shared across metastases, with only a small number being private to individual metastasis. LOH of HLA alleles was observed in many metastases (clonal in two cases), and non-synonymous mutations predicted to be neo-antigenic were frequently associated with the lost HLA allele, suggesting tumor cell immune escape. Overall, the number of predicted neo-antigens in each metastasis was only exceptionally lower than expected, suggesting that, in late metastatic breast cancer, tumor cells are already in the escape phase of the immunoediting process, where cancer cells have acquired the ability to circumvent immune recognition or destruction (Schreiber et al., 2011).

Heterogeneity of the TME across Metastases

We characterized the TME across individual metastases using a combination of computational pathology of digitally scanned H&E slides (64 frozen sections of the tissue used for RNA extraction and 102 FFPE tumor biopsies), manually scored immunohistochemistry (IHC) of a set of immune markers (n = 102), and gene expression data (RNA-seq, n = 64).

We previously reported the use of digitized H&E slides and machine learning methods to classify cells within a tumor as cancer, stromal, or lymphocytes (Ali et al., 2016b). Using this approach, we analyzed 166 frozen and paraffin-embedded metastatic and primary tissue sections (STAR Methods), and the data revealed significant heterogeneity of cell numbers, fractions, and densities across individual metastases, showing the variable spatial architecture of the TME (Figures 6A and S6A; SI6 in https://doi.org/10.17632/6cv77bry6m.1). In parallel IHC (102 FFPE sections), semiquantitative analysis by expert pathologists revealed variable numbers of CD4 and CD8 T cells per surface area (Figure 6A, bottom panel).

The patterns of immune infiltration can also be inferred using deconvolution of bulk gene expression (Hackl et al., 2016). In primary tumors, these patterns are variable across subtypes and associated with response to therapy and survival (Ali et al., 2016a). We performed these analyses across metastases from 9 cases with available RNA-seq data. The immune cytolytic activity score (Rooney et al., 2015) was highly variable across metastases (Figure 6B). We further characterized TME expression signatures using a recently reported deconvolution methodology (Charoentong et al., 2017). This tool provides normalized *Z* scores for a list of cancer immunity parameters, including 20 single factors (major histocompatibility complex [MHC] molecules, immunoinhibitors, and immunostimulators) and six cell types (STAR Methods). These *Z* scores were visualized as immunophenograms for each individual metastasis or used to generate clustered heatmaps across metastases for each case. Inspection of the immunophenograms revealed variability of the TME in metastases both between and within each of the cases (Figure 6C; SI6 in https://doi.org/10.17632/6cv77bry6m.1). This was mirrored in IHC analysis of a total of 14 TME markers, which also revealed heterogeneous TME across metastases (SI6 in https://doi.org/10.17632/6cv77bry6m.1). Unsupervised hierarchical clustering based on the Euclidean distance matrix of the *Z* scores across metastases showed that the immune parameters tended to cluster naturally into major functional groups: immunogenic or immune-suppressive (Figure 6D; Figure S6B). The clustering of the individual metastasis in each case also had interesting features. In cases 288, 290, and 330, it appeared as if the TME clustering of the metastases mirrored their genomic clades (Figure 6D; Figure S6B). The TME clustering seen could simply reflect the metastatic organ site, but in other cases, metastases to the same organ had very distinct TMEs: meningeal and bone metastases in case 308, liver metastases in case 315, and brain metastases in case 328 (Figure S6). We also examined the expression of immunomodulators (Tang et al., 2018; Thorsson et al., 2018), and this also revealed metastases largely segregated by target organ and by genomic clade (Figure 6E).

In summary, multiple orthogonal methods congruently demonstrated that the immune TME is not uniform across

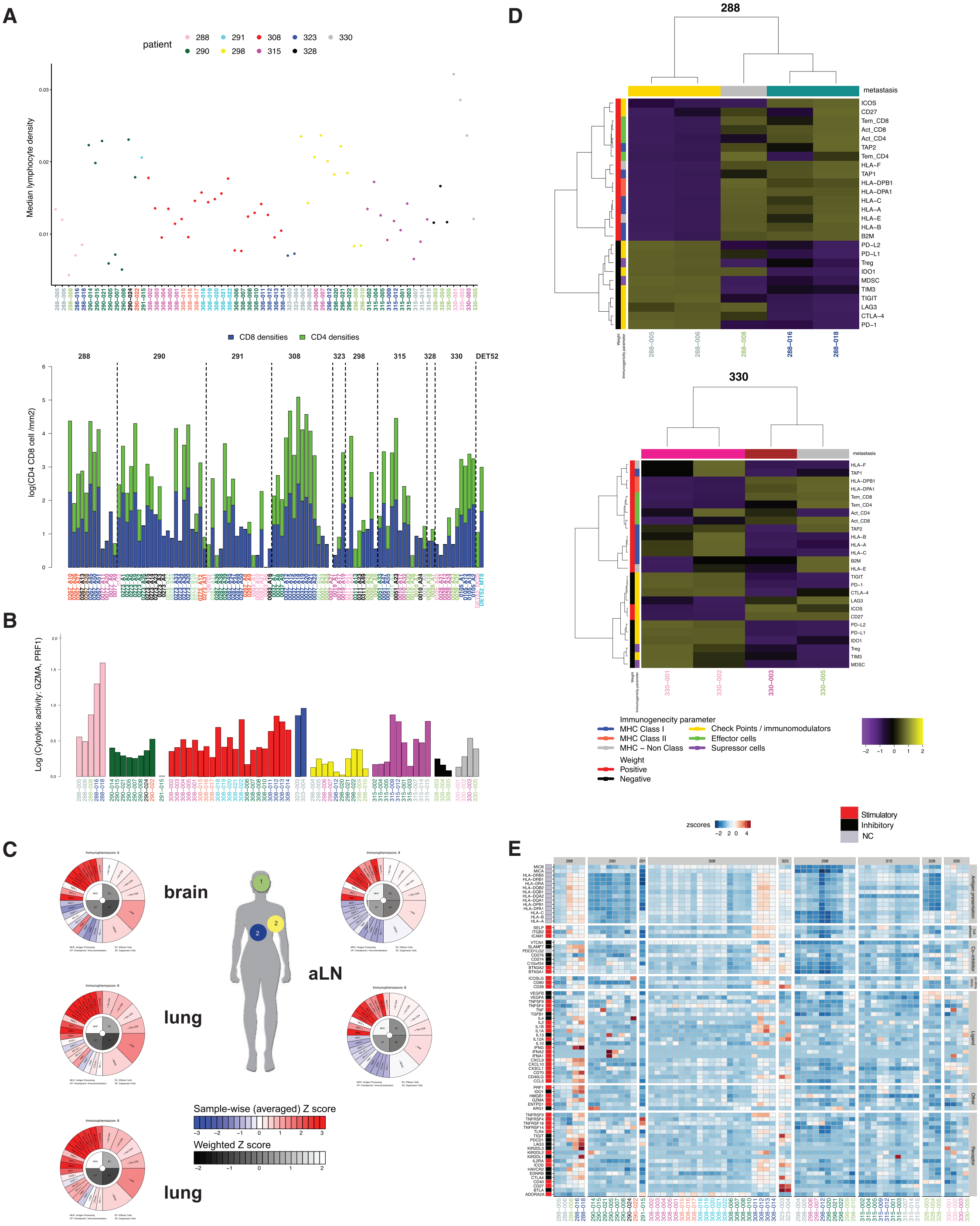

Figure 6. The Tumor Microenvironment Is Heterogeneous across Metastases

(A) Median lymphocyte density (computational pathology of digitally scanned H&E slides) (top panel) and CD4 and CD8 T number per square millimeter (IHC staining) (bottom panel).

(B) Cytolytic activity score across metastases based on transcript levels of granzyme A (GZMA) and perforin (PRF1).

(C) Immunophenograms across metastases of case 288. Each immunophenogram is color-coded in the outer part of the wheel (red, positive *Z* score; blue, negative *Z* score) for each of the parameters and gray-scaled in the inner part of the wheel, with a weighted averaged *Z* score within the respective category. *Z* scales are shown in the bars. MHC, antigen processing; CP, checkpoints/immunomodulators; EC, effector cells; SC, suppressor cells.

(D) Heatmaps depicting two-way unsupervised hierarchical clustering of immune parameters and metastases for patients 288 and 330.

(E) Gene expression of immunomodulators from RNA-seq gene expression (76 genes from Thorsson et al., 2018). *Z*-scored transformed TPMs are plotted across all 64 RNA-seq metastases from 9 patients.

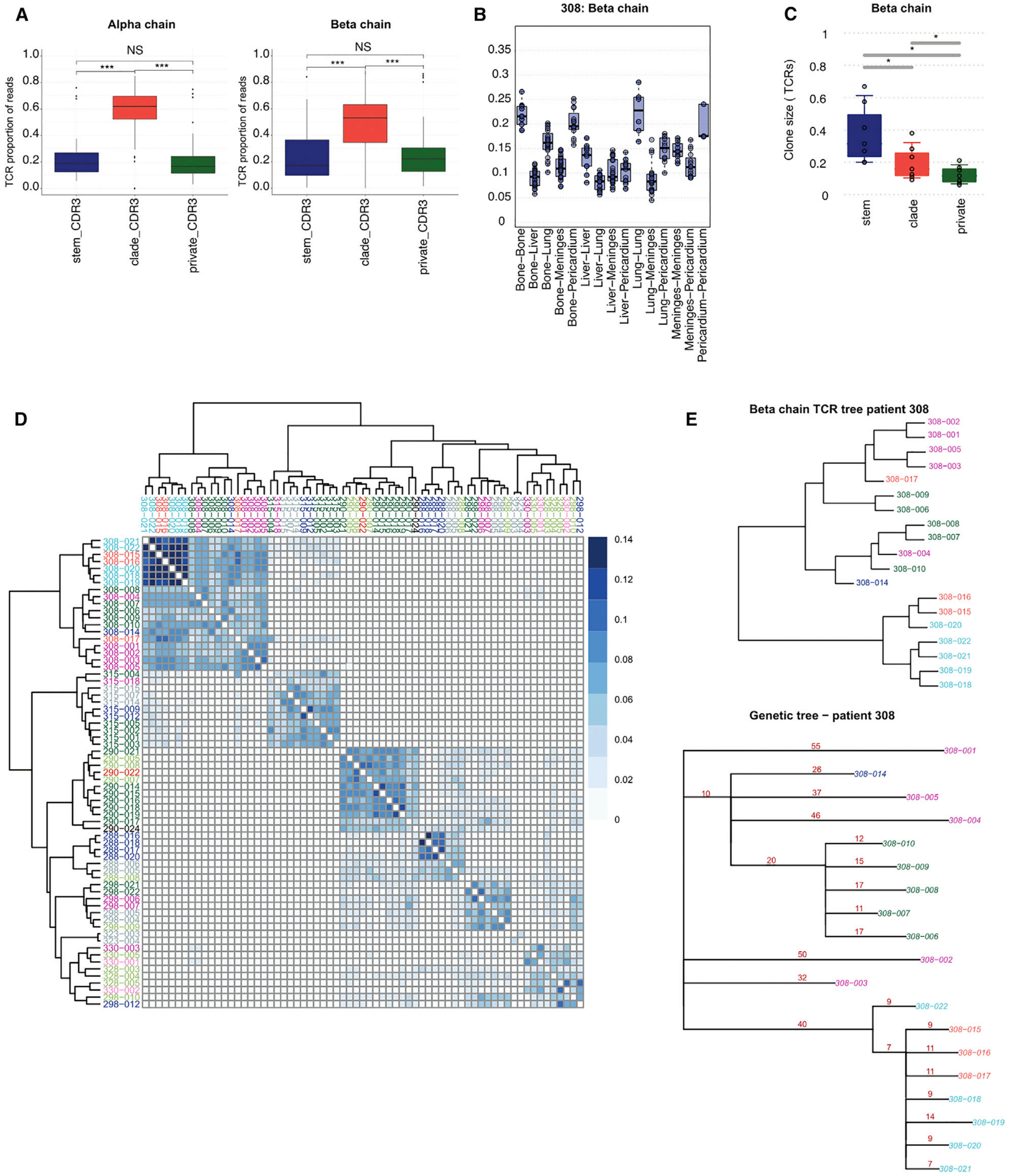

Figure 7. Analysis of the TCR Repertoire across Metastases

(A) Boxplots of proportions of TCR reads classified as metastatic stem, metastatic clade, and metastatic private. Bars indicate significance of differences (not significant [NS], $p > 0.05$; ***$p < 0.0005$).

(B) Boxplots of overlap coefficients between metastatic sites of TCR β chain nucleotide sequence repertoires. Data for case 308 is shown.

(C) Boxplots showing the TCR clone sizes according to their stem, clade, or private status. *$p < 0.05$.

(D) Clustering of TCR β chain CDR3 amino acid sequences using Jaccard distance across metastases.

(E) Jaccard tree for the TCR β chain CDR3 amino acid sequence (top panel) and the WES phylogenetic tree from OncoNEM (bottom panel) for case 308.

metastatic sites within a patient, although it can be both relatively homogeneous in metastatic clades in some cases and different across metastases to a particular organ in other cases.

The Repertoire of TCRs across Metastatic Sites

The landscape of neo-antigens is thought to determine the immunogenicity of cancers and, in particular, the anti-tumor responses mediated by T cells. We therefore characterized the repertoires of TCRs in tumor-infiltrating lymphocytes (TILs) across metastases and integrated this with the genomic data (Figure 7; Figure S7).

We sequenced the antigen binding regions of TCRs of TILs using direct amplification of the α and β TCR chains in RNA isolated from 70 metastases from 8 patients (STAR Methods). Each RNA sample was amplified and high-throughput-sequenced separately for α and β TCR chains. These sequences represented a diverse set of TCRs with a mean of 5,204 unique TCR sequences per sample (α, 5,551; β, 4,857). The α and β chain V-J gene usages were highly correlated in each metastatic sample and across all metastases, attesting to the quality of the data (Figure S7A).

Public TCRs are defined as TCRs that are shared between unrelated individuals, and these are thought to bind shared peptides; for example, of viral origin (Li et al., 2012). Therefore, we focused our analyses on non-public TCRs by first removing both the TCR β chain sequences derived from unrelated individuals in the Adaptive database (Dean et al., 2015) and the TCR α and β chain sequences shared between any of the 8 cases profiled. This filtering step enriched for TCRs that bind patient-specific antigens, including tumor neo-antigens (Figure S7B). The non-public TIL TCRs were classified in each case as stem when shared between all metastatic sites in a patient, clade when shared between some sites, or private when found in a single metastasis. Interestingly, a significant fraction of the TCR repertoire was comprised of stem (mean α/β, 21.60%/24.53%) and clade (mean α/β, 58.13%/49.67%) clonotypes (Figure 7A). These data indicate that a significant proportion of T cell clones in TILs from an individual metastasis either recirculate to other metastatic sites or that T cell clones recognizing the same neo-antigen are recruited to several metastatic sites independently. Given the evidence for T cell sharing across individual metastases, we quantified the degree of clonal sharing (STAR Methods) of CDR3 α and β clonotypes across metastases and showed significant variation between sites and patients. This is exemplified by case 308, which revealed significant differences in TCR clonal sharing between and within metastatic target organs (Figure 7B) (see SI7 in https://doi.org/10.17632/6cv77bry6m.1 for the remainder of cases). Indeed, the TCR clonal overlap coefficient was significantly higher between metastases within the same target organ than in metastases to different target organs. In addition, there was a high TCR clonal overlap coefficient between bone and pericardium metastases, which formed a mediastinal mass in case 308.

We next assessed the clonal architecture of tumor-infiltrating T cells at each site using sequence diversity measures (Bashford-Rogers et al., 2013; Madi et al., 2017). This measures the unevenness of TCR clone sizes (clonality) within each site, where each TCR clone is defined by a unique TCR sequence, and its size is defined by the frequency of that sequence within the total repertoire (STAR Methods). The T cell clonality in total mononuclear cells, CD3+ T cells, naive T cells, and central memory T cells from a healthy individual's blood sample, profiled using the same method, were very distinct from breast cancer metastases T cells; although the former exhibited only low levels of expanded T cell clones with mean largest cluster sizes of 3.93% (range, 1.03%–7.01%), most metastases showed higher levels of specific T cell clonal expansion (mean largest cluster size, 14.97) (Figure S7C). Interestingly stem and clade TCR clones were significantly more expanded than TCR clones from a single metastasis (Figure 7C; SI7 in https://doi.org/10.17632/6cv77bry6m.1), and the shared TCR clones showed significant differences in clonal representation between metastatic sites, contrasting with relatively uniform clones in normal samples (Figure S7D; SI7 in https://doi.org/10.17632/6cv77bry6m.1). The fact that T cell clones that are shared are also significantly enlarged compared with site-specific clones is suggestive of immune surveillance between metastases.

Clustering of TCR repertoires of TILs by the level of sharing of CDR3 amino acid sequences (using the Jaccard index) revealed that TCR repertoires were distinct between each metastatic breast cancer patient (Figure 7D). There was only a small degree of sharing of TCR CDR3 sequences between unrelated patients, which may occur by chance at low frequencies, and high levels of TCR CDR3 sequence sharing between metastases within each patient. Indeed, within each patient, the unsupervised CDR3 TCR clustering nearly accurately segregated the metastases by organ, and clustering was consistent with both α and β TCR chains (SI7 in https://doi.org/10.17632/6cv77bry6m.1). Using two methods for evaluating hierarchical tree structure, cophenetic correlation and Robinson-Foulds metric (STAR Methods), we showed that the TCR repertoires clustered significantly by organ ($p < 2.2e-16$) rather than by chance or by differences in TCR repertoire sampling depth.

We noted that, in 4 of the autopsy cases (288, 290, 308, and 315) where both WES and RNA TCR sequencing data were available across several metastases, the tumor OncoNEM phylogenetic trees and the TCR Jaccard phylogenetic trees had remarkably similar structures (Figure 7E; SI7 in https://doi.org/10.17632/6cv77bry6m.1). To formally test this, we used the cophenetic statistic to assess the correlation. This analysis confirmed that, in 3 of the 4 cases (288, 315, and 308), the TCR α chain trees significantly correlated with the genomic trees (cophenetic correlation of 0.460, 0.235 and 0.518; $p \leq 0.05$), and in 2 of the 4 cases (288 and 308), the TCR β chain trees also correlated with the genomic trees (cophenetic correlation of 0.38 and 0.598, $p \leq 0.08$ [borderline] and $p < 0.01$, respectively) (Figure 7E; SI7 in https://doi.org/10.17632/6cv77bry6m.1).

To further corroborate the robustness of the findings, the correlation between the RNA-seq and the TCR repertoire datasets (STAR Methods; Figure S7E) was tested. The sum of the log10 transcripts per million (TPM) values for the four CD3 complex genes (a unique marker for all T cells) and the number of TCR reads (the sum of α and β chains) were highly statistically correlated (Pearson correlation, 0.717; $p < 1.684e-10$).

In summary, these data provide a detailed view of the adaptive immune response in metastatic cancer and reveal that the TCR

repertoire of TILs is highly diverse between cases; within each case, a significant fraction of TIL TCRs are shared between metastases, suggesting immune surveillance between metastatic sites. The clonal prevalence of shared TIL TCRs in each individual metastasis can be very different; the TCR repertoire clusters metastases by target organ within each case, and tumor phylogenetic trees appear to be correlated with TIL-TCR trees across metastases within a case. This correlation suggests co-evolution between tumor diversity and T cell response across metastases.

DISCUSSION

The comprehensive molecular analysis of multi-regional metastases collected from 10 autopsies of breast cancer patients subjected to multiple lines of therapy described here details the heterogeneous landscape of genomic aberrations, TME features, and T cell adaptive immune responses in lethal cancer. The only comparably sized study (10 autopsy cases) was recently published, but it limited its analysis to DNA sequencing of the available 41 paraffin-embedded tumor samples (Brown et al., 2017).

The genomic landscapes revealed that metastatic private driver mutations are relatively uncommon and that nearly all driver CNAs and SNVs are shared across all (stem) or a subset (clade) of the metastases. The normalized expression of the mutant allele is progressively lower in metastatic clade and metastatic private mutations compared with metastatic stem mutations, suggesting that, as metastases evolve from common ancestors, they accumulate higher proportions of mutant alleles that are passengers and, therefore, have lower or suppressed expression.

In 6 cases, targeted deep sequencing of available primary samples confirmed that a fraction of tested mutations were detected at high CCFs, confirming that the metastases originated from the surgically resected tumor. However, in five of these, deep sequencing failed to identify some metastatic stem mutations and most of the metastatic clade mutations tested, including cancer driver mutations. Given the high depth and quality of the TS data, it is exceedingly unlikely that a trivial technical issue (allele dropout) could explain this result. The most likely scenarios are either that metastases originate from a minor clone not sampled in the primary tumor because of spatial heterogeneity or that metastatic stem and clade mutations could be acquired by metastatic cancer cells after these leave the breast. Indeed, in case Det52, we reported previously that metastatic stem mutations could be identified as a minor clone in the axillary lymph node, consistent with an original metastatic ancestor being present at that site (Murtaza et al., 2015). Whether that is the rule or whether other sites (e.g., the bone marrow) (Harper et al., 2016; Hosseini et al., 2016) could have a similar metastases seeder role will require studying larger numbers of cases.

The genomic phylogenetic analysis showed clearly that the multiple metastases in each case (with one exception) grouped into a small number of clades (up to three). These clades were populated by a common seeder, itself a descendant of the original metastatic ancestor, and clades were anatomically distributed to one or more target organs. In each individual metastasis within a clade, the mutations were either shared with other clade members or private, and this suggests that seeding occurs most likely in a single spreading event. Furthermore, the genomic segregation of the metastases was nearly complete, and only a limited amount of cross-seeding between clades was observed. Cross-seeding within a clade was rare. These data suggest that metastatic spread occurs as a result of a limited number of seeding events.

The classical view of the metastatic cascade has focused on seeding from single cells (Lambert et al., 2017). However, circulating tumor cell (CTC) clusters occur in the blood of patients with metastatic breast cancer, and mouse models show that CTC clusters are oligoclonal and, although rare compared with single CTCs, have 23- to 50-fold increased metastatic potential (Aceto et al., 2014). Our analysis using PyClone and CCF is consistent with metastases often being composed of communities of genomic clones, as indicated by metastatic stem mutations and metastatic clade mutations frequently being sub-clonal. Our previously published data regarding primary tumors (Shah et al., 2012) and patient-derived tumor xenografts (Bruna et al., 2016; Eirew et al., 2015) also appear to show that cancers are communities of genomic clones. The polyclonal origin of breast cancer metastases has also been reported by others (Hoadley et al., 2016) and has profound implications for the study of metastatic biology and for devising therapeutic strategies.

The mutation signatures across the metastases are a reflection of the mutational processes operative during the life history of the cancer. We identified previously reported mutational signatures in all cases and evidence of residual mutations not explained by any of the canonical mutation signatures described to date (which have been almost exclusively derived from primary cancers). Larger autopsy series and WGS data will be required to definitively establish whether metastases can accumulate novel mutation signatures reflecting both their longer natural history and the combined scars of therapies and the effects of immunoediting.

The 10 cases analyzed were patients subject to multiple lines of targeted therapy (hormone therapy and anti-Her2) and chemotherapy, to which each patient had developed resistance. The small cohort and the diversity of cancer treatments the patients received do not allow confident identification of the mechanisms of resistance. Nevertheless, case 308 had a canonical activating mutation of *ESR1* (Robinson et al., 2013; Toy et al., 2013) across all metastases, and this mutation, a likely mechanism of resistance to hormone therapy, would have been identified with a single metastatic biopsy. This contrasts with cases where two different mechanisms for hormone therapy resistance were identified: losing ER expression in some metastases and 11q13 amplification in ER-positive metastases in case 288 and losing ER expression in some metastases and an activating *ESR1* mutation in ER-positive metastases in case 290. These distinct forms of resistance in both cases correspond to their metastatic phylogenetic clades and imply that for both to be identified would require at least two metastatic biopsies.

The combined analysis of somatic cancer aberrations, TME deconvolution, predicted tumor neo-antigens, and TCR repertoire from lethal metastatic breast cancer autopsies afforded us a unique opportunity to analyze the interactions between

the malignant and TME compartments across multi-regional metastases. Most of the predicted neo-antigens arose from metastatic stem and clade mutations, and this result concurs with what has been reported in lung cancer (McGranahan et al., 2016). Immune selection, as evidenced by depletion of neo-epitopes, was scarcely seen across metastases, suggesting that in advanced breast cancer, most of the metastases are in the escape phase of immunoediting (Schreiber et al., 2011).

TME composition and its spatial architecture were also heterogeneous across metastases from individual cases. This TME diversity had no direct correlation with evidence for differential immunoediting and has also profound implications for immune checkpoint inhibitor therapy because, for example, PD1 and PDL1 expression was different across metastases. The occurrence of HLA LOH suggests that tumor cells also evolve to avoid presenting neo-antigens, and this might also contribute to immune escape.

A major determinant of the ability of the adaptive immune system to eliminate tumors is the diversity of the TCR repertoire in TILs. Although the level of sharing of TCRs between patients was minimal, an important observation was the large fraction of TCRs either shared across all the metastases (stem TCRs) or in at least 2 metastases (clade TCRs) within a case. This finding suggests that specific TCRs reacting to tumor neo-antigens, which are mostly stem and clade, are present across metastatic sites. Shared TCRs could be clonally dominant in an individual metastasis and minor clones at other metastatic sites. We could not identify any correlation between clonal inequality (measured by either Gini or Shannon index) or intra-tumor heterogeneity, mutation, or neo-antigen burden (data not shown); however, other factors, such as chemokines, may influence the migration and proliferation of T cell clones between sites.

TCR similarity was higher in metastases within a given metastatic organ. This observation led us to cluster the metastases across all patients and organs based on TCR diversity and/or similarity, revealing each patient clustered separately from all others and, within an individual patient, nearly perfect metastatic organ segregation. Very distinct TCR repertoires between patients were an expected result. However, within an individual patient, TCR repertoires in metastases to the same organ were more similar. This observation was robust and highly statistically significant, suggesting that TCR repertoires in TILs are tuned to target organs where metastases tend to share a common genomic ancestor. Indeed, genomic phylogenetic trees and TCR repertoire Jaccard trees showed remarkably similar architectures in the 4 cases where we had parallel WES and TCR sequencing data from more than 4 metastases. We tested this more formally using tools developed by ecologists, and the correlation was statistically significant in 3 of the cases. This is suggestive of co-evolution of cancer genomes and the TCR repertoires of the same metastases, providing unique patient-based evidence for the cancer immunoediting hypothesis. An alternative explanation that is not contradictory is that tissue-resident T cells, which have tissue-specific TCR repertoires, infiltrate metastases, giving rise to the observed TCR similarities. Such differences in the composition of TCR repertoires between tissues can be part of the forces that influence clonal evolution of metastatic cancer cells. These findings can have important implications for T cell-based immunotherapies. For example, peptides that are derived from neo-antigens and are used for vaccination should be tailored differently for different metastatic sites. Similarly, adoptive T cell therapies based on patient T cells or engineered receptors should also take into account differences in TCR composition and reactivity between metastatic sites. On the other hand, the presence of TCRs shared across metastases and the fact that most neo-antigens are also shared across all or subsets of metastases could be translated into either T cell-based therapies or modulation of immune checkpoints that would elicit an effective anti-tumor response across all metastases.

The extensive profiling of multi-regional metastases in lethal breast cancers resistant to several lines of therapy analyzed here has provided a unique glimpse into how metastases propagate and evolve, how drug resistance mechanisms vary, how their predicted neo-antigen landscapes look, how they shape and/or are shaped by the TME, and how the T cell adaptive immune response appears to co-evolve with the metastatic genomes. These data should motivate the research community to consider launching a lethal cancer genome project. This project, across common human cancers in a sufficiently large number of autopsy cases from patients with detailed therapy exposure histories, will produce the detailed integrated maps required for understanding resistance to therapy and escape from cancer immunoediting.

STAR★METHODS

Detailed methods are provided in the online version of this paper and include the following:

- KEY RESOURCES TABLE
- CONTACT FOR REAGENT AND RESOURCE SHARING
- EXPERIMENTAL MODEL AND SUBJECT DETAILS
  - Patients
- METHOD DETAILS
  - Sample Nomenclature
  - Nucleic acid extraction
  - WES AND sWGS ANALYSES
  - RNA-sequencing analysis
  - Neo-antigen simulations
  - T cell receptor (TCR) analysis
- QUANTIFICATION AND STATISTICAL ANALYSIS
- DATA AND SOFTWARE AVAILABILITY
  - Software
  - Data Resources

SUPPLEMENTAL INFORMATION

Supplemental Information can be found online at https://doi.org/10.1016/j.celrep.2019.04.098.

ACKNOWLEDGMENTS

This work was supported by Cancer Research UK. L.D.M.A. was partly funded by Asociación Española contra el Cáncer. The authors acknowledge Asociación Española contra el Cáncer, Cellex foundation, and the clinical team at the Breast Cancer Unit of Vall d'Hebron University Hospital/Institute of

Oncology. We are very grateful to the 10 women and their families who donated the autopsy and tumor samples with linked clinical data.

AUTHOR CONTRIBUTIONS

L.D.M.-A., S.-J.S., J.S., and C.C. conceived the experiments and wrote the manuscript. L.D.M.-A. and S.-J.S. performed the experiments and analyses with assistance from E.M.R. and F.M. (tumor phylogenies); R.B.-R., E.G., Y.T., G.V., D.R., and N.F. (TCR analysis); H.M., B.P., O.M.R., S.-F.C., T.C.-C., D.T., and M.M. (shallow WGS, whole-exome sequencing, RNA sequencing, and targeted deep sequencing data generation and analysis); S.M., T.D.A., and S.N.-Z. (mutation signatures); Y.M. and G.G. (neo-antigen observed over expected analysis); and R.M., A.A., H.R.A., W.C., A.D., N.C., E.M.-S., V.P., S.R. y C., and J.C. (clinical and pathology data).

DECLARATION OF INTERESTS

C.C. is a member of the AstraZeneca External Science Panel and has research grants from Roche, Genentech, AstraZeneca, and Servier that are administered by the University of Cambridge.

Received: January 22, 2018
Revised: December 13, 2018
Accepted: April 22, 2019
Published: May 28, 2019

REFERENCES

Aceto, N., Bardia, A., Miyamoto, D.T., Donaldson, M.C., Wittner, B.S., Spencer, J.A., Yu, M., Pely, A., Engstrom, A., Zhu, H., et al. (2014). Circulating tumor cell clusters are oligoclonal precursors of breast cancer metastasis. Cell *158*, 1110–1122.

Alexandrov, L.B., Nik-Zainal, S., Wedge, D.C., Campbell, P.J., and Stratton, M.R. (2013). Deciphering signatures of mutational processes operative in human cancer. Cell Rep. *3*, 246–259.

Ali, H.R., Rueda, O.M., Chin, S.F., Curtis, C., Dunning, M.J., Aparicio, S.A., and Caldas, C. (2014). Genome-driven integrated classification of breast cancer validated in over 7,500 samples. Genome Biol. *15*, 431.

Ali, H.R., Chlon, L., Pharoah, P.D., Markowetz, F., and Caldas, C. (2016a). Patterns of Immune Infiltration in Breast Cancer and Their Clinical Implications: A Gene-Expression-Based Retrospective Study. PLoS Med. *13*, e1002194.

Ali, H.R., Dariush, A., Provenzano, E., Bardwell, H., Abraham, J.E., Iddawela, M., Vallier, A.L., Hiller, L., Dunn, J.A., Bowden, S.J., et al. (2016b). Computational pathology of pre-treatment biopsies identifies lymphocyte density as a predictor of response to neoadjuvant chemotherapy in breast cancer. Breast Cancer Res. *18*, 21.

Altschul, S.F., Gish, W., Miller, W., Myers, E.W., and Lipman, D.J. (1990). Basic local alignment search tool. J. Mol. Biol. *215*, 403–410.

Bashford-Rogers, R.J., Palser, A.L., Huntly, B.J., Rance, R., Vassiliou, G.S., Follows, G.A., and Kellam, P. (2013). Network properties derived from deep sequencing of human B-cell receptor repertoires delineate B-cell populations. Genome Res. *23*, 1874–1884.

Bronner, I.F., Quail, M.A., Turner, D.J., and Swerdlow, H. (2014). Improved Protocols for Illumina Sequencing. Curr. Protoc. Hum. Genet. *80*, 18.2.1-42.

Brown, D., Smeets, D., Szekely, B., Larsimont, D., Szasz, A.M., Adnet, P.Y., Rothe, F., Rouas, G., Nagy, Z.I., Farago, Z., et al. (2017). Phylogenetic analysis of metastatic progression in breast cancer using somatic mutations and copy number aberrations. Nat. Commun. *8*, 14944.

Brown, S.D., Warren, R.L., Gibb, E.A., Martin, S.D., Spinelli, J.J., Nelson, B.H., and Holt, R.A. (2014). Neo-antigens predicted by tumor genome meta-analysis correlate with increased patient survival. Genome Res. *24*, 743–750.

Bruna, A., Rueda, O.M., Greenwood, W., Batra, A.S., Callari, M., Batra, R.N., Pogrebniak, K., Sandoval, J., Cassidy, J.W., Tufegdzic-Vidakovic, A., et al. (2016). A Biobank of Breast Cancer Explants with Preserved Intra-tumor Heterogeneity to Screen Anticancer Compounds. Cell *167*, 260–274.e22.

Callari, M., Sammut, S.J., De Mattos-Arruda, L., Bruna, A., Rueda, O.M., Chin, S.F., and Caldas, C. (2017). Intersect-then-combine approach: improving the performance of somatic variant calling in whole exome sequencing data using multiple aligners and callers. Genome Med. *9*, 35.

Cancer Genome Atlas, N.; Cancer Genome Atlas Network (2012). Comprehensive molecular portraits of human breast tumours. Nature *490*, 61–70.

Charoentong, P., Finotello, F., Angelova, M., Mayer, C., Efremova, M., Rieder, D., Hackl, H., and Trajanoski, Z. (2017). Pan-cancer Immunogenomic Analyses Reveal Genotype-Immunophenotype Relationships and Predictors of Response to Checkpoint Blockade. Cell Rep. *18*, 248–262.

Cibulskis, K., Lawrence, M.S., Carter, S.L., Sivachenko, A., Jaffe, D., Sougnez, C., Gabriel, S., Meyerson, M., Lander, E.S., and Getz, G. (2013). Sensitive detection of somatic point mutations in impure and heterogeneous cancer samples. Nat. Biotechnol. *31*, 213–219.

Ciriello, G., Miller, M.L., Aksoy, B.A., Senbabaoglu, Y., Schultz, N., and Sander, C. (2013). Emerging landscape of oncogenic signatures across human cancers. Nat. Genet. *45*, 1127–1133.

Curtis, C., Shah, S.P., Chin, S.F., Turashvili, G., Rueda, O.M., Dunning, M.J., Speed, D., Lynch, A.G., Samarajiwa, S., Yuan, Y., et al.; METABRIC Group (2012). The genomic and transcriptomic architecture of 2,000 breast tumours reveals novel subgroups. Nature *486*, 346–352.

De Mattos-Arruda, L., Mayor, R., Ng, C.K., Weigelt, B., Martínez-Ricarte, F., Torrejon, D., Oliveira, M., Arias, A., Raventos, C., Tang, J., et al. (2015). Cerebrospinal fluid-derived circulating tumour DNA better represents the genomic alterations of brain tumours than plasma. Nat. Commun. *6*, 8839.

Dean, J., Emerson, R.O., Vignali, M., Sherwood, A.M., Rieder, M.J., Carlson, C.S., and Robins, H.S. (2015). Annotation of pseudogenic gene segments by massively parallel sequencing of rearranged lymphocyte receptor loci. Genome Med. *7*, 123.

DePristo, M.A., Banks, E., Poplin, R., Garimella, K.V., Maguire, J.R., Hartl, C., Philippakis, A.A., del Angel, G., Rivas, M.A., Hanna, M., et al. (2011). A framework for variation discovery and genotyping using next-generation DNA sequencing data. Nat. Genet. *43*, 491–498.

Ding, L., Ellis, M.J., Li, S., Larson, D.E., Chen, K., Wallis, J.W., Harris, C.C., McLellan, M.D., Fulton, R.S., Fulton, L.L., et al. (2010). Genome remodelling in a basal-like breast cancer metastasis and xenograft. Nature *464*, 999–1005.

Dvinge, H., Git, A., Gräf, S., Salmon-Divon, M., Curtis, C., Sottoriva, A., Zhao, Y., Hirst, M., Armisen, J., Miska, E.A., et al. (2013). The shaping and functional consequences of the microRNA landscape in breast cancer. Nature *497*, 378–382.

Eirew, P., Steif, A., Khattra, J., Ha, G., Yap, D., Farahani, H., Gelmon, K., Chia, S., Mar, C., Wan, A., et al. (2015). Dynamics of genomic clones in breast cancer patient xenografts at single-cell resolution. Nature *518*, 422–426.

Flensburg, C., Sargeant, T., Oshlack, A., and Majewski, I. (2018). SuperFreq: Integrated mutation detection and clonal tracking in cancer. bioRxiv. https://doi.org/10.1101/380097.

Galili, T. (2015). dendextend: an R package for visualizing, adjusting and comparing trees of hierarchical clustering. Bioinformatics *31*, 3718–3720.

Gaujoux, R., and Seoighe, C. (2010). A flexible R package for nonnegative matrix factorization. BMC Bioinformatics *11*, 367.

Hackl, H., Charoentong, P., Finotello, F., and Trajanoski, Z. (2016). Computational genomics tools for dissecting tumour-immune cell interactions. Nat. Rev. Genet. *17*, 441–458.

Harper, K.L., Sosa, M.S., Entenberg, D., Hosseini, H., Cheung, J.F., Nobre, R., Avivar-Valderas, A., Nagi, C., Girnius, N., Davis, R.J., et al. (2016). Mechanism of early dissemination and metastasis in Her2+ mammary cancer. Nature *540*, 588–592.

Hoadley, K.A., Siegel, M.B., Kanchi, K.L., Miller, C.A., Ding, L., Zhao, W., He, X., Parker, J.S., Wendl, M.C., Fulton, R.S., et al. (2016). Tumor Evolution in Two Patients with Basal-like Breast Cancer: A Retrospective Genomics Study of Multiple Metastases. PLoS Med. *13*, e1002174.

Hosseini, H., Obradović, M.M.S., Hoffmann, M., Harper, K.L., Sosa, M.S., Werner-Klein, M., Nanduri, L.K., Werno, C., Ehrl, C., Maneck, M., et al.

(2016). Early dissemination seeds metastasis in breast cancer. Nature *540*, 552–558.

Huber, W., Carey, V.J., Gentleman, R., Anders, S., Carlson, M., Carvalho, B.S., Bravo, H.C., Davis, S., Gatto, L., Girke, T., et al. (2015). Orchestrating high-throughput genomic analysis with Bioconductor. Nat. Methods *12*, 115–121.

Hundal, J., Carreno, B.M., Petti, A.A., Linette, G.P., Griffith, O.L., Mardis, E.R., and Griffith, M. (2016). pVAC-Seq: A genome-guided in silico approach to identifying tumor neoantigens. Genome Med. *8*, 11.

Juric, D., Castel, P., Griffith, M., Griffith, O.L., Won, H.H., Ellis, H., Ebbesen, S.H., Ainscough, B.J., Ramu, A., Iyer, G., et al. (2015). Convergent loss of PTEN leads to clinical resistance to a PI(3)Kα inhibitor. Nature *518*, 240–244.

Koboldt, D.C., Zhang, Q., Larson, D.E., Shen, D., McLellan, M.D., Lin, L., Miller, C.A., Mardis, E.R., Ding, L., and Wilson, R.K. (2012). VarScan 2: somatic mutation and copy number alteration discovery in cancer by exome sequencing. Genome Res. *22*, 568–576.

Koressaar, T., and Remm, M. (2007). Enhancements and modifications of primer design program Primer3. Bioinformatics *23*, 1289–1291.

Lambert, A.W., Pattabiraman, D.R., and Weinberg, R.A. (2017). Emerging Biological Principles of Metastasis. Cell *168*, 670–691.

Lawrence, M.S., Stojanov, P., Polak, P., Kryukov, G.V., Cibulskis, K., Sivachenko, A., Carter, S.L., Stewart, C., Mermel, C.H., Roberts, S.A., et al. (2013). Mutational heterogeneity in cancer and the search for new cancer-associated genes. Nature *499*, 214–218.

Lawrence, M.S., Stojanov, P., Mermel, C.H., Robinson, J.T., Garraway, L.A., Golub, T.R., Meyerson, M., Gabriel, S.B., Lander, E.S., and Getz, G. (2014). Discovery and saturation analysis of cancer genes across 21 tumour types. Nature *505*, 495–501.

Lefebvre, C., Bachelot, T., Filleron, T., Pedrero, M., Campone, M., Soria, J.C., Massard, C., Lévy, C., Arnedos, M., Lacroix-Triki, M., et al. (2016). Mutational Profile of Metastatic Breast Cancers: A Retrospective Analysis. PLoS Med. *13*, e1002201.

Lefranc, M.P. (2011). IMGT unique numbering for the variable (V), constant (C), and groove (G) domains of IG, TR, MH, IgSF, and MhSF. Cold Spring Harb. Protoc. *2011*, 633–642.

Li, H., and Durbin, R. (2009). Fast and accurate short read alignment with Burrows-Wheeler transform. Bioinformatics *25*, 1754–1760.

Li, H., Handsaker, B., Wysoker, A., Fennell, T., Ruan, J., Homer, N., Marth, G., Abecasis, G., and Durbin, R.; 1000 Genome Project Data Processing Subgroup (2009). The Sequence Alignment/Map format and SAMtools. Bioinformatics *25*, 2078–2079.

Li, H., Ye, C., Ji, G., and Han, J. (2012). Determinants of public T cell responses. Cell Res. *22*, 33–42.

Madi, A., Poran, A., Shifrut, E., Reich-Zeliger, S., Greenstein, E., Zaretsky, I., Arnon, T., Laethem, F.V., Singer, A., Lu, J., et al. (2017). T cell receptor repertoires of mice and humans are clustered in similarity networks around conserved public CDR3 sequences. eLife *6*, e22057.

Martincorena, I., Raine, K.M., Gerstung, M., Dawson, K.J., Haase, K., Van Loo, P., Davies, H., Stratton, M.R., and Campbell, P.J. (2017). Universal Patterns of Selection in Cancer and Somatic Tissues. Cell *171*, 1029–1041.e21.

McGranahan, N., Favero, F., de Bruin, E.C., Birkbak, N.J., Szallasi, Z., and Swanton, C. (2015). Clonal status of actionable driver events and the timing of mutational processes in cancer evolution. Sci. Transl. Med. *7*, 283ra54.

McGranahan, N., Furness, A.J., Rosenthal, R., Ramskov, S., Lyngaa, R., Saini, S.K., Jamal-Hanjani, M., Wilson, G.A., Birkbak, N.J., Hiley, C.T., et al. (2016). Clonal neoantigens elicit T cell immunoreactivity and sensitivity to immune checkpoint blockade. Science *351*, 1463–1469.

McGranahan, N., Rosenthal, R., Hiley, C.T., Rowan, A.J., Watkins, T.B.K., Wilson, G.A., Birkbak, N.J., Veeriah, S., Van Loo, P., Herrero, J., et al. (2017). Allele-Specific HLA Loss and Immune Escape in Lung Cancer Evolution. Cell *171*, 1259–1271.e11.

McLaren, W., Gil, L., Hunt, S.E., Riat, H.S., Ritchie, G.R., Thormann, A., Flicek, P., and Cunningham, F. (2016). The Ensembl Variant Effect Predictor. Genome Biol. *17*, 122.

Mermel, C.H., Schumacher, S.E., Hill, B., Meyerson, M.L., Beroukhim, R., and Getz, G. (2011). GISTIC2.0 facilitates sensitive and confident localization of the targets of focal somatic copy-number alteration in human cancers. Genome Biol. *12*, R41.

Munson, D.J., Egelston, C.A., Chiotti, K.E., Parra, Z.E., Bruno, T.C., Moore, B.L., Nakano, T.A., Simons, D.L., Jimenez, G., Yim, J.H., et al. (2016). Identification of shared TCR sequences from T cells in human breast cancer using emulsion RT-PCR. Proc. Natl. Acad. Sci. USA *113*, 8272–8277.

Murtaza, M., Dawson, S.J., Pogrebniak, K., Rueda, O.M., Provenzano, E., Grant, J., Chin, S.F., Tsui, D.W., Marass, F., Gale, D., et al. (2015). Multifocal clonal evolution characterized using circulating tumour DNA in a case of metastatic breast cancer. Nat. Commun. *6*, 8760.

Nik-Zainal, S., Alexandrov, L.B., Wedge, D.C., Van Loo, P., Greenman, C.D., Raine, K., Jones, D., Hinton, J., Marshall, J., Stebbings, L.A., et al.; Breast Cancer Working Group of the International Cancer Genome Consortium (2012a). Mutational processes molding the genomes of 21 breast cancers. Cell *149*, 979–993.

Nik-Zainal, S., Van Loo, P., Wedge, D.C., Alexandrov, L.B., Greenman, C.D., Lau, K.W., Raine, K., Jones, D., Marshall, J., Ramakrishna, M., et al.; Breast Cancer Working Group of the International Cancer Genome Consortium (2012b). The life history of 21 breast cancers. Cell *149*, 994–1007.

Nik-Zainal, S., Davies, H., Staaf, J., Ramakrishna, M., Glodzik, D., Zou, X., Martincorena, I., Alexandrov, L.B., Martin, S., Wedge, D.C., et al. (2016). Landscape of somatic mutations in 560 breast cancer whole-genome sequences. Nature *534*, 47–54.

Olshen, A.B., Venkatraman, E.S., Lucito, R., and Wigler, M. (2004). Circular binary segmentation for the analysis of array-based DNA copy number data. Biostatistics *5*, 557–572.

Paradis, E., Claude, J., and Strimmer, K. (2004). APE: Analyses of Phylogenetics and Evolution in R language. Bioinformatics *20*, 289–290.

Park, J.H., Jang, M., Tarhan, Y.E., Katagiri, T., Sasa, M., Miyoshi, Y., Kalari, K.R., Suman, V.J., Weinshilboum, R., Wang, L., et al. (2016). Clonal expansion of antitumor T cells in breast cancer correlates with response to neoadjuvant chemotherapy. Int. J. Oncol. *49*, 471–478.

Parker, J.S., Mullins, M., Cheang, M.C., Leung, S., Voduc, D., Vickery, T., Davies, S., Fauron, C., He, X., Hu, Z., et al. (2009). Supervised risk predictor of breast cancer based on intrinsic subtypes. J. Clin. Oncol. *27*, 1160–1167.

Pereira, B., Chin, S.F., Rueda, O.M., Vollan, H.K., Provenzano, E., Bardwell, H.A., Pugh, M., Jones, L., Russell, R., Sammut, S.J., et al. (2016). The somatic mutation profiles of 2,433 breast cancers refines their genomic and transcriptomic landscapes. Nat. Commun. *7*, 11479.

Popic, V., Salari, R., Hajirasouliha, I., Kashef-Haghighi, D., West, R.B., and Batzoglou, S. (2015). Fast and scalable inference of multi-sample cancer lineages. Genome Biol. *16*, 91.

Rainer, J. (2016). EnsDb.Hsapiens.v75: Ensembl based annotation package, R package version 2.1.0.

Reiter, J.G., Makohon-Moore, A.P., Gerold, J.M., Bozic, I., Chatterjee, K., Iacobuzio-Donahue, C.A., Vogelstein, B., and Nowak, M.A. (2017). Reconstructing metastatic seeding patterns of human cancers. Nat. Commun. *8*, 14114.

Robinson, J.T., Thorvaldsdóttir, H., Winckler, W., Guttman, M., Lander, E.S., Getz, G., and Mesirov, J.P. (2011). Integrative genomics viewer. Nat. Biotechnol. *29*, 24–26.

Robinson, D.R., Wu, Y.M., Vats, P., Su, F., Lonigro, R.J., Cao, X., Kalyana-Sundaram, S., Wang, R., Ning, Y., Hodges, L., et al. (2013). Activating ESR1 mutations in hormone-resistant metastatic breast cancer. Nat. Genet. *45*, 1446–1451.

Rooney, M.S., Shukla, S.A., Wu, C.J., Getz, G., and Hacohen, N. (2015). Molecular and genetic properties of tumors associated with local immune cytolytic activity. Cell *160*, 48–61.

Rosenthal, R., McGranahan, N., Herrero, J., Taylor, B.S., and Swanton, C. (2016). DeconstructSigs: delineating mutational processes in single tumors distinguishes DNA repair deficiencies and patterns of carcinoma evolution. Genome Biol. *17*, 31.

Ross, E.M., and Markowetz, F. (2016). OncoNEM: inferring tumor evolution from single-cell sequencing data. Genome Biol. *17*, 69.

Ross, E.M., Haase, K., Van Loo, P., and Markowetz, F. (2017). Allele-specific multi-sample copy number segmentation. bioRxiv. https://doi.org/10.1101/166017.

Roth, A., Khattra, J., Yap, D., Wan, A., Laks, E., Biele, J., Ha, G., Aparicio, S., Bouchard-Côté, A., and Shah, S.P. (2014). PyClone: statistical inference of clonal population structure in cancer. Nat. Methods *11*, 396–398.

Saunders, C.T., Wong, W.S., Swamy, S., Becq, J., Murray, L.J., and Cheetham, R.K. (2012). Strelka: accurate somatic small-variant calling from sequenced tumor-normal sample pairs. Bioinformatics *28*, 1811–1817.

Savas, P., Teo, Z.L., Lefevre, C., Flensburg, C., Caramia, F., Alsop, K., Mansour, M., Francis, P.A., Thorne, H.A., Silva, M.J., et al. (2016). The Subclonal Architecture of Metastatic Breast Cancer: Results from a Prospective Community-Based Rapid Autopsy Program "CASCADE". PLoS Med. *13*, e1002204.

Scheinin, I., Sie, D., Bengtsson, H., van de Wiel, M.A., Olshen, A.B., van Thuijl, H.F., van Essen, H.F., Eijk, P.P., Rustenburg, F., Meijer, G.A., et al. (2014). DNA copy number analysis of fresh and formalin-fixed specimens by shallow whole-genome sequencing with identification and exclusion of problematic regions in the genome assembly. Genome Res. *24*, 2022–2032.

Schreiber, R.D., Old, L.J., and Smyth, M.J. (2011). Cancer immunoediting: integrating immunity's roles in cancer suppression and promotion. Science *331*, 1565–1570.

Schwarz, R.F., Trinh, A., Sipos, B., Brenton, J.D., Goldman, N., and Markowetz, F. (2014). Phylogenetic quantification of intra-tumour heterogeneity. PLoS Comput. Biol. *10*, e1003535.

Shah, S.P., Morin, R.D., Khattra, J., Prentice, L., Pugh, T., Burleigh, A., Delaney, A., Gelmon, K., Guliany, R., Senz, J., et al. (2009). Mutational evolution in a lobular breast tumour profiled at single nucleotide resolution. Nature *461*, 809–813.

Shah, S.P., Roth, A., Goya, R., Oloumi, A., Ha, G., Zhao, Y., Turashvili, G., Ding, J., Tse, K., Haffari, G., et al. (2012). The clonal and mutational evolution spectrum of primary triple-negative breast cancers. Nature *486*, 395–399.

Shen, Z., Qu, W., Wang, W., Lu, Y., Wu, Y., Li, Z., Hang, X., Wang, X., Zhao, D., and Zhang, C. (2010). MPprimer: a program for reliable multiplex PCR primer design. BMC Bioinformatics *11*, 143.

Shukla, S.A., Rooney, M.S., Rajasagi, M., Tiao, G., Dixon, P.M., Lawrence, M.S., Stevens, J., Lane, W.J., Dellagatta, J.L., Steelman, S., et al. (2015). Comprehensive analysis of cancer-associated somatic mutations in class I HLA genes. Nat. Biotechnol. *33*, 1152–1158.

Soetaert, K., Van den Meersche, K., and van Oevelen, D. (2009). limSolve: Solving Linear Inverse Models, R-package version 1.5.1.

Stamatakis, A. (2014). RAxML version 8: a tool for phylogenetic analysis and post-analysis of large phylogenies. Bioinformatics *30*, 1312–1313.

Tang, J., Shalabi, A., and Hubbard-Lucey, V.M. (2018). Comprehensive analysis of the clinical immuno-oncology landscape. Ann. Oncol. *29*, 84–91.

Thorsson, V., Gibbs, D.L., Brown, S.D., Wolf, D., Bortone, D.S., Ou Yang, T.H., Porta-Pardo, E., Gao, G.F., Plaisier, C.L., Eddy, J.A., et al. (2018). The Immune Landscape of Cancer. Immunity *48*, 812–830.e14.

Toy, W., Shen, Y., Won, H., Green, B., Sakr, R.A., Will, M., Li, Z., Gala, K., Fanning, S., King, T.A., et al. (2013). ESR1 ligand-binding domain mutations in hormone-resistant breast cancer. Nat. Genet. *45*, 1439–1445.

Untergasser, A., Cutcutache, I., Koressaar, T., Ye, J., Faircloth, B.C., Remm, M., and Rozen, S.G. (2012). Primer3–new capabilities and interfaces. Nucleic Acids Res. *40*, e115.

Van Loo, P., Nordgard, S.H., Lingjærde, O.C., Russnes, H.G., Rye, I.H., Sun, W., Weigman, V.J., Marynen, P., Zetterberg, A., Naume, B., et al. (2010). Allele-specific copy number analysis of tumors. Proc. Natl. Acad. Sci. USA *107*, 16910–16915.

Vogelstein, B., Papadopoulos, N., Velculescu, V.E., Zhou, S., Diaz, L.A., Jr., and Kinzler, K.W. (2013). Cancer genome landscapes. Science *339*, 1546–1558.

Wang, K., Li, M., and Hakonarson, H. (2010). ANNOVAR: functional annotation of genetic variants from high-throughput sequencing data. Nucleic Acids Res. *38*, e164.

Wang, T., Wang, C., Wu, J., He, C., Zhang, W., Liu, J., Zhang, R., Lv, Y., Li, Y., Zeng, X., et al. (2017). The Different T-cell Receptor Repertoires in Breast Cancer Tumors, Draining Lymph Nodes, and Adjacent Tissues. Cancer Immunol. Res. *5*, 148–156.

Watson, S.J., Welkers, M.R., Depledge, D.P., Coulter, E., Breuer, J.M., de Jong, M.D., and Kellam, P. (2013). Viral population analysis and minority-variant detection using short read next-generation sequencing. Philos. Trans. R. Soc. Lond. B Biol. Sci. *368*, 20120205.

Wolff, A.C., Hammond, M.E., Hicks, D.G., Dowsett, M., McShane, L.M., Allison, K.H., Allred, D.C., Bartlett, J.M., Bilous, M., Fitzgibbons, P., et al.; American Society of Clinical Oncology; College of American Pathologists (2013). Recommendations for human epidermal growth factor receptor 2 testing in breast cancer: American Society of Clinical Oncology/College of American Pathologists clinical practice guideline update. J. Clin. Oncol. *31*, 3997–4013.

Yates, L.R., Gerstung, M., Knappskog, S., Desmedt, C., Gundem, G., Van Loo, P., Aas, T., Alexandrov, L.B., Larsimont, D., Davies, H., et al. (2015). Subclonal diversification of primary breast cancer revealed by multiregion sequencing. Nat. Med. *21*, 751–759.

Yates, L.R., Knappskog, S., Wedge, D., Farmery, J.H.R., Gonzalez, S., Martincorena, I., Alexandrov, L.B., Van Loo, P., Haugland, H.K., Lilleng, P.K., et al. (2017). Genomic Evolution of Breast Cancer Metastasis and Relapse. Cancer Cell *32*, 169–184.e167.

STAR★METHODS

KEY RESOURCES TABLE

REAGENT or RESOURCE	SOURCE	IDENTIFIER
Antibodies		
CD68 Antibody	Novocastra	Cat# NCL-CD68; RRID: AB_563623
CD3 (Clone SP7)	Thermo Scientific	Cat# RM-9107-S; RRID: AB_149922
CD19 Antibody	Abcam	Cat# ab134114
Anti FOXP3 Antibody	Abcam	Cat# ab20034; RRID: AB_445284
CD8 Monoclonal Antibody	Thermo Scientific	Cat# RM-9116-S; RRID: AB_149960
Anti-IL3RA	Atlas	Cat# HPA003539; RRID: AB_1078438
Anti-IDO1 Antibody	Atlas	Cat# HPA027772; RRID: AB_1846222
CD4 Antibody	Novocastra	Cat# CD4-368-L-CE
CD56 Antibody	Novocastra	Cat# CD56-504-L-CE
CD1A Antibody	Novocastra	Cat# CD1A-235-L-CE
Mast Cell Tryptase Antibody	DAKO	Cat# M7052; RRID: AB_2206478
CD45RO Antibody	DAKO	Cat# M0742; RRID: AB_2237910
CD38 Antibody	Novocastra	Cat# NCL-L-CD38-290; RRID: AB_563555
PDL1 Antibody	Cell Signaling Technologies	Cat# 13684; RRID: AB_2687655
ER	Novocastra	Cat# NCL-ER-6F11/2; RRID: AB_876939
PR	Dako	Cat# M3569; RRID: AB_2532076
HER2	Abbott Diagnostics	Cat# 06N46-035
Raindance Source Chips	Raindance Techologies (BioRad)	Cat# 20-04410
TaqMan Genotyping Master Mix	Thermo Fisher	Cat# 4371353
SPRIselect Reagent	Beckman Coulter	Cat# B23318
Critical Commercial Assays		
DNeasy Blood and Tissue Kit	QIAGEN	Cat# 69506
QIAamp DNA Mini Kit	QIAGEN	Cat# 51306
MiRneasy mini kit	QIAGEN	Cat# 217004
GoTaq DNA polymerase	Promega	Cat# M7808
GoTaq Flexi DNA polymerase	Promega	Cat# M7808
SuperScript III Reverse Transcriptase	ThermoFisher Scientific	Cat#18080093
Illumina Nextera Rapid Capture Exome kit	Illumina	Cat# FC-140-1003
Quant-IT dsDNA BR	Thermo Fisher Scientific	Cat# Q33130
KAPA Library Quantification Kit Illumina	KAPA Biosystems	Cat# KK4873
DNA 1000 Kit	Agilen	Cat# 5067-1504
TruSeq Stranded Total RNA HT kit with Ribo-Zero Gold	Illumina	Cat# RS-122-2303
RNA 6000 Nano Kit	Agilen	Cat# 5067-1511
PhiX control	Illumina	Cat# FC-110-3001
SuperScript IV First-Strand Synthesis System	ThermoFisher Scientific	Cat#18091050
Deposited Data		
Aligned DNA and RNA sequencing data		Deposited at European Genome Archive (EGA) with ID: EGAS00001002703
Additional supplemental figures		Deposited in Mendeley Data repository. https://doi.org/10.17632/6cv77bry6m.1

(*Continued on next page*)

Continued

REAGENT or RESOURCE	SOURCE	IDENTIFIER
Software and Algorithms		
bcl2fastq2 2.17	Illumina	https://support.illumina.com/sequencing/sequencing_software/bcl2fastq-conversion-software.html
R 3.2.2	R Core Team., 2017	http://www.r-project.org
MATLAB version 9.2	1994-2017 The MathWorks, Inc.	https://www.mathworks.com/products/matlab.html
BWA Mem v0.7.15	Li and Durbin, 2009	http://bio-bwa.sourceforge.net/
GATK 3.4.46	DePristo et al., 2011	https://software.broadinstitute.org/gatk/
HaplotypeCaller	HaplotypeCaller	https://software.broadinstitute.org/gatk/documentation/tooldocs/3.8-0/org_broadinstitute_gatk_tools_walkers_haplotypecaller_HaplotypeCaller.php
Novosort 3.02	Novocraft	http://www.novocraft.com/products/novoalign/
Novoalign 3.02	Novocraft	http://www.novocraft.com/products/novoalign/
MuTect	Cibulskis et al., 2013	https://software.broadinstitute.org/gatk/download
Strelka 1.0.14	Saunders et al., 2012	strelka@ftp.illumina.com
VEP (The Ensembl Variant Effect Predictor)	McLaren et al., 2016	http://www.ensembl.org//useast.ensembl.org/info/docs/tools/vep/index.html?redirectsrc=//www.ensembl.org%2Finfo%2Fdocs%2Ftools%2Fvep%2Findex.html
Integrative Genomics Viewer (IGV)	Robinson et al., 2011	http://software.broadinstitute.org/software/igv/
Picard v2.2.1	Picard	https://broadinstitute.github.io/picard/
samtools v1.3.1	Li et al., 2009	http://www.htslib.org/
ea-utils v1.1.2	Ea-utils	https://github.com/ExpressionAnalysis/ea-utils
Bioconductor 3.2	Huber et al., 2015	http://www.bioconductor.org
Bioconductor package QDNaseq 1.2.4	Scheinin et al., 2014	http://www.bioconductor.org
GISTIC2.0	Mermel et al., 2011	https://cloud.genepattern.org/gp/landingpage/index.html
iC10: A Copy Number and Expression-Based Classifier for Breast Tumors	Ali et al., 2014	https://rdrr.io/cran/iC10/
pam50: PAM50 classifier for identification of breast cancer	Parker et al., 2009	https://rdrr.io/bioc/genefu/man/pam50.html
R package deconstructSigs 1.8.0	Rosenthal et al., 2016	http://www.r-project.org/
ASCAT 2.5	Van Loo et al., 2010	https://www.crick.ac.uk/peter-van-loo/software/ASCAT
PyClone 0.12.7	Roth et al., 2014	http://www.shahlab.ca
EnsDb.Hsapiens.v75	Rainer, 2016	http://bioconductor.org/packages/release/data/annotation/html/EnsDb.Hsapiens.v75.html
POLYSOLVER	Shukla et al., 2015	https://software.broadinstitute.org/cancer/cga/polysolver
pVAC-Seq pipeline	Hundal et al., 2016	https://github.com/griffithlab/pVAC-Seq
Immunophenogram	Charoentong et al., 2017	https://github.com/mui-icbi/Immunophenogram
MEDICC (devel branch, commit da7ed4a)	Schwarz et al., 2014	https://bitbucket.org/rfs/medicc
superFreq 0.9.17	Flensburg et al., 2018	https://github.com/ChristofferFlensburg/cnv-caller
treeomics 1.7.3	Reiter et al., 2017	https://github.com/johannesreiter/treeomics
OncoNEM 1.0	Ross and Markowetz, 2016	http://bitbucket.org/edith_ross/onconem/src
Tree: Raxml v8.2.1	Stamatakis, 2014	
VarScan 2.4.3	Koboldt et al., 2012	http://dkoboldt.github.io/varscan/
alleleCount 3.1.1	alleleCount	https://github.com/cancerit/alleleCount
QUASR	Watson et al., 2013	https://sourceforge.net/projects/quasr/
BLAST	Altschul et al., 1990	ftp://ftp.ncbi.nlm.nih.gov/blast/executables/blast+/LATEST/
IMGT	Lefranc, 2011	http://www.imgt.org/
Primer Design: mprimer (v1.9), primer3 (v2.3.7), *in silico* PCR	Koressaar and Remm, 2007; Shen et al., 2010; Untergasser et al., 2012	N/A

(*Continued on next page*)

Continued

REAGENT or RESOURCE	SOURCE	IDENTIFIER
ggplot2 2.2.1	ggplot2	https://ggplot2.tidyverse.org
Igraph 1.0.1	Igraph	https://cran.r-project.org/web/packages/igraph/index.html
Ape 4.1	Paradis et al., 2004	https://cran.r-project.org/web/packages/ape/index.html
Dendextend 1.5.2	Galili, 2015	https://cran.r-project.org/web/packages/dendextend/index.html
nonnegative matrix factorization	Gaujoux and Seoighe, 2010	https://cran.r-project.org/web/packages/NMF/index.html
limSolve	Soetaert et al., 2009	https://cran.r-project.org/web/packages/limSolve/index.html
Other		
Adaptive 587 cohort data		https://genomemedicine.biomedcentral.com/articles/10.1186/s13073-015-0238-z
Silhouettes		http://silhouettesfree.com/download-silhouette/liver-silhouette/

CONTACT FOR REAGENT AND RESOURCE SHARING

Further information and requests for resources and reagents should be directed to and will be fulfilled by the Lead Contact, Carlos Caldas (Carlos.caldas@cruk.cam.ac.uk).

EXPERIMENTAL MODEL AND SUBJECT DETAILS

Patients

Ten metastatic breast cancer patients who underwent post-mortem warm autopsies were included in this study. Nine patients were enrolled as part of the Vall d'Hebron Institute of Oncology (VHIO) Warm Autopsy Program, and one patient was enrolled at the Cambridge University Hospitals NHS Foundation Trust, Cambridge, UK as previously described (Murtaza et al., 2015).

Informed consent was obtained for all patients. Research autopsies were performed under VHIO Warm Autopsy Program protocols approved by the institutional review board (IRB) of Vall d'Hebron University Hospital (Barcelona, Spain) and under a study protocol approved by the Cambridgeshire Research Ethics Committee (Cambridgeshire 3 REC 07/Q0106/63MN.A).

METHOD DETAILS

Sample Nomenclature

Each sample identifier (ID) follows the nomenclature NNN-sample, where NNN denotes the patient ID. For whole exome sequencing (WES) and T cell receptor (TCR) sequencing, NNN-0XX indicates sample type (solid tumor or body-fluid derived DNA) (Table S1). For targeted amplicon sequencing and shallow whole genome sequencing (sWGS), the sample nomenclature is specified in Table S1. Briefly, this was derived from the primary tumor (PR), metastasis (year sample was taken (i.e., −13), followed by ID)) or body-fluid derived cell-free DNA (plasma, CSF, ascitic fluid, pleural fluid).

Nucleic acid extraction

The histologic evaluation of each diagnostic primary tumor or metastatic lesion from autopsy of the nine VHIO patients was confirmed on review of routine hematoxylin and eosin-stained slides. Samples were processed as previously described (De Mattos-Arruda et al., 2015). DNA and RNA were isolated from tumor tissue using commercially available kits according to manufacturer's specifications (Key Resources Table). DNA was extracted from peripheral blood mononuclear cells and body fluids using commercially available kits as per manufacturer's specifications (Key Resources Table). RNA and DNA were quantified using the Qubit Fluorometer (Invitrogen).

The collection, processing, DNA extraction and preparation of exome-sequencing libraries of DET52 patient for tumor tissues and plasma samples have been described previously (Murtaza et al., 2015).

WES AND sWGS ANALYSES

DNA sequencing

Libraries for Illumina sequencing were prepared using Illumina Nextera Rapid Capture Exome kit (cat. FC-140-1003, Illumina). Prior to library preparation DNA concentrations for each sample were quantified using a fluorescence-based method (Quant-IT dsDNA BR, cat. Q33130, Thermo Fisher Scientific) and 50ng of genomic DNA was used for library preparation.

Samples were processed following manufacturer's instructions (part# 15037436 Rev. J, Illumina) for WES and sWGS. Prior to first hybridization all libraries were quantified using quantitative polymerase chain reaction (qPCR). KAPA Library Quantification Kit

(cat. KK4873, KAPA Biosystems) as used as per manufacturer's recommendations. A subset of libraries was analyzed using DNA 1000 Kit (cat. 5067-1504, Agilent).

Whole genome libraries and exome libraries were normalized and pooled in equal volumes to create balanced pools. Each pool was normalized to a molarity of 4nM and used for sequencing with clustering concentration 20pM with 1% spike-in of PhiX control. Sequencing was performed on an Illumina HiSeq2500 using v4 chemistry and 50 cycles single-end for s-WGS and 75 cycles paired-end for WES.

Demultiplexing was performed using Illumina's bcl2fastq2 v.2.17 software using default options. FASTQ files were used for subsequent data analysis.

WES analyses

Adaptor and low-quality base (Phred score below 20) trimming, alignment to the GRCh37 build of the human genome, and base quality recalibration were performed using Novoalign v 3.02 (Novocraft). Coordinate sorting of reads and PCR-duplicate marking was performed using Novosort (v 3.02). The resulting bam files for all samples for the same patient were locally realigned using the Genome Analysis Toolkit (GATK, v 3.4.46) (DePristo et al., 2011). MuTect (version 1.1.7) was run using default parameters (Cibulskis et al., 2013). In order to decrease the false positive rate secondary to germline variants of noisy regions within the genome, a panel of normals derived from 300 normal tissue exomes was generated using MuTect's artifact detection mode and supplied to MuTect during variant calling. Strelka (version 1.0.14) (Saunders et al., 2012) was run with recommended starting parameters for BWA and default parameters. The isSkipDepthFilters parameter was set to 1 to skip depth filtration. Only tier 1 mutations, as well as SNVs with a QSS_NT score higher than 15 and Indels with a QSI_NT score higher than 30 were retained. Mutations present in all samples for each patient were then concatenated into one VCF, and Haplotypecaller was used in GENOTYPE_GIVEN_ALLELES mode to detect these mutations across all samples.

SNVs and indels that fell into any of these categories were removed:

- Rejected by MuTect for a reason different than "DBSNP Site," "DBSNP Site,alt_allele_in_normal" or "alt_allele_in_normal." Mutations rejected by MuTect by any of these reasons that were present in the 1000 Genomes Project were also rejected
- Read depth less than 10
- Variant allelic frequency less than 0.05 in all samples for one patient
- Minimum allelic frequency of 0.02 per sample
- SNVs falling in segmental duplications regions, as annotated by annovar (Wang et al., 2010) genomic superDups, were classified as potential artifacts. Those that occurred in more than one patient, or in only one patient but in less than 25% of the samples from that patient were removed
- SNVs falling in simple repeat regions, microsatellites or homoplymers were removed.

Somatic mutations were annotated using Variant Effect Predictor (VEP, http://grch37.ensembl.org/index.html) (McLaren et al., 2016) and visualized using IGV (Robinson et al., 2011).

sWGS analyses

50 bp single-read sWGS was performed to provide copy number profiles. FASTQ files were aligned to the GRCh37 assembly of the human genome using BWA (Li and Durbin, 2009) and the bam files were merged, sorted and indexed using samtools (Li et al., 2009). Duplicates were marked using Picard. Copy number profiles were obtained using the R package QDNaseq (Olshen et al., 2004), using non-overlapping 100 kb pairs windows, and correcting for mappability and GC content.

Targeted amplicon sequencing

Targeted amplicon sequencing (TS) was performed in 464 unique amplicons derived from WES across 10 patients (average 46.4/case, range 16-127). Targeted sequencing libraries were prepared using droplet-based PCR amplification following the manufacturer's protocols for ThunderBolts Cancer Panel with specific modifications (RainDance Technologies) as previously reported (Murtaza et al., 2015). Multiplex primers sequences are shown in Table S6. Analysis of targeted sequencing data was performed as described previously (Murtaza et al., 2015). For each locus and non-reference allele of interest, we assessed the allele fraction in eight control genomic DNA samples. A mutation was considered significantly detectable if minimum coverage at that locus was 500x, and the AF in a sample was greater than or equal to 3 standard deviations higher than the mean AF in control samples, and if present in greater than or equal to 1% allelic fraction (AF). Additionally, any samples in which over 90% of all mutations had AFs below 5% were excluded. The mutation calls generated from targeted sequencing were then used to assess the quality of the exome mutation calling pipeline. Any mutations detected on both WES and TS were defined as true positives (TP), mutations detected on WES, but not TS defined as false positives (FP), mutations detected on TS but not on WES defined as false negatives (FN), and finally mutations that were not detected on TS and WES defined as true negatives (TN). Sensitivity was subsequently calculated as TP/(TP+FN), specificity calculated as TN/(TN+FP), precision calculated as TP/(TP+FP) and accuracy calculcated as (TP+TN)/(TP+FP+FN+TN).

Genotyping

Germline SNPs and indels were identified in all samples using GATK HaplotypeCaller, and all tumor samples were then genotyped to the matching normal tissues by computing the percentage of shared SNPs and indels between tumors and normal. Concordance of more than 90% was taken to indicate related samples.

RNA-sequencing analysis

RNA sequencing

RNA sequencing libraries were prepared using the TruSeq Stranded Total RNA HT kit with Ribo-Zero Gold (cat. RS-122-2303, Illumina). Prior to library preparation samples were quantified using a fluorescence based method and RNA quality was assessed using RNA 6000 Nano Kit (cat. 5067-1511, Agilent) on Bioanalyzer2100 (Agilent). Depending on availability, 400-900ng of total RNA was used for library preparation. The RNA Integrity Number (RIN) for these samples varied from 2.3 to 7.2. Samples were processed following manufacturer's HS (High-Sample) instruction (part# 15031048 Rev. E, Illumina). Subset of 12 libraries was analyzed using DNA 1000 Kit (cat. 5067-1504, Agilent) and the average library length was determined as 280bp. All libraries were quantified using qPCR. Serial dilutions were made and 100,000x dilution was used for quantification using KAPA Library Quantification Kit Illumina (cat. KK4873, KAPA Biosystems). Libraries were normalized to 40nM and pooled in equal volumes to create a balanced pool. The library pool was quantified after doing serial dilutions in triplicate and 10,000x and 100,000x dilutions were used for quantification. The final library was normalized to 4nM and sequenced at a clustering concentration of 20pM and 22pM with 1% spike-in of PhiX control (cat. FC-110-3001, Illumina). Sequencing was performed on HiSeq2500 v4 chemistry single-end flow cell (Illumina) following manufacturer's instructions. Demultiplexing was performed using bcl2fastq2 v.2.17 software (Illumina) using default options.

RNA sequencing analyses

FASTQ files were aligned to the GRCh37 assembly of the human genome using STAR v 2.5.2b in two-pass mode for splice-aware read alignment. Counting of reads aligned over exonic features for the purpose of differential expression was performed using the htseq-count script in the HTSeq package (v 0.6.1) in 'Union' overlap resolution mode using a Gene Transfer Format (GTF) file from Ensembl (http://www.ensembl.org//useast.ensembl.org/?redirectsrc=//www.ensembl.org%2F). The gene counts for all samples were then collated and FPKM calculations per gene per sample performed using the rpkm() function in the edgeR R package. De novo transcript assembly and counting of transcripts was performed using Cufflinks v2.2.1.

For variant calling, STAR-aligned BAM files were processed as per the RNA-seq GATK Best Practices. Briefly, sequencing read duplicates were marked using Picard MarkDuplicates, followed by Split'N'Trim and mapping quality reassignment using GATK SplitNCigarReads (v3.6). This was then followed by local realignment across indels and base quality recalibration using GATK. Mutations detected in the corresponding DNA sequencing data were genotyped in RNA using the GENOTYPE_GIVEN_ALLELES mode in Haplotype caller.

Selection of driver mutations

Breast cancer driver mutations were defined as those genes identified in previous publications (Lefebvre et al., 2016; Nik-Zainal et al., 2016; Pereira et al., 2016) and non-breast cancer drivers were defined as those present at the Cancer Gene Census and non-overlapping with breast cancer driver mutations (https://cancer.sanger.ac.uk/census/) (Table S4).

Mutational signatures

Somatic substitutions of each metastatic sample were organized into a 96-channel vector (where the six mutation classes and their immediate flanking sequence context are taken into account), referred to hereafter as a mutational profile. Mutational signature analysis of these mutational profiles was performed in two steps: extraction and assignment.

The first step in our analysis aimed to identify any signatures previously found in associated primary tumors that are present within our cohort. It consisted of applying the widely adopted (Alexandrov et al., 2013) Non-negative Matrix Factorization (NNMF) algorithm (R-CRAN package NMF - (Gaujoux and Seoighe, 2010)) to an extended dataset, where 240 additional WES metastatic breast cancer samples (Lefebvre et al., 2016) were added to our original 86 sample cohort. NNMF extraction was performed on these mutational profiles, bootstrapped 100 times, and a KL-divergence error was used to assess the accuracy of each result. The rank of the NNMF solution (i.e., the number of extracted signatures), was allowed to vary between 2-20. Across the different extractions, cosine similarity comparison with known canonical primary tumor signatures (COSMIC) revealed the presence of Signatures 1,2,13, and 17. The use of the additional samples increased the power of the NNMF, enabling a more precise mutational profile extraction for these four well-known breast-cancer-related signatures.

The second step consisted of assigning the contribution of the four COSMIC signatures identified (Signature 1, 2, 13, 17) to each sample of our original 86 metastatic cohort. This was computed using a quadratic programming algorithm (R-CRAN package limSolve - (Soetaert et al., 2009)). A minimum of either 3% of the total number of mutations of the sample or at least 10 mutations was required for a COSMIC signature to be attributed to a sample. The assignment step was performed on four versions of the mutational profiles for each patient, including: (i) all mutations, (ii) mutations shared across all samples (stem), (iii) mutations shared across some samples (clade), and (iv) mutations uniquely present in one sample (private).

IntCluster, PAM50 and stratification into breast cancers subtypes

Matched samples (n = 60) with copy number and expression data were classified into one of the 10 Integrative Clusters using the 'iC10' R package (Ali et al., 2014; Curtis et al., 2012). The 'iC10' package uses copy number and expression from breast cancer data, trains a pamr classifier with the features available and predicts the iC10 group. Each sample was classified into the 10 Integrative Clusters and the assignment to each model was done by consensus after manual curation in specific cases. The 50-gene subtype predictor PAM50 was also applyied to 64 metastases with expression data using the R package genefu (Parker et al., 2009).

ASCAT

ASCAT v2.5 (Van Loo et al., 2010) was run by integrating copy number log ratios generated from QDNaseq and SNP allelic frequencies from WES data. The gamma technology parameter was set to 1 as recommended for exome sequencing.

Phylogenetic analyses

Four multi-sample methods were used to infer the metastatic breast cancer phylogenies. MEDICC (Minimum Event Distance for Intra-tumor Copy number Comparisons) is a method for phylogenetic reconstruction and heterogeneity quantification that uses allele-specific copy number profiles (Schwarz et al., 2014). OncoNEM (Ross and Markowetz, 2016) and Treeomics (Reiter et al., 2017) are tools that utilize Bayesian inference to infer phylogenetic relationships from mutation patterns of SNVs. SuperFreq is a clonality tracker that uses single nucleotide variants (SNV) and copy number alterations (CNA) (Flensburg et al., 2018; Savas et al., 2016).

MEDICC

QDNaseq (Scheinin et al., 2014) was applied to the sWGS data to obtain sequence mappability and GC content adjusted log ratios of read depth. B-allele frequencies were calculated from WES data at previously inferred germline variant sites using alleleCounte 3.1.1. Log ratios and B-allele frequencies were segmented on a per case basis using allele specific multi-sample segmentation (Ross et al., 2017) and allele-specific copy number profiles were inferred using ASCAT 2.5 (Van Loo et al., 2010). The raw ASCAT copy number profiles were compared across samples for each case and ASCAT was rerun with adjusted ploidy and/or purity estimates where necessary to obtain the final discrete copy number profiles. Samples with copy number fits of low quality were excluded. A maximum copy number cut-off of nine was applied to both major and minor copy number profiles, replacing any values exceeding this threshold to comply with MEDICC requirements. Finally, MEDICC was used to infer the phylogenies (Schwarz et al., 2014).

Treeomics

Treeomics 1.7.3 (Reiter et al., 2017) was used to infer phylogenies from both WES and targeted sequencing data. For each case, Treeomics was used to calculate the posterior probabilities of a variant being present based on total read depth and number of reads covering the alternative allele. To make the Treeomics analysis computationally feasible, the number of samples had to be reduced for some of the cases. In order to keep the mutation profiles as diverse as possible and to maintain a good representation of the different tumor populations, samples that had a mutation profile similar to one of the remaining samples were excluded preferentially. Additionally, all sites whose posterior probabilities were lower than 0.5 in all samples were removed, as these were likely to be false positives. Finally, Treeomics was applied to each case with subclone detection switched on and all other parameters set to default.

OncoNEM

Like Treeomics, OncoNEM 1.0 (Ross and Markowetz, 2016) was used to infer phylogenies from both WES and targeted sequencing data. Binary mutation profiles were obtained from the Treeomics posterior probability matrices by setting all entries with a mutation probability smaller than 0.5 to 0 and to 1 otherwise. The OncoNEM analysis was performed using error parameter optimization over a parameter range from 0.0001 to 0.1. The Bayes factor threshold epsilon was set to 2.

SuperFreq

SuperFreq was used to infer the clonal composition of the different samples from WES data. Pileup files of the WES data were generated using samtools 1.3.1 mpileup using a maximum depth threshold of 10000, a minimum mapping quality of 1 and a minimum base quality of 15. Liberal variant calling was performed using VarScan 2.4.3 mpileu2cns with a p value filter of 0.01, no strand-bias filter and the variant flag set to only obtain variant sites. Then SuperFreq 0.9.17 was run with default parameters using the normal of all cases apart from DET52 as reference normal samples.

PyClone

PyClone is a Bayesian clustering method that infers the clonal population structures for each sample (Murtaza et al., 2015; Roth et al., 2014). It integrates mutation alleles, copy number calls for each sample as input to obtain cellular frequencies for each cluster in each sample. PyClone was run using a beta- binomial density, using 40000 iterations and a burn-in sample of 20000. A minimum cluster size of 3 was selected for WES data.

LICHeE

The previously inferred PyClone clusters were used as input for LICHeE. To remove spurious clones from the PyClone output two filtering steps were performed. First, low prevalence clones that did not exceed a cellular prevalence of 0.1 in any of the samples were removed. Second, if multiple clusters were present in all samples, all but the cluster with the highest cellular prevalence were removed. To generate binary presence-absence patterns of mutation clusters across samples, a mutation set was classified as present in a given sample if at least 40% of its mutations had a VAF larger than 0.01. Finally, LICHeE (commit 238770c) was used to infer clone trees and sample compositions, assuming a cellular prevalence estimate error of 0.3.

Cytolytic activity score

Cytolitic activity score was calculated as the geometric mean of the *GZMA* and *PRF1* expression levels from RNA gene expression data (Rooney et al., 2015).

Immunophenogram and immunophenoscore calculations

The immunophenogram (Charoentong et al., 2017) was applied to determine the immunophenotypes of each tumor sample and to enable the calculation of an aggregated score (immunophenoscore (IPS)), based on the expression of major determinants. These factors were classified into four categories: MHC molecules (MHC), Immunomodulators (CP), Effector cells (EC), Suppressor cells (SC), and into 20 single factors (MHC molecules, immunoinhibitors, and immunostimulators) and six cell types (effector cells: activated CD4+ T cells, activated CD8+ T cells, effector memory CD4+ T cells and effector memory CD8+ T cells; suppressive cells: Tregs, and MDSCs).

Briefly, the algorithm generates normalized Z scores from gene expression data for a list of cancer immunity parameters (using an input list of 162 genes (Table S5)). The outer part of the wheel illustrates sample-wise (averaged) Z scores, which is calculated for each

of the individual 26 parameters. These Z scores are positively weighted according to stimulatory factors (cell types) and negatively weighted according to inhibitory factors (cell types) and averaged. The inner wheel illustrates the weighted Z scores of the factors included into the four categories. Z scores $\geq$ 3 were designated as IPS10 and z-scores $\leq$ 0 are designated as IPS0.

Unsupervised hierarchical clustering based on the Euclidean distance matrix of the Z scores across metastases (R heatmap.3 function) were used to produce the heatmaps.

HLA typing and in silico neoantigen prediction pipeline

For the metastases of all patients, the 4-digit HLA type was determined using POLYSOLVER (POLYmorphic loci reSOLVER) as previously described (Shukla et al., 2015).

The pVAC-Seq pipeline (Hundal et al., 2016) was used with minor modifications. All nonsynonymous point mutations identified were translated into strings of 17-21 amino acids with the mutant amino acid situated centrally. A sliding window method was used to identify amino acid substrings within the mutant 17-mer that had a predicted MHC Class I binding affinity of $\leq$ 500nM to one (or more) of the patient-specific HLA alleles. Binding affinity for the mutant and corresponding wild-type nonamer were analyzed using the NetMHCPan v3 prediction tool bundled within the IEDB MHC-I binding prediction resources. Following this, for all cases excluding DET52 (which did not have any RNA expression data), candidate neoantigens were further filtered by retaining mutations that were also present in the RNA sequencing data, as well as had a gene and transcript RPKM expression of more than 1. Neoantigens were subsequently classified as stem, private and clade by applying the classification derived from the WES mutational dataset. When generating Figure 5A, highly similar neoantigens generated from one mutation were counted as one.

Neo-antigen simulations

Simulations procedure

We assumed that each gene has its own mutation rate. For each gene, we used the mutations list of 4,742 WES tumor normal pairs from (Lawrence et al., 2014) (http://www.tumorportal.org) to estimate the gene relative background mutation rate by counting the amount of mutations the gene had divided by the total number of mutations. Every mutation was then randomly assigned to a new gene based on the gene's relative rate. The position within the gene was chosen to maintain the trinucleotide context of each mutation (the 5′ and 3′ nearest neighbors and the mutated position) and the variant was based on the original mutation. In addition, for every base we counted how many times it was sufficiently covered for mutation detection (i.e., > = 14 reads), across 7,732 TCGA tumor WES samples. The fraction of covered patients at a given base was used as a probabilistic weight when selecting the new position for a mutation.

P value calculation

For a single sample analysis we defined the *P*-value as the fraction of replications (out 100) that had a neo-epitopes ratio larger than 1. For combining cases based on their target tissue we tried to see if their average was less expected at random. For that we generated 20 replicates and and 100 additional simulations based on each of the 20 replicates. We then calculated the average neo-epitopes ratio for each of these sets and compared it to the original ratio. We then calculated a P value for the fraction of the real average being different from the simulations average.

Loss of heterozygosity in human leukocyte antigen (LOHHLA)

LOH over the HLA Class I locus was identified using LOHHLA on the whole exome sequencing data, and LOH called if the copy number at HLA-A/HLA-B or HLA-C locus was less than 0.5, with a p value of less than 0.05 (McGranahan et al., 2017).

T cell receptor (TCR) analysis

TCR-sequencing library preparation

Reverse transcription primers were designed using Primer3 (Koressaar and Remm, 2007; Untergasser et al., 2012) and multiplex PCR primers using MPrimer (Shen et al., 2010). cDNA synthesis was performed using TCR-α or TCR-β constant region-specific primers carrying a molecular barcode of 12 random/degenerate nucleotides (N12, TNNNNTNNNNTNNNNT) to enable molecule-level identification (unique molecular identifier). The molecular barcode was inserted upstream (5′) of the sequence that recognizes the constant region(s) and downstream (3′) of an adaptor sequence complementary to first round PCR reverse primers.

Reverse transcription was performed with SuperScript IV First-Strand Synthesis System (Invitrogen, ThermoFisher Scientific) using 500ng to 2 μg of total RNA, 1 μL of barcoded TCR-α or TCR-β specific reverse primer (0.1 μM final), 1 μL of dNTPs (0.5mM final) and added with nuclease-free water to a total volume of 13 μl. This was incubated at 65°C for 5min, then on ice for 2 minutes and followed by addition of 4 μL of 5X First strand buffer, 1 μL of DTT (5mM final), 1 μL of RNaseOUT Recombinant Ribonuclease Inhibitor (40 units), and 1 μL of SuperScript IV reverse transcriptase (200 units). The reaction was incubated at 56°C for 60min followed by inactivation at 80°C for 10min. 1 μL of *E. coli* RNase H (2 units) was added and incubated at 37°C for 20min to remove RNA from the cDNA:RNA hybrids. The first strand cDNA was cleaned up using 1.8x Agencourt Ampure XP beads (Beckman Coulter) and eluted with 21 μL of nuclease-free water (Ambion).

First-round multiplex PCR amplifications were set up in a total volume of 50 μl, with 20 μL of cDNA as template, 25 μL of Q5 Hotstart High-Fidelity DNA Polymerase Master Mix 2x (New England Biolabs) and tailed TCR-α or TCR-β forward primer set pools and sample-indexed reverse primers (0.2uM final concentration each). Multiplex forward primers target different TCR-α or TCR-β V-regions and sequences are shown in Table S7. The sample-indexed reverse primers used were published previously (Bronner et al., 2014). The following PCR program was used: 30 s at 98°C, 25 cycles of 20 s at 98°C, 1 min at 55°C, and 1 min at 72°C, with a final extension

step of 2 min at 72°C. The PCR product was cleaned up using 0.75x Agencourt Ampure XP beads (Beckman Coulter) and eluted with 20 µL of nuclease-free water (Ambion).

First-round forward (TCR-α or TCR-β V-region-specific) PCR primers each contained a shared sequence to allow Illumina sequencing adapters to be introduced with a second round PCR. The second-round PCR amplification step was performed on the first round PCR amplicons to generate Illumina-ready sequencing libraries. 12 cycles of PCRs were performed and the product was analyzed and quantitated using Agilent Bioanalyzer DNA 1000 chips. For each batch, equal nanomoles of each sample were pooled, double SPRI size selected (0.5x and 0.7x) and stored at −20°C until sequencing. Libraries were batched and sequenced on MiSeq sequencers (300bp paired-end reads).

TCR-sequencing and barcode filtering

MiSeq libraries were prepared using Illumina protocols and sequenced using 300bp paired-ended MiSeq (Illumina). Raw MiSeq reads were filtered for base quality (median Phred score > 32) using the QUASR program (https://sourceforge.net/projects/quasr/) (Watson et al., 2013). MiSeq forward and reverse reads were merged together if they contained identical overlapping region of > 50bp, or otherwise discarded. Universal barcoded regions were identified in reads and orientated to read from V-primer to constant region primer. The barcoded region within each primer was identified and checked for conserved bases (i.e., the T's in NNNNTNNNNTNNNNT). Primers and constant regions were trimmed from each sequence, and sequences were retained only if there was > 80% sequence certainty between all sequences obtained with the same barcode, otherwise discarded. The constant region allele with highest sequence similarity was identified by k-mer matching (where k = 10bp) to the reference constant region gene IMGT database (Lefranc, 2011), and sequence trimmed to give only the region of the sequence corresponding to the variable (V-D-J) regions, where constant region usage information for each TCR was retained throughout the analysis. Sequences without complete reading frames and non-TCR sequences were removed and only reads with significant similarity to reference TRBV or TRAV and J genes from the IMGT database were retained using BLAST (Altschul et al., 1990). Sequences were annotated using IMGT. Sample clustering was performed as previously described (Bashford-Rogers et al., 2013).

TCR repertoire generation and network analysis

The network generation algorithm and network properties were calculated as in Bashford-Rogers et al.(Bashford-Rogers et al., 2013): each vertex represents a unique sequence, where relative vertex size is proportional to the number of identical reads. A clone (cluster) refers to a group of identical related T cells, each containing TCRs with identical CDR3 regions and TCRV gene usage.

Repertoire parameters that were dependent on sequencing depth were generated by subsampling each sequencing sample to a specified depth and the mean of 20 repeats of resulting parameters were calculated using the clonality measures. These measures include 1) total repertoire clonality (vertex & cluster Gini Indices), and 2) mean cluster sizes, (3) largest cluster sizes calculated as follows:

1) Total repertoire clonality, measured by vertex & cluster Gini Indices are defined in Bashford-Rogers et al. (Bashford-Rogers et al., 2013), calculated from the distribution of the number of unique RNA molecules per vertex and the distribution of the number of unique TCRs per cluster, respectively. These were all calculated per 1000 read subsample of the each total TCR repertoire.
2) Mean cluster sizes (MCS) were within each subsample for the total TCR repertoire were calculated as follows for any given sample Y:

$$\mathrm{MCS(Sample\ Y)} = \frac{\sum N\ TCRs\ per\ cluster\ in\ subsample}{\sum Total\ number\ of\ clusters\ in\ subsample} x100$$

3) Largest cluster sizes (LCS) were within each subsample for the total TCR repertoire were calculated as follows for any given sample Y:

$$\mathrm{MCS(Sample\ Y)} = \frac{Max(N\ TCRs\ per\ cluster\ in\ subsample)}{\sum Total\ number\ of\ clusters\ in\ subsample} x100$$

Clonal overlap analysis

Clonal groups defined as TCRs sharing same V and J gene usages and identical CDR3 region sequence (nucleotide). Public TCRs were defined as TCR clusters that were shared between 2 or more T cell samples from unrelated individuals within this dataset and compared to the Adaptive Biotechnology database (Dean et al., 2015). The clonal overlap coefficient, describing the overlap between samples, was calculated either considering the whole TCR datasets, or with the public TCR clones removed.

Clonal overlap, $O(i,j)$, between any two samples, i and j was calculated by

$$O(i,j) = \frac{C_{i,j}}{1/2\,(C_i + C_j)}$$

Where C_x is the number of clusters in sample x and $C_{i,j}$ is the number of clusters shared between samples i and j. To account for differences in sequencing depth between samples, each sample was subsampled each sequencing sample to a specified depth (1000 TCRs), and the mean of 20 repeats per sample of resulting clonal overlap was calculated.

Similarity heatmaps

Similarity heatmaps were produced using Jaccard index calculated between each pair of metastases, using unique amino acids TCRs (CDR3s), for the alpha and beta chains. The Jaccard index was calculated using the ecological toolkit of the vegan R package, and the heatmaps were produced using R pheatmap package. Clustering of the heatmaps was done by the standard R hclust (hierarchical clustering) method, using the "complete" option. The comparison of the hclusts objects was done by the cophenetic correlation, using the dendextend package (Galili, 2015).

The data were reshuffled to assign the TCR sequences to "randomized" metastases, and then on the reshuffled repertoire clustering by the Jaccard index was performed. This randomization was done 100 times. In this setting where there are 100 clusterings performed on the different randomizations and these were compared then between themselves, and to the original clustering of the biological data. To compare the randomized and 'real' trees we used the cophenetic correlation, and the Robinson-Foulds metric.

The comparison of the Jaccard clustering trees with the genetic trees was done by using the cophenetic definition for edge-weighted trees. In this version of the cophenetic, the distance between each pair of nodes is the sum of the edges weights along the path connecting these pair of nodes.

Correlation of TPM of CDR3 genes and sum of TCR reads

From the RNASeq we extracted the TPM values of four genes that encode for the four different parts of the CD3 complex. These genes are CD3D, CD3G, CD3E and the zeta chain CD247 (Ensembl codes: ENSG00000167286, ENSG00000160654, ENSG00000198851, ENSG00000198821, respectively). For each sample, we calculated two measurements: 1) the sum of the RNASeq TPM values for these four genes; 2) from the TCR repertoires - the sum of the number of alpha chains and beta chains for each sample. We computed the Pearson correlation between the log10 values of these two measurements across all samples. The correlation and p value were computed using R's cor.test method.

Histopathological analyses

Tissue microarrays (TMA) were prepared using duplicate 1 mm cores extracted from formalin-fixed paraffin-embedded blocks containing material from the individual tumors and metastases.

Immunohistochemistry (IHC) was conducted for CD68, CD3, CD19, FOXP3, CD8, IL3RA, IDO1, CD4, CD56, CD1A, Mast Cell Tryptase, CD45RO, CD38, PDL1, ER, PR, and HER2 proteins. Details of reagents and protocols for IHC are provided in Key Resources Table.

Stains were manually quantified by counting the absolute number or the percentage of positive stained cells. ER, PR statuses were assessed based on IHC applied to TMAs and scored using the percentage of positive tumor cells and intensity of staining (AllRead score). Herceptest was performed for all samples according to ASCO/CAP Guidelines (Wolff et al., 2013).

Fluorescence *in situ* hybridization (FISH) (HER2-to-CEP17 ratio and gene copy number) for HER2 status was performed as previously described for all samples (Wolff et al., 2013). Positive HER2 amplification was considered when FISH ratio was higher than 2.2 or HER2 gene copy greater than 6.0.

Digital pathology

Whole slide images (either FFPE sections or frozen sections from tissue samples used for RNA extraction) were analyzed using CellExtractor v1.0, an open-source platform developed for high throughput analysis of histopathological images. The code was written in Python and used the OpenCV, i.e., an open source computer vision and machine learning software library written in C++, and the OpenSlide library. Full-face H&E scanned images were analyzed and divided into several sub-regions. Each sub-region is processed and segmented to compute cellular features such as centroids, shape descriptors, and pixel intensities. These features were used to train a support-vector machine (SVM) based classifier to identify cancer cells, stromal cells, and lymphocytes based on a training set of objects selected by a pathologist (W.C.) of approximately 1,000 objects for each category. Finally, based on these classes descriptive statistical parameters such as cellular fractions and densities were estimated. For each detected cell density was obtained based on counting the number of nearest neighbor approach, i.e., the density within the distance to the Nth nearest neighbor calculated as follow: Sigma_N (pixel^[-2]) = N/(pi* d_N^2), Where d_N is the distance to the Nth nearest neighbor within a density-defining population. A value of N = 50 was used in order to estimate the density parameter (see (Ali et al., 2016b) for a detailed description).

QUANTIFICATION AND STATISTICAL ANALYSIS

All statistical analyses were performed using R version 3.2.2 and associated packages (Key Resources Table). The statistical details of experiments including the exact value of n in terms of number of samples for a given patient, the experimental method and specific statistical tests employed are reported in the relevant section, Results, Figures and Figure Legends, and Supplementary tables. For a given test (i.e., Wilcoxon rank sum, test chi-square test) significance was defined if a p value was less than 0.05.

DATA AND SOFTWARE AVAILABILITY

Software

Custom scripts to run the analyses described in the manuscript are available at https://github.com/cclab-brca/MET-breast-landscapes/

Data Resources

Sequence data has been deposited at the European Genome-phenome Archive (EGA), which is hosted by the EBI and the CRG, under accession number EGAS00001002703. Further information about EGA can be found on https://ega-archive.org. The European Genome-phenome Archive of human data consented for biomedical research (https://idp.nature.com/authorize?response_type=cookie&client_id=grover&redirect_uri=http%3A%2F%2Fwww.nature.com%2Fng%2Fjournal%2Fv47%2Fn7%2Ffull%2Fng.3312.html). Supplemental Information was deposited on Mendeley at https://doi.org/10.17632/6cv77bry6m.1

Cell Reports, Volume *27*

Supplemental Information

The Genomic and Immune Landscapes

of Lethal Metastatic Breast Cancer

Leticia De Mattos-Arruda, Stephen-John Sammut, Edith M. Ross, Rachael Bashford-Rogers, Erez Greenstein, Havell Markus, Sandro Morganella, Yvonne Teng, Yosef Maruvka, Bernard Pereira, Oscar M. Rueda, Suet-Feung Chin, Tania Contente-Cuomo, Regina Mayor, Alexandra Arias, H. Raza Ali, Wei Cope, Daniel Tiezzi, Aliakbar Dariush, Tauanne Dias Amarante, Dan Reshef, Nikaoly Ciriaco, Elena Martinez-Saez, Vicente Peg, Santiago Ramon y Cajal, Javier Cortes, George Vassiliou, Gad Getz, Serena Nik-Zainal, Muhammed Murtaza, Nir Friedman, Florian Markowetz, Joan Seoane, and Carlos Caldas

Cell Reports

Supplemental Information

The genomic and immune landscapes of lethal metastatic breast cancer

Leticia De Mattos-Arruda, Stephen-John Sammut, Edith M. Ross, Rachael Bashford-Rogers, Erez Greenstein, Havell Markus, Sandro Morganella, Yvonne Teng, Yosef Maruvka, Bernard Pereira, Oscar M. Rueda, Suet-Feung Chin, Tania Contente-Cuomo, Regina Mayor, Alexandra Arias, H Raza Ali, Wei Cope, Daniel Tiezzi, Aliakbar Dariush, Tauanne Dias Amarante, Dan Reshef, Nikaoly Ciriaco, Elena Martinez-Saez, Vicente Peg, Santiago Ramon y Cajal, Javier Cortes, George Vassiliou, Gad Getz, Serena Nik-Zainal, Muhammed Murtaza, Nir Friedman, Florian Markowetz, Joan Seoane, and Carlos Caldas

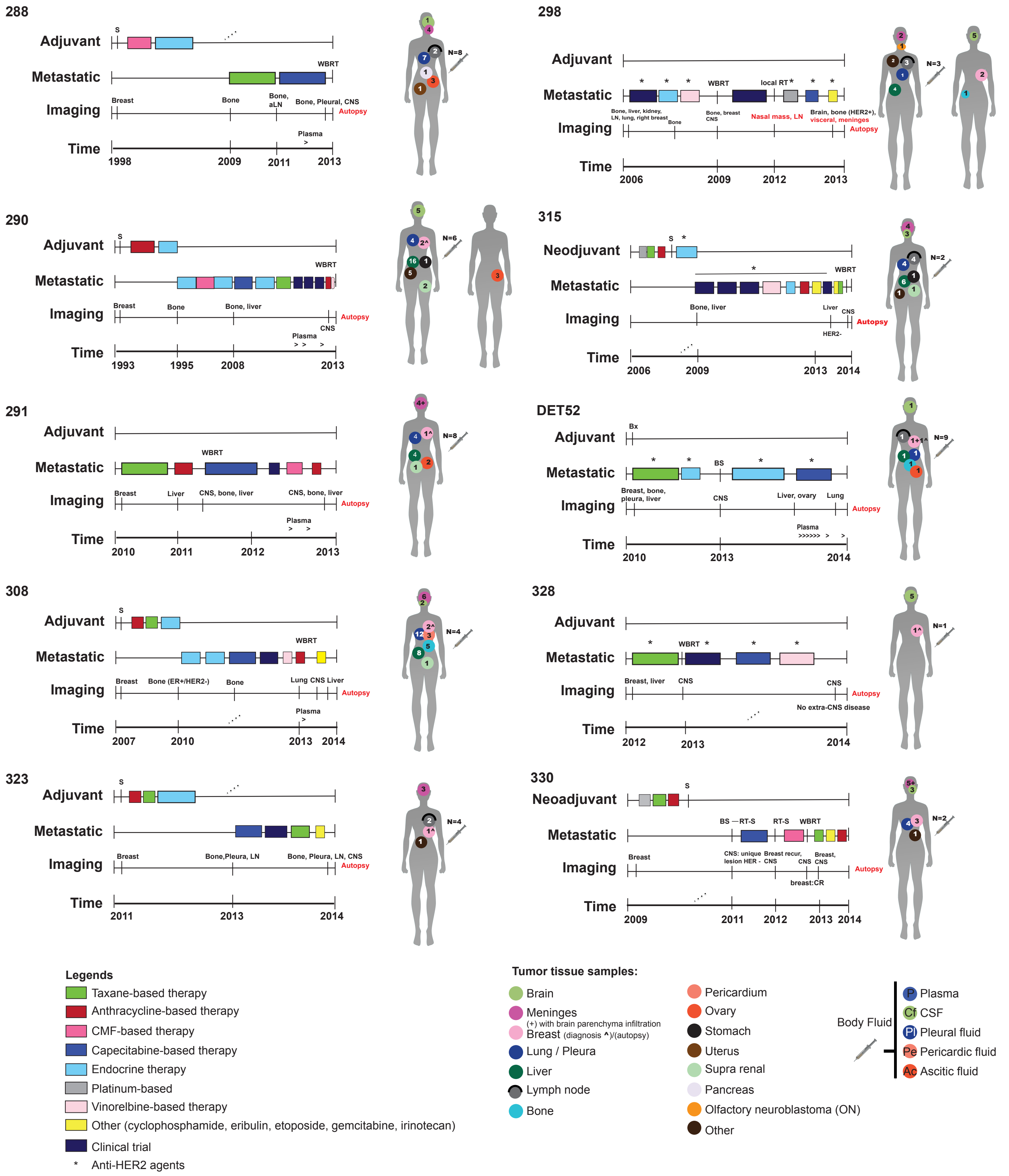

Figure S1: Timelines with clinical and treatment data for all 10 cases, related to Figure 1.

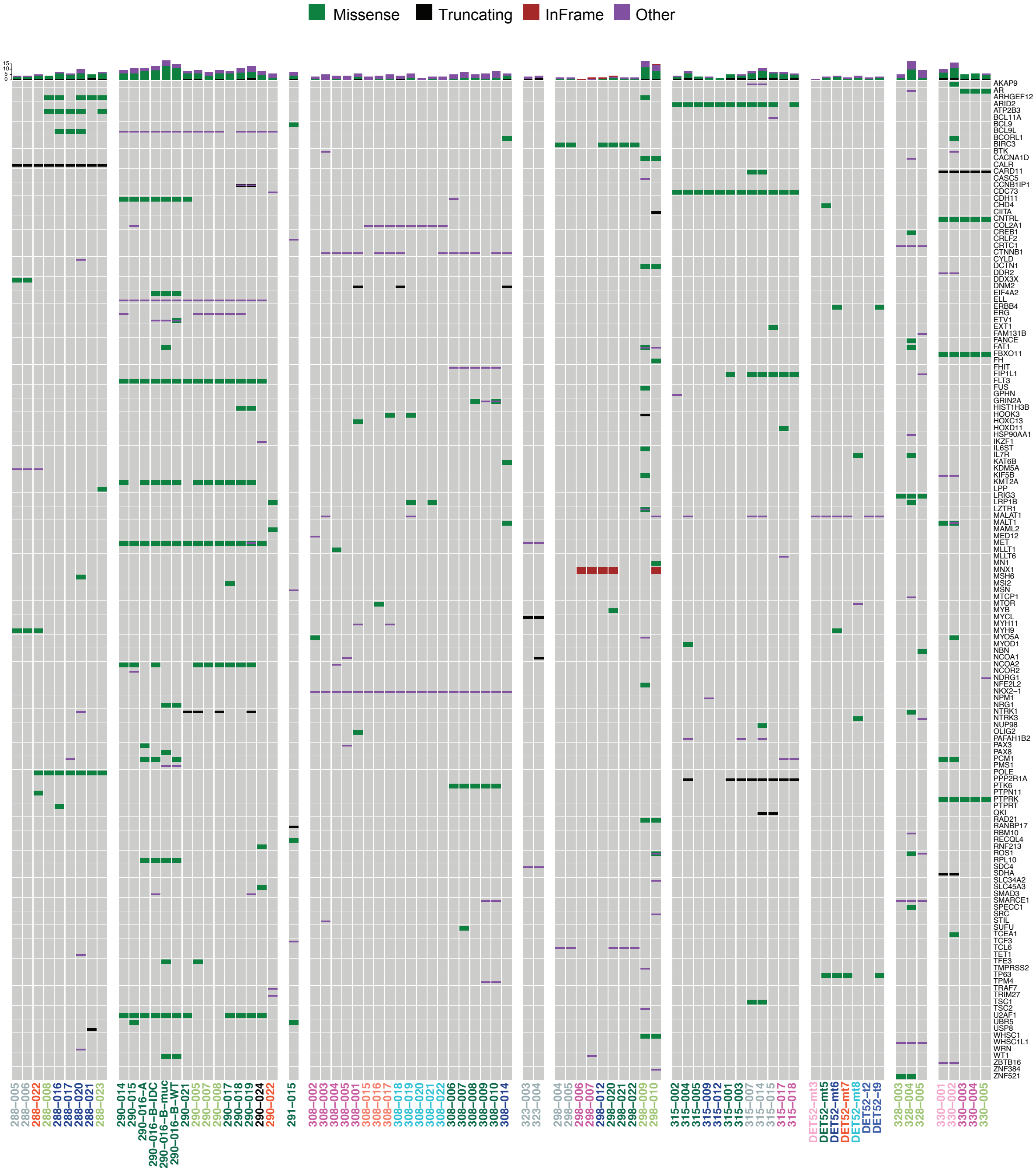

Figure S2: Oncoprint plot showing the mutations in non-breast cancer driver genes identified by WES across 85 metastases for the 10 patients, related to Figure 2.

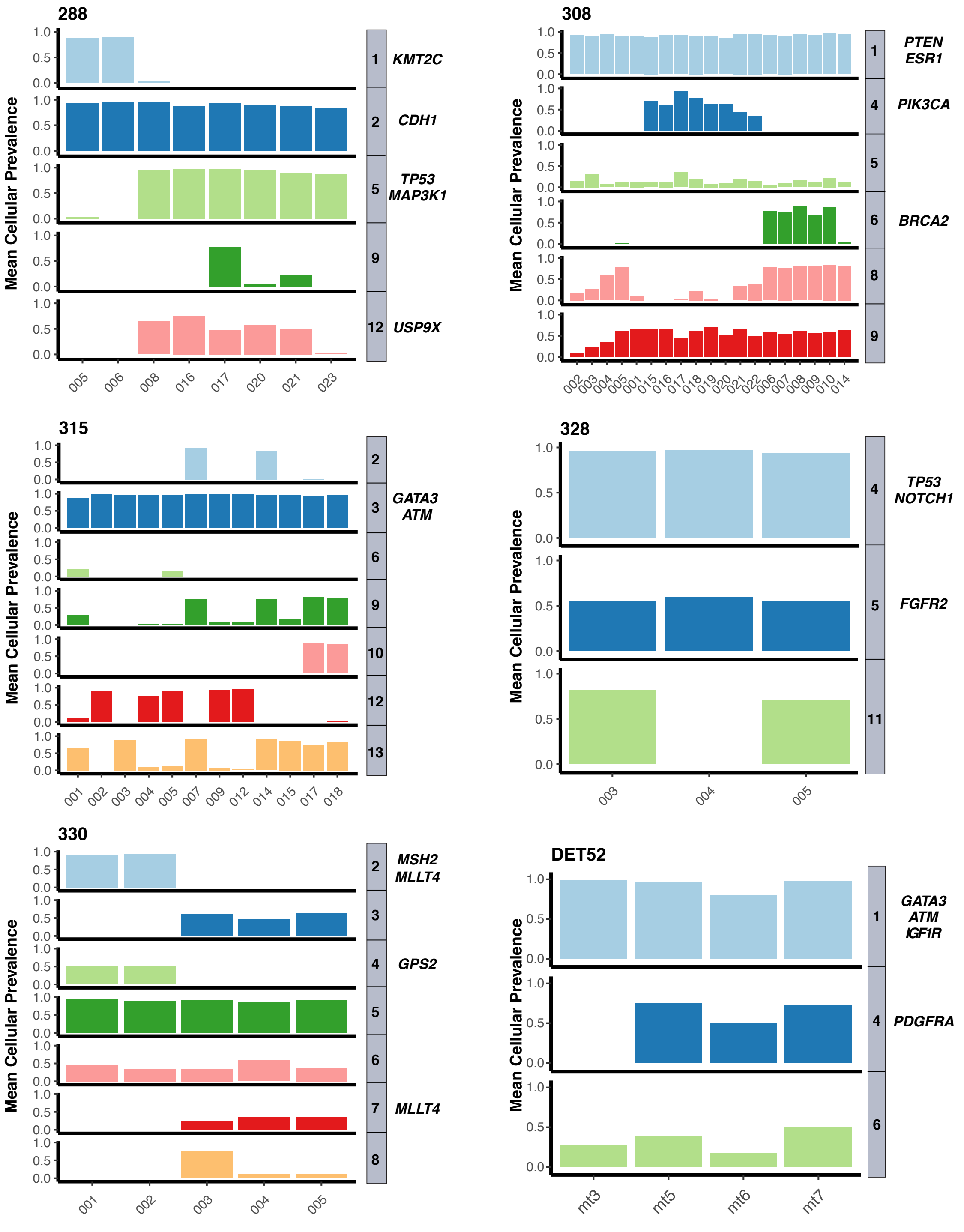

Figure S3: Mean cellular prevalence (CP) of mutation clusters identified by PyClone from WES data across metastases for 6 cases having more than two metastatic samples, related to Figure 3.

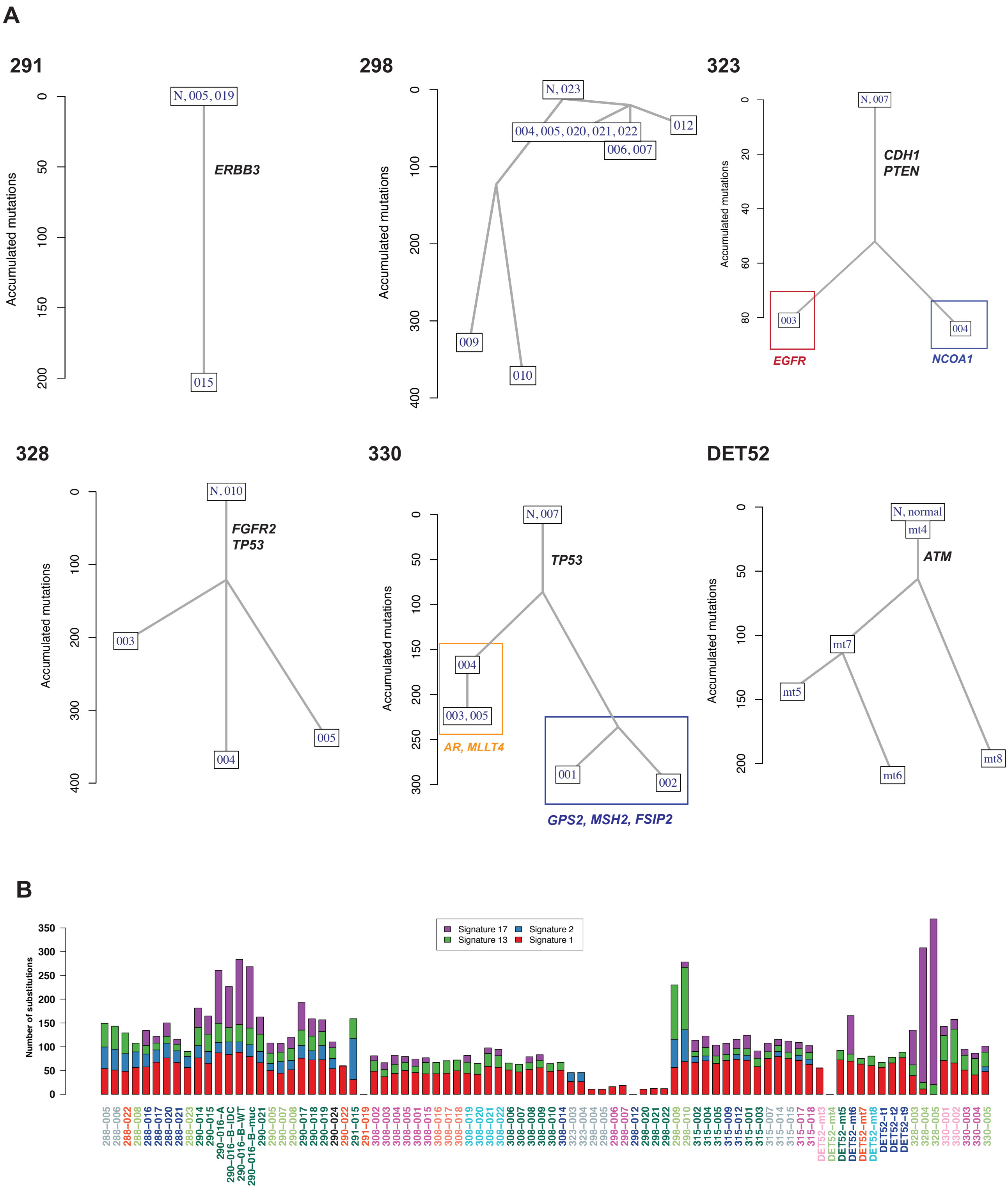

Figure S4: <u>Phylogenetic ancestries of breast cancer metastasis,</u> related to Figure 4. **(A)** Phylogenetic trees from OncoNEM algorithm. Cases 291, 298, 323, 328, 330, DET52. **(B)** Barplots of mutational signatures across metastasis for all cases with all mutations included. We only considered assignments with a minimum of ten mutations and with greater than 3% of the total mutational burden of each sample.

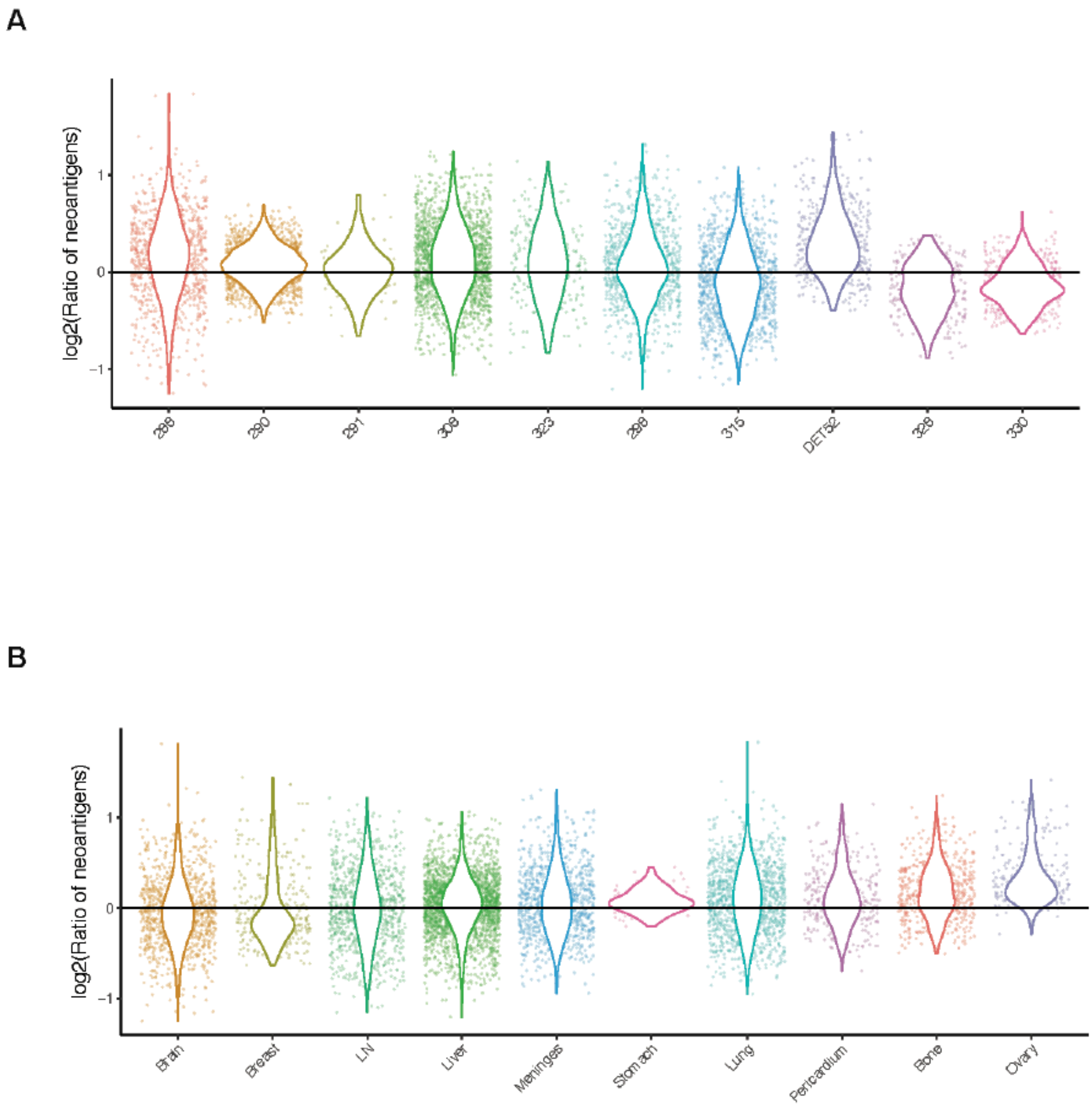

Figure S5: Neo-antigen landscape across breast cancer metastasis, related to Figure 5. **(A)** Violin plots of observed/expected neoantigen ratios grouped by patient. **(B)** Violin plots of observed/expected neoantigen ratios grouped by metastatic site.

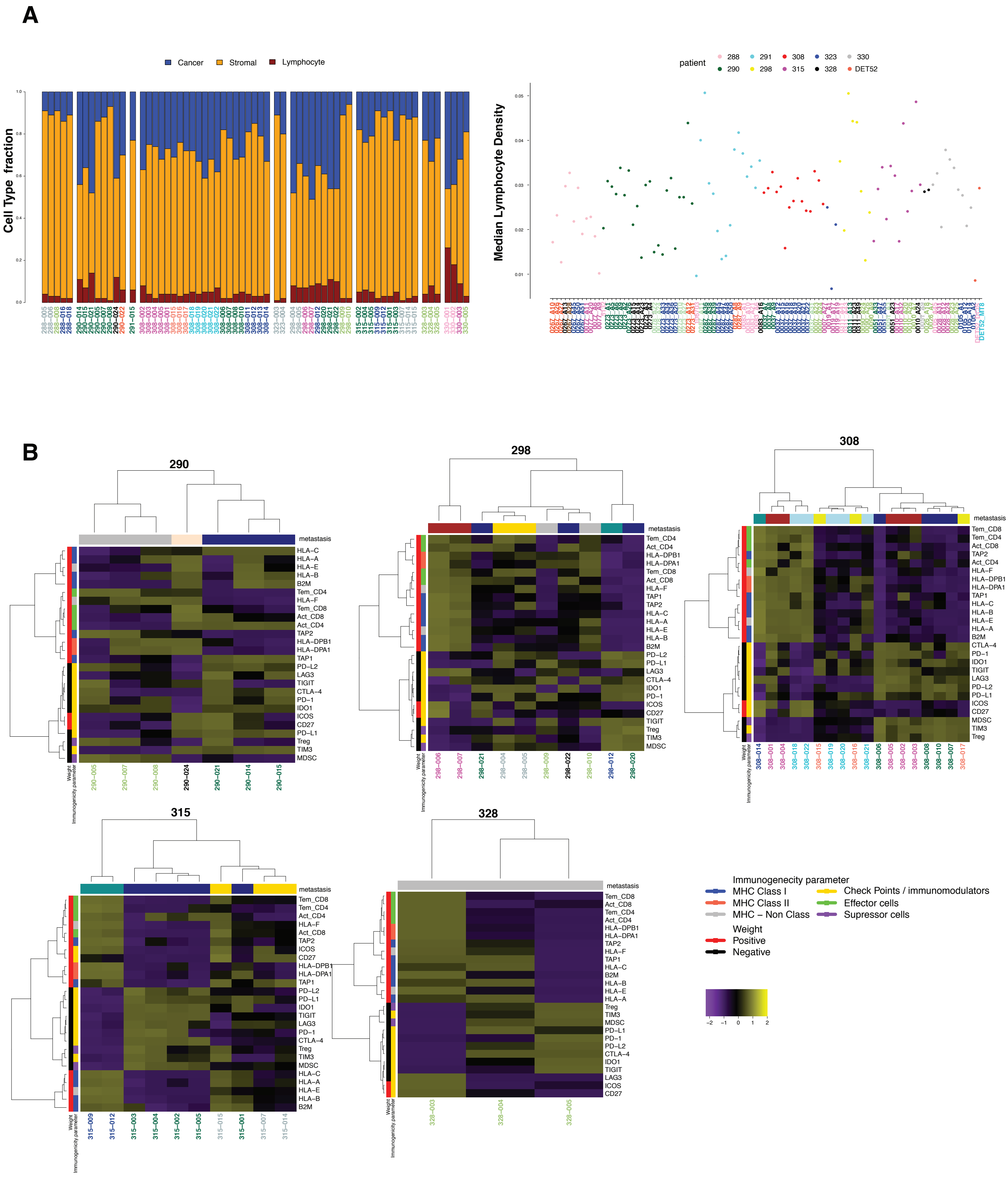

Figure S6: Tumor microenvironment is heterogeneous across metastasis, related to Figure 6. **(A)** Distribution of cancer, stromal and lymphocyte fractions across each metastasis (64 fresh frozen metastases) (left panel); Median lymphocyte density across FFPE-derived samples (primary breast and metastases) (right panel). **(B)** Heatmaps depicting two-way unsupervised hierarchical clustering of immune parameters and metastases for patients 290, 298, 308, 315, 328.

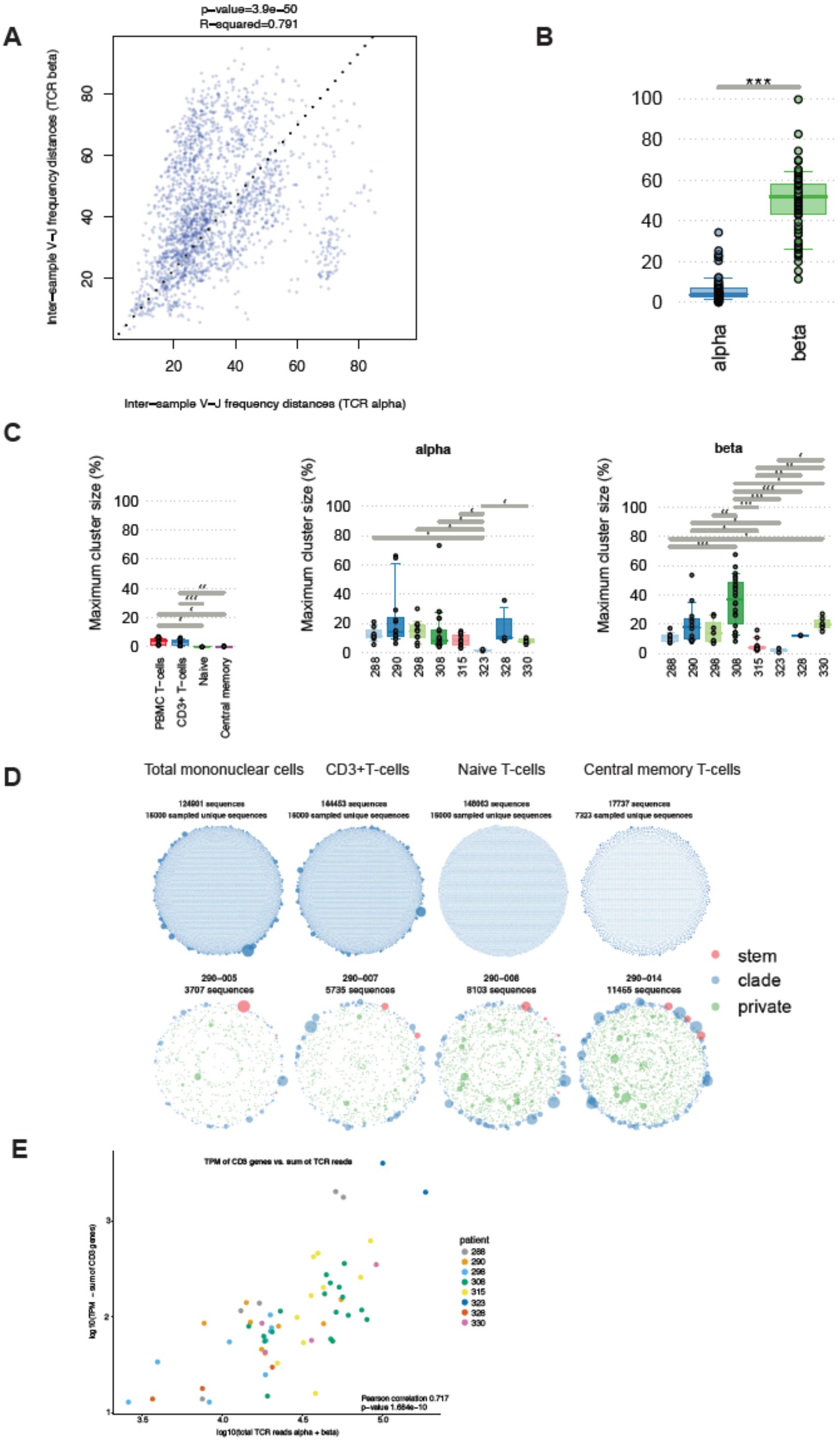

Figure S7: Analysis of TCR repertoire across metastasis, related to Figure 7. **(A)** Scatter plot of inter-sample V-J frequency distances for beta vs alpha TCR chains. **(B)** Boxplots of percentage of non-public sequences across all metastases for alpha and beta TCR chains. **(C)** Boxplots of distribution of the percentage of maximum cluster size in normal samples (beta chain) and metastases (alpha and beta chains). Significant differences between repertoire clonalities per patient for alpha and beta chains. * denotes p-values <0.05, ** denotes p-values <0.005, *** denotes p-values <0.0005 (after correcting for multiple testing). **(D)** Network plots of TCR beta chain sequences from healthy donor peripheral blood (top) and in eight metastases from case 290 (bottom). Color code indicates whether TCR sequences are stem, clade or private. Network plots for all metastases across cases where TCR sequencing was done are presented in SI7. **(E)** Correlation between the sum of the TPM values for the four genes that comprise the CD3 complex (CD3D, CD3G, CD3E and the zeta chain CD247) and the number of TCR reads.
